# Cryo-EM Structure of the Fork Protection Complex Bound to CMG at a Replication Fork

**DOI:** 10.1016/j.molcel.2020.04.012

**Published:** 2020-06-04

**Authors:** Domagoj Baretić, Michael Jenkyn-Bedford, Valentina Aria, Giuseppe Cannone, Mark Skehel, Joseph T.P. Yeeles

**Affiliations:** 1 Laboratory of Molecular Biology, Medical Research Council, Cambridge CB2 0QH, UK

**Keywords:** Fork Protection Complex, Replisome, CMG helicase, DNA replication, Genome Stability, Csm3, Tof1, Mrc1, Timeless-Tipin, Claspin

## Abstract

The eukaryotic replisome, organized around the Cdc45-MCM-GINS (CMG) helicase, orchestrates chromosome replication. Multiple factors associate directly with CMG, including Ctf4 and the heterotrimeric fork protection complex (Csm3/Tof1 and Mrc1), which has important roles including aiding normal replication rates and stabilizing stalled forks. How these proteins interface with CMG to execute these functions is poorly understood. Here we present 3 to 3.5 Å resolution electron cryomicroscopy (cryo-EM) structures comprising CMG, Ctf4, and the fork protection complex at a replication fork. The structures provide high-resolution views of CMG-DNA interactions, revealing a mechanism for strand separation, and show Csm3/Tof1 “grip” duplex DNA ahead of CMG via a network of interactions important for efficient replication fork pausing. Although Mrc1 was not resolved in our structures, we determine its topology in the replisome by cross-linking mass spectrometry. Collectively, our work reveals how four highly conserved replisome components collaborate with CMG to facilitate replisome progression and maintain genome stability.

## Highlights

• Cryo-EM structure of Csm3/Tof1 and Ctf4 bound to the eukaryotic CMG helicase
• Csm3/Tof1 are positioned at the front of the replisome where they grip duplex DNA
• High-resolution views of CMG-DNA contacts suggest a mechanism for strand separation
• Mrc1 binds across one side of CMG contacting the front and back of the replisome

## Graphical Abstract

**Figure:**
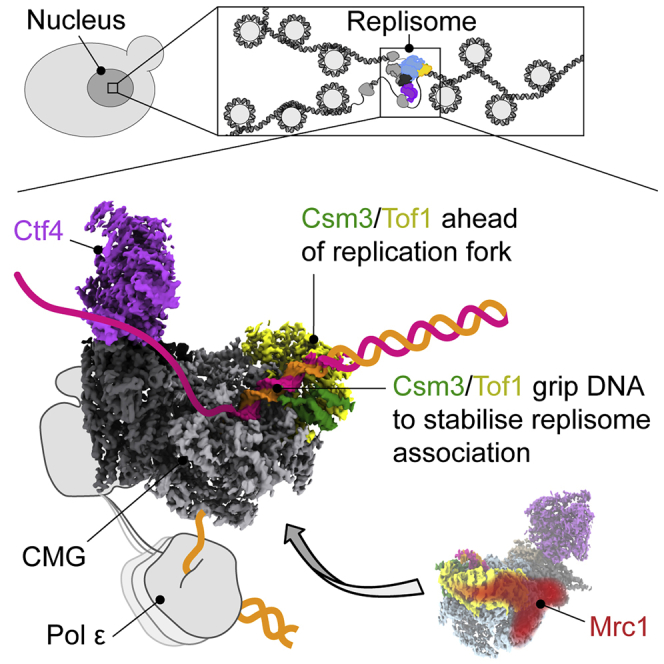

## Teaser

Baretić and Jenkyn-Bedford et al. describe cryo-EM structures of the CMG helicase bound to the fork protection complex and Ctf4 at a replication fork. They reveal the position of Mrc1 in the replisome, how Csm3/Tof1 engage CMG and DNA to facilitate fork pausing, and suggest a mechanism for strand separation.

## Introduction

Replication of eukaryotic genomes is initiated when double-hexameric minichromosome maintenance (MCM) complexes are activated to form two CMG helicases (Douglas et al., 2018, Yeeles et al., 2015). MCM is a two-tiered ring composed of six related subunits (Mcm2–7), with one tier of amino-terminal domains (N tier) and a second tier of carboxy-terminal AAA+ domains that power ATP-dependent DNA unwinding (C tier) (Bell and Botchan, 2013). Cdc45 and GINS (Go-Ichi-Ni-San) are loaded onto MCM by the combined action of multiple “firing factors,” where they stabilize the N tier through interactions with Mcm2, 3, and 5 (Costa et al., 2011, Yuan et al., 2016). CMG travels in an N tier-first orientation (Douglas et al., 2018, Georgescu et al., 2017), unwinding DNA by translocating 3ʹ→5ʹ along the leading-strand template, which is threaded through the central channel of MCM while the lagging-strand template is excluded (Fu et al., 2011). Translocation is proposed to occur via a non-symmetric rotary mechanism with ATP binding promoting single-stranded DNA (ssDNA) engagement by loops in the MCM C tier (Eickhoff et al., 2019). Despite the importance of these ssDNA contacts, they are yet to be observed in CMG structures at high resolution. Moreover, the mechanism by which ssDNA translocation is coupled to strand separation is not fully resolved.

We previously showed that robust DNA synthesis in a reconstituted system requires only the proteins necessary for CMG activation, together with primase and DNA polymerases (Yeeles et al., 2015, Yeeles et al., 2017). However, these minimal replisomes exhibited partial functionality due to an absence of critical accessory proteins (Yeeles et al., 2017), many of which are essential for proper replication fork progression and maintenance of genome stability. These include Ctf4 (And-1), Mrc1 (Claspin), and Csm3/Tof1 (Tipin/Timeless), which were identified as components of replisome progression complexes—stable CMG-containing complexes isolated from S phase yeast cells (Gambus et al., 2006). Ctf4 is a trimeric hub that recruits proteins involved in sister chromatid cohesion, ribosomal DNA maintenance, parental histone transfer, and gene silencing (Evrin et al., 2018, Gan et al., 2018, Samora et al., 2016, Simon et al., 2014, Villa et al., 2016). Csm3/Tof1 and Mrc1 are key replisome modulators, collectively termed the “fork protection complex” (FPC), that intimately associate with one another, both physically and functionally (Bando et al., 2009, Katou et al., 2003). They are essential for normal replication rates (Petermann et al., 2008, Somyajit et al., 2017, Szyjka et al., 2005, Tourrière et al., 2005, Yeeles et al., 2017), maintaining coupling of DNA synthesis to CMG in response to hydroxyurea (HU) (Katou et al., 2003), limiting trinucleotide-repeat instability (Gellon et al., 2019), and fully activating the S phase checkpoint (Alcasabas et al., 2001, Foss, 2001).

Currently, very little is known about how these functions are accomplished. Understanding their mechanisms has been hindered by a lack of structures for these proteins in the context of a replisome. In fact, there are no structures of Mrc1 or Csm3, and only an incomplete structure of the N terminus of the human Tof1 ortholog, Timeless, in isolation (Holzer et al., 2017). To address these issues, we have determined the electron cryomicroscopy (cryo-EM) structure of a 1.4 MDa complex comprising CMG, Ctf4, and the FPC at a replication fork.

## Results

### Cryo-EM Structure of Csm3/Tof1, Mrc1, and Ctf4 in Complex with CMG at a Replication Fork

**Figure 1:**
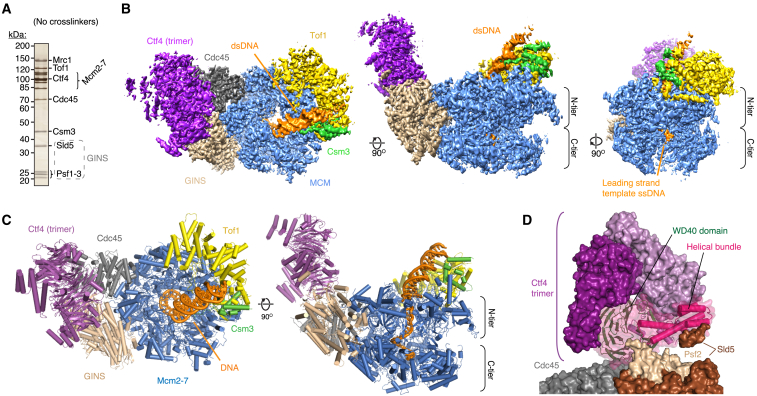
Structure of CMG Bound to Csm3/Tof1, Ctf4, and a DNA Fork (A) Silver-stained SDS-PAGE of a representative glycerol gradient fraction of a non-cross-linked sample (fraction 11, Figure S1B) equivalent to fractions used for cryo-EM. (B and C) Cryo-EM density map (B) and corresponding atomic model (C) of a complex assembled as in Figure S1A observed in conformation 1. (D) Model of the Ctf4 trimer bound across the Cdc45-GINS interface of CMG, rendered as a surface. The Ctf4 monomer mediating the interaction with CMG is rendered also as a cartoon.

To assemble complexes for cryo-EM, we incubated *S. cerevisiae* CMG with fork DNA, Ctf4, Csm3/Tof1, Mrc1, and the non-hydrolyzable ATP analog adenylyl-imidodiphosphate (AMP-PNP) (Figure S1A). Analysis of complex formation over glycerol gradients revealed Csm3/Tof1, Mrc1, and Ctf4 co-sedimenting with CMG (Figures 1A and S1B). Previous work established that Tof1 phosphorylation promotes its association with CMG (Bastia et al., 2016). Consistent with this finding, Tof1 was phosphorylated in our Csm3/Tof1 preparation (Figure S1C). Samples for cryo-EM were prepared following glycerol gradient fixation (Kastner et al., 2008), yielding three-dimensional (3D) reconstructions that enabled model building of CMG, the homotrimeric Ctf4 C terminus, and ∼900 residues of the Csm3/Tof1 heterodimer and fork DNA (Figures 1B, 1C, S1, and S2; Tables 1 and S1). In addition to assembling complexes by reconstitution, we also determined cryo-EM reconstructions of the same protein complex prepared following co-overexpression of all 15 proteins in *S. cerevisiae* (Figures S3A–S3G), demonstrating there are no major differences in the architecture of Csm3/Tof1 and Ctf4 bound to CMG between the different assembly methods (Figure S3H). Furthermore, we also examined the structure of a complex prepared by co-expression in the absence of cross-linking; although the sample displayed considerable heterogeneity, we obtained a 3D class containing Ctf4 and Csm3/Tof1 bound to CMG (Figure S3I). Docking the atomic model derived from the reconstituted sample into this cryo-EM map illustrates that gradient fixation did not alter the positioning of Csm3/Tof1 or Ctf4 when bound to CMG.

**Table 1:** Cryo-EM Statistics for the Reconstituted Sample, Related to Figure 1

	Conformation 1 (CMG-Csm3-Tof1-Ctf4-Fork DNA) (EMDB: EMD-10227; PDB: 6SKL)	Conformation 2 (MCM C Tier, ssDNA) (EMDB: EMD-10230; PDB: 6SKO)
**Data Collection and Processing**		

Grids	Cu R2/2 400 mesh (Quantifoil) with continuous carbon support	Cu R2/2 400 mesh (Quantifoil) with continuous carbon support
Cryo-specimen freezing	Manual plunger	Manual plunger
Microscope	Titan Krios (Thermo Fisher)	Titan Krios (Thermo Fisher)
Detector	K2 Summit (Gatan)	K2 Summit (Gatan)
Datasets	2	2
Micrographs (used in processing)	6,682 (20 frames per micrograph)	6,682 (20 frames per micrograph)
Voltage (keV)	300	300
GIF energy filter slit width (eV)	20	20
Electron exposure (e^−^/Å^2^)	37	37
Defocus range (μm)	−1.4 to −2.6	−1.4 to −2.6
Sampling interval (Å/pixel)	1.049	1.049
Initial particle number	632,077	632,077
Final particle numbers	Csm3/Tof1 (MBR maps): 282,761; CMG + Ctf4 (MBR maps): 198,120; whole complex (single map): 34,647	181,957
Map resolution (Å): 0.143 FSC threshold	3.1–3.7	3.2–3.7

**Refinement**		

Model resolution (Å): 0.5 FSC threshold	4.1	3.8
Map-sharpening B factor (Å^2^)	−5	−5 or −20
Model composition		
Non-hydrogen atoms	59,184	15,715
Protein residues	7,251	1,955
Ligands	3 AMP-PNP, 3 Mg^2+^, 5 Zn^2+^	5 AMP-PNP, 5 Mg^2+^
RMS deviations		
Bond lengths (Å)	0.70	0.70
Bond angles (°)	1.13	1.19
Validation^a^		
MolProbity score	0.81	1.19
Clashscore	0.26	0.95
Poor rotamers (%)	0.09	0.24
Ramachandran plot		
Favored (%)	96.71	94.01
Allowed (%)	3.29	5.99
Outliers (%)	0.00	0.00

For details about the derivation of individual maps used in model building and refinement and their respective resolutions quoted at the 0.143-FSC threshold, refer to Figure S2 and STAR Methods. Electron Microscopy Data Bank (EMDB) accession codes are those with the most complete density across all residues in the Protein Data Bank (PDB) models; for EMDB accession codes of additional maps used in model building, refer to Figure S2 and Key Resources Table. FSC, Fourier shell correlation; GIF, Gatan imaging filter; MBR, multi-body refinement; RMS deviation, root-mean-square deviation.

a MolProbity validation server (http://molprobity.biochem.duke.edu/).

Overall, the complex displays a “horseshoe-like” configuration, with the discoidal Ctf4 trimer sitting across the GINS-Cdc45 interface whereas Csm3/Tof1 are located on the N tier face of MCM (Figures 1B–1D). Two turns of double-stranded DNA (dsDNA) protrude from the N tier of the MCM central channel, tilted from the vertical axis of the channel toward Csm3/Tof1, where it contacts both proteins. We observe two major conformations for the MCM C tier (termed conformations 1 and 2) where the whole C tier swings beneath the N tier, with some inter-subunit motion between adjacent C tier AAA+ domains (Figures S1I–S1K; Video S1). No major differences in the MCM N tier or other subunits were observed between the two conformations (Figure S1L). Compared to the reconstituted dataset, distinct conformations were not distinguished for the C tier in co-expressed samples, resulting in the C tier being considerably less well resolved, likely owing to heterogeneity in co-purifying DNA fragments and/or a lack of nucleotide during sample preparation (Figures S3G and S3I). Finally, comparison of conformation 1 with a prior CMG-DNA structure illustrates binding of the FPC and Ctf4 did not significantly alter the global conformation of CMG, although several small regions became ordered upon accessory factor binding (Figure S1M).

Video S1 Comparison of MCM Subunits in Conformations 1 and 2, Related to Figures 3 and S1 Movie shows morphing between models adjusted to the cryo-EM density maps of the complex in conformation 1 and conformation 2. The large global repositioning of the MCM C-tier (lower) relative to the N-tier (upper, binding dsDNA) is clear. The map for conformation 2 was that with 5 AMP-PNP molecules bound. To generate the movie, models for each conformation were aligned on MCM N-tier regions using PyMOL before morphing between these models using Chimera.

### Cross-Linking Mass Spectrometry Reveals the Location of Mrc1 in the Replisome

Although the level of Mrc1 incorporation was comparable to other components (e.g., Tof1) in both reconstituted and co-expressed samples (Figures 1A, S1B, and S3A), we did not observe significant density that could be assigned to the 125 kDa protein in either dataset (Figures 1B and S3G). We did, however, observe disconnected helical densities across one side of the complex in samples prepared both by reconstitution and co-expression, specifically associated with Tof1, Mcm6, Mcm2, and Cdc45 (Figure S3J). Although the sequence identity of these densities could not be determined, we considered that some of them might be contributed by Mrc1. In support of this, Mrc1 is known to interact with Csm3/Tof1 (Bando et al., 2009, Lewis et al., 2017) positioned at the front of the replisome, as well as the Mcm6 winged helix and DNA polymerase (Pol) ε (Komata et al., 2009, Lou et al., 2008), both of which travel behind CMG (Goswami et al., 2018, Sun et al., 2015). Mrc1 might therefore stretch from the front to the rear of the replisome.

**Figure 2:**
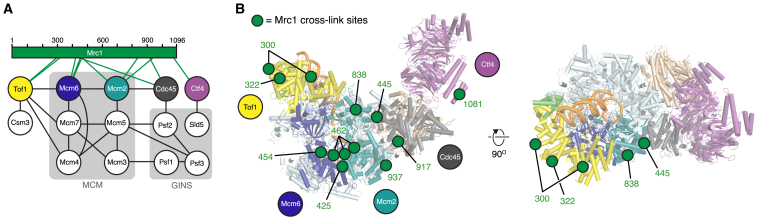
XL-MS Identifies the Position of Mrc1 in the Eukaryotic Replisome (A) Summary of cross-linking mass spectrometry (XL-MS) for a co-expressed replisome subcomplex (see Table S2 for details of all inter- and intra-subunit cross-links). Lines indicate inter-subunit cross-links. (B) Positions of Mrc1 cross-links detected in XL-MS mapped to the structure of CMG-Csm3-Tof1-Ctf4. The positions of residues observed to cross-link with Mrc1 are indicated by green circles; green labels indicate which Mrc1 residues cross-link to these sites.

To investigate the location of Mrc1 in the replisome and further validate our cryo-EM structures, we performed cross-linking mass spectrometry (XL-MS) using disuccinimidyl dibutyric urea (DSBU) that cross-links reactive amino acids within ∼30 Å (Merkley et al., 2014). This approach yielded numerous cross-links between CMG subunits, over 90% of which could be rationalized on the CMG structure, validating the stringency of this approach (Figure S3K). Moreover, all cross-links between Tof1 and MCM subunits or Csm3 involved solvent-exposed residues located within 30 Å, further highlighting the consistency between cryo-EM and XL-MS approaches (Figures S3L and S3M). We identified 11 distinct cross-links involving Mrc1 and other subunits of the complex (Figure 2A; Table S2). Notably, all 11 cross-links localized to one side of CMG (Figure 2B). The N terminus of Mrc1 cross-linked to Tof1, whereas a cluster of cross-links involving the Mrc1 mid-region was observed at the C tier by Mcm6/Mcm2, and several cross-links involving more C-terminal regions of Mrc1 were detected on Cdc45 and Ctf4 (Figure 2; Table S2). These data indicate Mrc1 contacts CMG in multiple locations across one side of the complex, extending from its N-terminal association with Tof1 to its C-terminal association in the vicinity of Cdc45, close to the binding site for the Pol ε non-catalytic domain (Goswami et al., 2018), with which the Mrc1 C terminus is proposed to interact (Lou et al., 2008).

### Structure of Ctf4 in the Replisome

The region of Ctf4 density we resolve is very similar to crystal structures of Ctf4 and And-1 C-terminal domains (Guan et al., 2017, Kilkenny et al., 2017, Simon et al., 2014) and a recent cryo-EM structure of CMG-Ctf4 lacking DNA (Yuan et al., 2019) (Figures S4A and S4B), and therefore neither the FPC nor the replication fork alters the position of Ctf4 in the replisome. The trimer is rigidly bound, sitting side-on across the interface between Cdc45 and the GINS subunit Psf2, with the helical domains facing away from the replication fork (Figures 1C, 1D, and S1L, left). One Ctf4 monomer mediates the interaction with CMG, burying 520 Å^2^ of Cdc45 and 790 Å^2^ of Psf2, primarily involving two blades of its WD40 domain (Figures S4C–S4G). We do not observe clear density for the ∼400-residue (1–383) Ctf4 N terminus predicted to contain a WD40-like domain (Guan et al., 2017), consistent with it being loosely connected to the C terminus (Simon et al., 2014) and not stabilized by DNA or the FPC in our structure. Because dsDNA is bent away from Ctf4 toward Csm3/Tof1 at the front of the replisome, partner proteins that dock onto the replisome via Ctf4 may be located a considerable distance from the parental DNA duplex approaching the fork junction. This may be of particular significance for Pol α, which is involved in parental H3-H4 transfer to the lagging strand in conjunction with the N-terminal extension (NTE) of Mcm2 (Gan et al., 2018) (Figure S4H).

### High-Resolution Details of CMG-ssDNA Interactions in the MCM AAA+ C Tier

**Figure 3:**
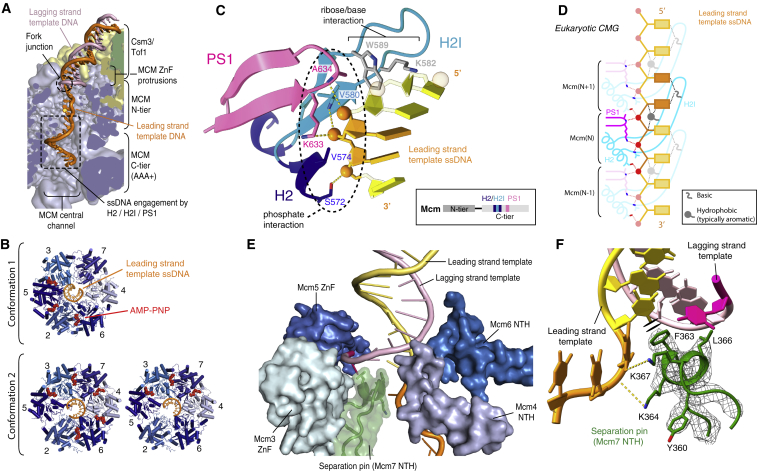
Interaction of Eukaryotic CMG Helicase with Fork DNA (A) Cutaway showing the path of DNA approaching and traversing the MCM central channel in conformation 1. (B) Comparison of the MCM C tier between conformations 1 and 2 (subclasses bound to five or three AMP-PNP molecules are shown). (C) Individual MCM ssDNA-binding motif (Mcm2 shown). Three phosphates contacted by the single MCM subunit are colored orange. Ribose and/or base contacts observed in most but not all subunits (see Figures S5A–S5C and S5G). Inset: locations of the ssDNA-binding loops in the MCM primary sequence. (D) Schematic demonstrating the repeating nature of MCM-ssDNA contacts. For variations in sugar/base contacts, see Figure S5G. Bolder colors highlight the ssDNA-binding motif of a single MCM subunit. Phosphates are colored red. (E) MCM N tier loops contacting DNA around the fork junction. Loops are rendered as surfaces, with the Mcm7 NTH separation pin also represented as a cartoon. For (D) and (E), unpaired ssDNA is colored darker pink/orange for the lagging- and leading-strand template, respectively. (F) Detailed view of the strand-separation pin displayed in cryo-EM density (mesh), inserting between the two strands of DNA at the point of unwinding. F363 makes π-π interactions with DNA. ZnF, zinc finger; H2, helix 2; H2I, helix 2 insertion loop; PS1, presensor 1; NTH, N-terminal hairpin.

Despite the presence of the FPC and Ctf4 in our structure, we observe two C tier configurations similar to two conformational states previously determined at lower resolution for isolated *Drosophila* CMG in the presence of ATP (Eickhoff et al., 2019) (conformations 1 and 2; see Figures S1I–S1L and Video S1). These two states were reported to represent CMG translocation intermediates, enabling CMG-ssDNA contacts made during translocation to be inferred from our structures. Conformations 1 and 2 differ in ATPase site occupancy and ssDNA engagement by the presensor 1 (PS1) hairpin and helix 2 (H2)/helix 2 insertion (H2I) loop that protrude into the MCM central channel (Figures 3A and 3B). In both conformations, MCM subunits contact backbone phosphates using four highly conserved residues; every other phosphate is coordinated by both a PS1 lysine and the backbone amide of an H2I valine/isoleucine from a single MCM, whereas each remaining phosphate is bound by an H2 serine and PS1 alanine from two neighboring subunits (Figures 3C, 3D, and S5A–S5E). This establishes a highly repetitive arrangement whereby every other phosphate is coordinated in an equivalent manner maintaining a two-phosphate periodicity as observed in homohexameric archaeal MCM (Meagher et al., 2019) (Figure S5F, far left). Although the precise ssDNA contacts and number of phosphates bound per subunit vary among diverse hexameric helicases, the formation of repetitive backbone phosphate contacts is universal (Figure S5F).

In addition to the phosphate backbone contacts, the H2I loops of several MCM subunits interact with ribose and bases primarily through residues in two conserved positions; in Mcm2, 5, 6, and 7, a basic residue and an aromatic/methionine side chain positioned close to one another mediate these interactions (Figures 3C, 3D, S5A–S5C, and S5G). In Mcm3, the equivalent basic residue is absent whereas an arginine (R455) replaces the aromatic/hydrophobic residue (Figures S5C and S5G). These contacts are similar between the two conformations, except Mcm7 is disengaged in conformation 1, Mcm2 differs in its contacts in conformation 2 as the path of ssDNA diverges toward the 5ʹ end, and the position of Mcm3/5 near the 5ʹ end and Mcm2/6 nearer the 3ʹ end in conformation 1 is reversed in conformation 2 (Figure S5G). The equivalent Mcm4 basic/hydrophobic pair is disengaged from DNA in both conformations, with a unique tyrosine (Y604) instead contacting DNA in conformation 2 (Figures S5B, S5C, and S5G). Additional basic residues at a distinct position in the H2I loops of Mcm5 (R460) and Mcm2 (K587) also project toward the fork junction in conformation 1, likely contacting bases as the ssDNA is translocated from the N tier toward the C tier (Figures S5C and S5G, top), whereas for conformation 2 it is the Mcm2 W589 that contacts bases as the path of ssDNA diverges (Figures S5B and S5G, bottom); these contacts might be important to guide ssDNA along the correct path from the fork junction toward engagement by the MCM motor domains.

### A Mechanism for Strand Separation by the Replisome

ssDNA translocation by the C tier motor domains must be coupled to dsDNA unwinding during replication and the high resolution of our structure at the fork junction suggests a mechanism for how this is accomplished. Consistent with lower-resolution structures (Georgescu et al., 2017, Goswami et al., 2018), strand separation occurs after dsDNA enters the circle of zinc-finger (ZnF) domains on top of the N-terminal face of the MCM ring (Figure 3A). The position of the fork junction is not significantly influenced by the association of Csm3/Tof1 (Figure S6A). Multiple structural features contact DNA in the vicinity of the fork junction, several of which require extensive remodeling from their positions in the MCM double hexamer (Figures 3E and S6B–S6D; Video S2) (Abid Ali et al., 2017, Li et al., 2015, Noguchi et al., 2017). This remodeling enables the N-terminal hairpins (NTHs) of Mcm6 and Mcm4 to engage the lagging-strand template ahead of strand separation, where they help guide the incoming dsDNA onto the NTH of Mcm7, which appears to function as a separation pin, splitting the two strands of the incoming duplex (Figure 3F). This configuration of the Mcm7 NTH is observed in both conformations 1 and 2 (Figure S6C). The Mcm7 NTH itself undergoes extensive rearrangement from the double hexamer, where it is engaged with the Mcm5 ZnF of the second hexamer (Figure S6D) (Abid Ali et al., 2017, Li et al., 2015, Noguchi et al., 2017); the lack of this second Mcm5 ZnF in the active helicase enables both the DNA and Mcm7 NTH to reposition, such that the Mcm7 NTH can insert between the two strands of DNA at the fork junction. Furthermore, it remodels to form a helical turn at its apex, positioning an invariant phenylalanine to form π-π interactions with the last base pair in the DNA (Figures 3E, 3F, and S6B–S6E; Video S2). Positioning hydrophobic residues to appose the last base pair is a hallmark of separation pins in diverse helicases, including the T7 replisome (Figure S6F) (Gao et al., 2019). Two lysines from the Mcm7 NTH that previously interacted with the *trans*-Mcm5 ZnF in the double hexamer now contact the unwound leading-strand template, at which point ssDNA is kinked almost 90° before continuing toward the C tier (Figures 3F, S6A, S6B, S6D, and S6E; Video S2). We propose this arrangement of intricate DNA contacts surrounding the fork junction functions to destabilize the DNA duplex and block entry of the lagging-strand template to the MCM central channel while the leading-strand template is translocated through.

Video S2 Overview of MCM N-Tier Interactions with DNA at the Fork Junction with the Residues Involved in the Direct Interaction Highlighted, Related to Figures 3 and S6 Details of the Mcm7 NTH strand-separation pin are shown at the end. Movie produced using PyMOL.

After strand separation, a single nucleotide is clearly resolved for the unwound lagging-strand template that runs above the Mcm7 NTH, approaching the Mcm3 and Mcm5 ZnFs (Figure 3E; Video S2). However, it is not resolved beyond this point despite the fork substrate containing 14 additional nucleotides of ssDNA (Figure S1A), consistent with prior lower-resolution CMG structures (Georgescu et al., 2017, Goswami et al., 2018). Based on the position of the final nucleotide resolved in our structures, we consider it most likely the lagging-strand template will exit between the ZnF domains of Mcm3/5 or Mcm5/2 (Figure S6G).

### Structure of Csm3/Tof1

**Figure 4:**
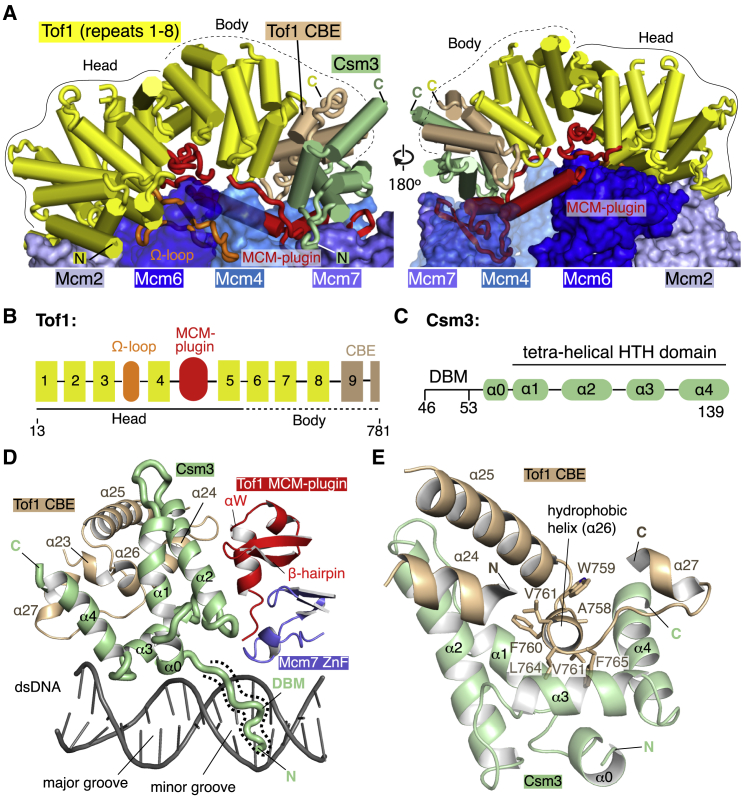
Csm3/Tof1 Structure (A) Structures of Tof1 and Csm3 shown as cylinders above the MCM N tier (surface representation). Tof1 insertions (cartoon representation): the Ω-loop (orange) and the MCM-plugin (red) are highlighted. The Csm3-binding element (CBE) of Tof1 is colored brown. The positions of the Tof1 head and body are outlined with solid and dashed black lines, respectively. For clarity, dsDNA is not shown. (B) Schematic illustrating the positions of Tof1 helical repeats (numbered 1–9; see Figure S7A for repeat assignment) and Tof1 features (CBE, Ω-loop, and MCM-plugin). The head and body subdivisions are marked with solid and dashed black lines, respectively. (C) Schematic illustration of Csm3 domain architecture with helices α0–α4 labeled. (D) Overview of the Csm3 structure (46–139) and its interface with Tof1 and the Mcm7 ZnF. The Csm3 DNA-binding motif (DBM) is highlighted by a dashed outline. (E) Overview of interactions between Csm3 and the Tof1 CBE. Hydrophobic residues from Tof1 helix α26 are shown.

Before the template is unwound, dsDNA projects toward Csm3/Tof1 at the front of the replisome (Figures 1B, 1C, and S6A). Tof1 (13–781) forms a right-handed α solenoid with a crescent-like architecture that mimics the curvature of the MCM ring, traversing the N tier face above Mcm2, 6, 4, and 7 (Figures 1B, 1C, and 4A; Video S3). The α solenoid is composed of nine tandem 2- or 3-helix units resembling either armadillo or HEAT repeats, which can be subdivided into a head (repeats 1–5) and body (repeats 6–9) (Figures 4A, 4B, and S7A). The first six repeats superpose well with the crystal structure of the N terminus of human Timeless (Holzer et al., 2017), whereas repeats 5–9 are reminiscent of p120 catenin (Ishiyama et al., 2010) (Figures S7B and S7C). Clear density for the C-terminal third of Tof1 is not observed, indicating it is flexibly tethered. Two large insertions absent from the Timeless crystal structure (Holzer et al., 2017) embellish the α solenoid: a 35-residue Ω-loop and an ∼100-amino acid region, hereafter referred to as the “MCM-plugin,” that extends in a large loop over the surface of the MCM N tier encircling the base of the Tof1 body (Figures 4A, 4B, S7D–S7F, and S8A).

Video S3 Overview of the Structure of Csm3/Tof1 Demonstrating the Positions of the Tof1 Ω Loop, MCM Plugin, and Csm3-Binding Element and Showing the Binding of Csm3 to Tof1, Related to Figures 4 and S7 Movie produced using PyMOL.

We resolve 94 residues of Csm3 comprising five α helices, which fold around a hydrophobic core to form a tetra-helical helix-turn-helix (HTH) domain (α1–4), with a short DNA-binding motif (DBM) preceding α0 (Figures 4A, 4C, 4D, and S8B). The Csm3 HTH encases a hydrophobic helix (α26) at the C-terminal end of the Tof1 body, part of a larger interface involving a region of Tof1 termed the “Csm3-binding element” (CBE) (Figure 4; Video S3). This extensive interface and additional contacts with the MCM plugin ensure Csm3 is correctly positioned in the replisome.

### Csm3/Tof1 Interactions with MCM and dsDNA

**Figure 5:**
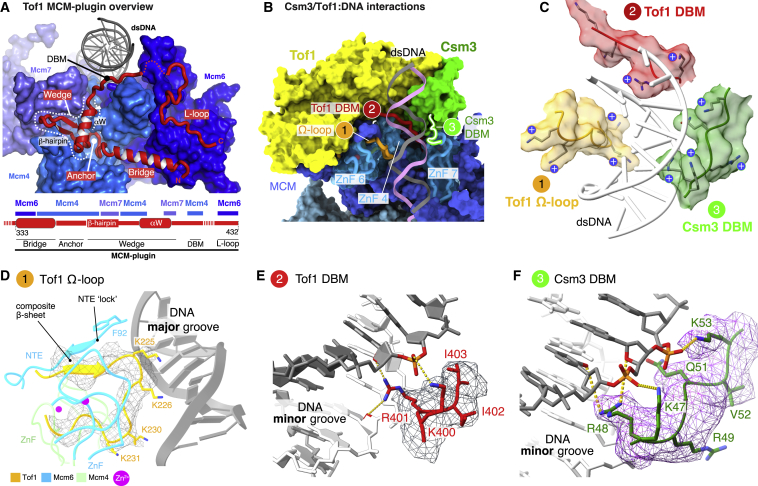
Tof1 Interactions with MCM and DNA (A) Overview of the Tof1 MCM-plugin (red) and its position on the MCM N tier. Top: the MCM-plugin is shown in cartoon representation above the MCM N tier (surface representation) and structural elements involved in MCM binding are illustrated, as is the location of a DBM. Bottom: schematic illustrating the organization of the MCM-plugin. Structural elements involved in MCM binding are illustrated, together with the specific Mcm subunits that they bind. (B) Overview of Csm3/Tof1 dsDNA contacts at the front of the replisome. The Mcm4, 6, and 7 ZnF domains important for Csm3/Tof1 binding are displayed as cartoons in transparent surfaces. (C) Close-up view of the Csm3/Tof1 dsDNA grip. For simplicity, only the Ω-loop and DBMs are shown. (D) Detailed view of Ω-loop interactions with Mcm6, Mcm4, and dsDNA. Cryo-EM density for the Ω-loop is shown as mesh. (E and F) Detailed views of the Tof1 (E) and Csm3 (F) DBMs with the cryo-EM density shown as mesh.

In our structure, Csm3/Tof1 binds to MCM almost exclusively via Tof1. Although the N terminus of the α solenoid forms an interface with the NTE of Mcm2 (Figure S9A), binding is mediated primarily by the MCM-plugin and Ω-loop (Figures 5A and S7F; Video S4). The MCM-plugin contains four structural features (Figure 5A; bridge, anchor, wedge, and L-loop). The bridge comprises an α helix spanning the helical domains of Mcm6 and Mcm4, making contacts with Mcm6 through a small conserved hydrophobic patch flanked by two glutamates (Figures 5A, S8A, and S9B). The bridge is anchored on Mcm4 by a short loop (anchor) positioned in a cleft between the helical domain, ZnF, and OB fold of Mcm4 (Figures 5A, S8A, and S9C). The MCM-plugin then stretches upward into a β hairpin and short helix αW (collectively the “wedge”) sandwiched between regions of Mcm4 and Mcm7 and beneath Csm3 (Figures 4A, 4D, 5A, and S9D). Finally, it returns toward the head, contacting dsDNA via a short DBM, before looping back in an L shape (L-loop) over the surface of Mcm6, where it binds both the helical domain and NTE (Figures 5A and S9E).

Video S4 Overview of the Interface Formed between Csm3/Tof1 and MCM, Related to Figures 4, 5, and S9 Movie produced using PyMOL.

Being located at the front of the replisome enables Csm3/Tof1 to “grip” the parental DNA duplex via a network of interactions with the phosphate backbone and both the major and minor grooves (Figures 5B, 5C, and S7F; Video S5). This grip embraces three-quarters of a turn of dsDNA and comprises contacts mediated by the Tof1 Ω-loop and conserved DBMs from both the Tof1 MCM-plugin and Csm3. The Ω-loop protrudes from the α solenoid toward dsDNA, slotting between the ZnF domains of Mcm6 and Mcm4 (Figures 5D, S9F, and S9G; Video S4). The Mcm6 NTE, which becomes ordered upon Tof1 association (Figure S1M), “locks” the Ω-loop in place by extending over it and using a phenylalanine to form an intra-molecular interaction with the Mcm6 ZnF, allowing a short composite β sheet to form between the NTE and Ω-loop (Figure S9F). The entire hydrophobic core of the Ω-loop packs against the Mcm6 ZnF positioned to one side, whereas considerably fewer contacts are made with the Mcm4 ZnF on the opposite side (Figure S9G). This configuration places the tip of the Ω-loop facing the major groove, where several lysine residues interact with the phosphate backbone (Figures 5D, S7F, S8A, and S9F). The equivalent loop in Timeless is considerably shorter than the Tof1 Ω-loop and therefore this mode of DNA interaction may not be universal across eukaryotes (Figure S8A).

Video S5 Overview of the Interactions between Csm3/Tof1 and the Parental dsDNA Duplex, Related to Figure 5 Movie produced using PyMOL.

The DBMs from the Tof1 MCM-plugin and Csm3 both bind the minor groove (Figures 5B and 5C; Video S5). In the Tof1 DBM (400–404), R401 is placed into the minor groove contacting sugars approximately one turn above the fork junction and is flanked by two conserved lysine residues (K400 contacting phosphate, and K404 facing the Tof1 body) (Figures 5C and 5E). The Csm3 DBM (46–53) inserts R48 into the minor groove, where it is positioned to contact both bases and ribose (Figure 5F). In addition, R46 and K47 are close to the backbone, perhaps to stabilize R48, whereas Q51 projects toward the minor-groove and K53 coordinates the backbone phosphate at the end of the DBM. This configuration is reminiscent of minor groove binding by the N-terminal arm of homeodomain transcription factors that also precedes an HTH (Figures S9H and S9I). Finally, a fourth region of Csm3/Tof1 could also bind dsDNA because several basic residues in the loop between Csm3 α3 and α4, together with the linker between Tof1 α26 and α27, are in close proximity to the backbone (Figure S9H).

### The dsDNA Grip Stabilizes Csm3/Tof1 in the Replisome

**Figure 6:**
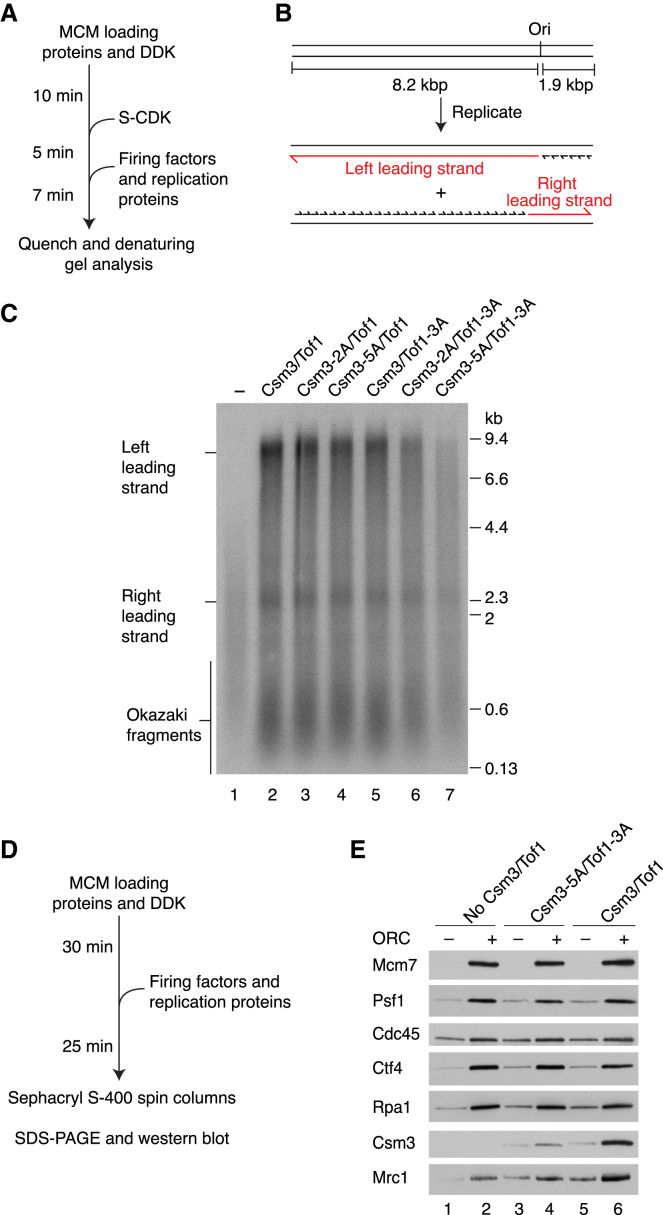
Csm3/Tof1 DNA Binding Is Important for Replisome Stability (A) Reaction scheme for origin-dependent replication assay. (B) Schematic of the DNA template and anticipated replication products. (C) Origin-dependent replication reaction (7 min) with the indicated Csm3/Tof1 proteins performed as illustrated in (A). Products were separated through a 0.6% alkaline agarose gel. (D) Reaction scheme for protein association experiments. (E) Western blot analysis of a reaction performed as in (D) with the indicated Csm3/Tof1 proteins.

We previously showed Csm3/Tof1 is required for maximal replication rates in a reconstituted system (Yeeles et al., 2017) and considered the DBMs might be important for this. Therefore, we substituted DBM residues for alanine and purified stable heterodimers of Csm3^R49A, K53A^/Tof1 (Csm3-2A/Tof1), Csm3^K47A, R48A, R49A, Q51A, K53A^/Tof1 (Csm3-5A/Tof1), Csm3/Tof1^K400A, R401A, K404A^ (Csm3/Tof1-3A), and double mutants where both subunits were mutated (Csm3-2A/Tof1-3A and Csm3-5A/Tof1-3A) (Figure S10A). Surprisingly, Figure S10B shows that all DBM mutants bound to fork DNA with comparable efficiency to the wild type, indicating the complex possesses additional DNA-binding regions, perhaps in the ∼400-amino acid C-terminal region of Tof1 and/or the C terminus of Csm3, which are not resolved in our structures. We then performed origin-dependent replication reactions (Aria and Yeeles, 2018, Taylor and Yeeles, 2018) on a linear template with the origin located to generate leading strands of 1.9 kb (right) and 8.2 kb (left) using conditions that require Csm3/Tof1 for rapid and efficient DNA replication (Yeeles et al., 2017) (Figures 6A and 6B). Figure 6C shows that mutation of either the Csm3 (2A and 5A) or Tof1 DBMs had little effect on left leading-strand products (compare lanes 3–5 with 2). In contrast, when both subunits were mutated, we observed a minor replication defect with Csm3-2A/Tof1-3A (Figure 6C, compare lanes 2 and 6) and a marked replication defect with Csm3-5A/Tof1-3A (compare lanes 2 and 7), although not as severe as Csm3/Tof1 omission, with some 8.2 kb left lead products still synthesized. At longer time points, both double mutants supported the synthesis of fully replicated left leading strands to levels above those generated in the absence of Csm3/Tof1 (Figures S10C and S10D). Whereas 8.2 kb left lead products were only slightly reduced with Csm3-2A/Tof1-3A (Figure S10C, compare lanes 2 and 5), they were notably less intense for Csm3-5A/Tof1-3A (Figure S10D, lanes 2 and 5), despite comparable levels of the shorter right leading strand being synthesized. Collectively, these data indicate that individual DBMs are dispensable for rapid and efficient DNA replication but that disrupting both motifs compromises the efficiency of leading-strand synthesis.

Rate enhancement by Csm3/Tof1 requires Mrc1 (Yeeles et al., 2017) and Csm3/Tof1 stabilize Mrc1 in the replisome (Bando et al., 2009). We therefore considered that the defects observed with the DBM double mutants might be due to compromised Mrc1 function, for example if they failed to correctly stabilize or position Mrc1 at the replication fork. To test this, we developed an assay to monitor replisome stability using spin columns to isolate DNA-bound proteins from unbound proteins (Figure 6D). Elution of Mcm7, Cdc45, Psf1 (GINS), Ctf4, RPA, Csm3, and Mrc1 was dependent on DNA, ORC (required for MCM loading), and Dpb11 (required for CMG assembly), confirming the detection of replisome-associated proteins (Figure S10E). In the absence of Csm3/Tof1, Mrc1 replisome association was reduced but not eliminated (Figure 6E), consistent with its ability to modestly stimulate fork rate in reactions lacking Csm3/Tof1 (Yeeles et al., 2017). Moreover, this indicates that Mrc1 is stabilized in the replisome by Csm3/Tof1 as is observed *in vivo* (Bando et al., 2009). In contrast, in the reaction containing Csm3-5A/Tof1-3A, Csm3 association was appreciably weaker and levels of Mrc1 were comparable to the experiment lacking Csm3/Tof1 (Figure 6E, compare lanes 4 and 6). Figure S10F shows the Csm3/Tof1 single-DBM mutants associated with the replisome at comparable levels to the wild type, as did Mrc1 in this context. We also observed a defect in the binding of Csm3, Tof1, and Mrc1 to CMG in glycerol gradients with Csm3-5A/Tof1-3A (Figure S10G). The weaker replisome association displayed by Csm3-5A/Tof1-3A indicated the complex may function more distributively during replication and Figure S10H supports this idea: whereas 10 nM Csm3/Tof1 was saturating for replication, DNA synthesis was enhanced across the entire titration range (2.5–80 nM) for Csm3-5A/Tof1-3A. Taken together, these results reveal that the Csm3/Tof1 dsDNA grip stabilizes the entire FPC in the replisome, strongly suggesting the replication defect observed with Csm3-5A/Tof1-3A (Figure 6C) stems from an inability to stabilize Mrc1 (Figure 6E).

### The Csm3/Tof1 dsDNA Grip Is Important for Replication Fork Pausing

**Figure 7:**
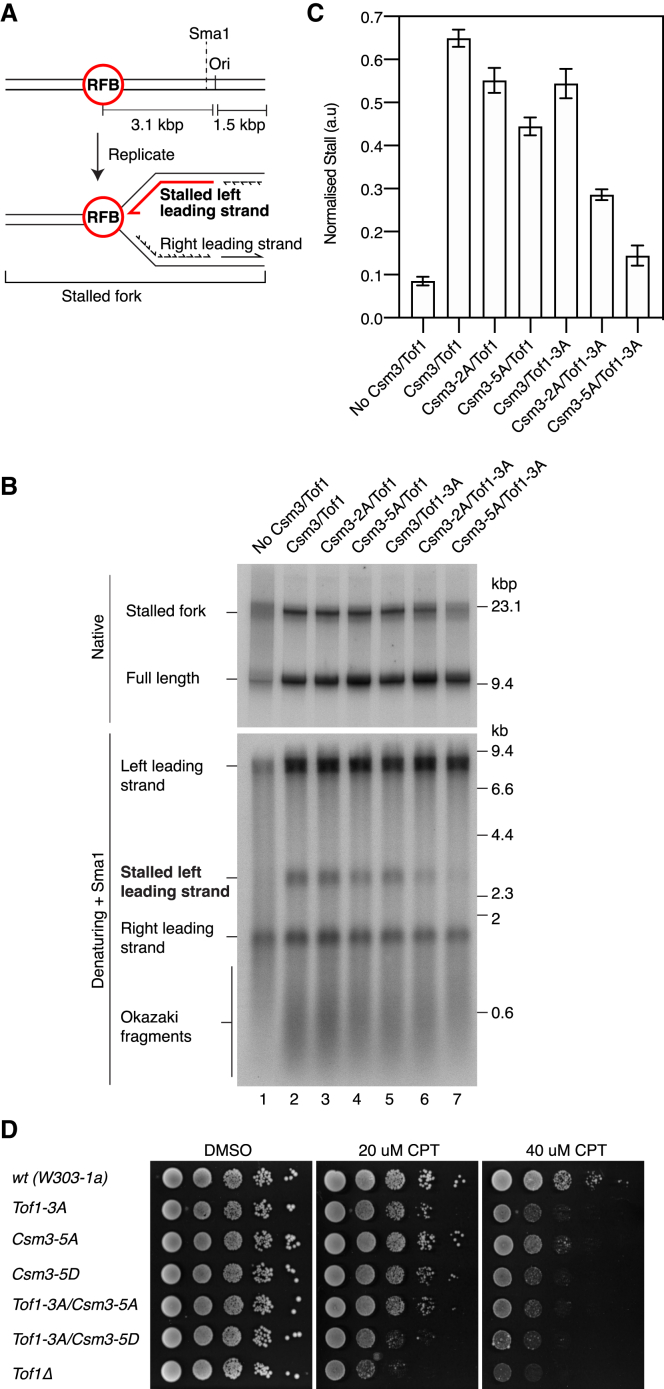
The Csm3/Tof1 dsDNA Grip Is Required for Efficient Fork Pausing (A) Schematic of the template used for replication fork barrier (RFB) experiments and the anticipated products of fork stalling at the RFB. (B) Origin-dependent replication reaction performed for 20 min in the presence of Fob1. The Csm3/Tof1 concentration was increased to 80 nM to increase replication efficiency (Figure S10H). Reaction products were treated with Sma1 prior to denaturing gel electrophoresis to remove heterogeneity in the position of leading-strand initiation (Taylor and Yeeles, 2018). (C) Quantitation of experiments performed as in (B). Error bars represent the SEM from 3 experiments. (D) Spot-dilution assay with Tof1 and Csm3 DBM mutants. 10-fold serial dilutions were plated and grown at 25°C for 3 days.

Csm3/Tof1 is essential for directional fork pausing at replication fork barriers (RFBs) to limit head-on collisions between the replisome and RNA polymerase (Hizume et al., 2018, Mohanty et al., 2006, Takeuchi et al., 2003). The RFB contains multiple binding sites for Fob1 (Kobayashi, 2003), which is required for barrier activity (Kobayashi and Horiuchi, 1996). To assess whether DNA binding by Csm3/Tof1 facilitates fork pausing at the RFB, we performed replication reactions in the presence of Fob1 on a linear template containing the RFB in the non-permissive orientation ∼3 kb left of the origin (Figure 7A). Fork pausing will generate a slowly migrating stalled fork and an ∼3 kb stalled left leading strand that can be visualized in native and denaturing gels, respectively. Figure 7B shows Csm3/Tof1-dependent fork stalling was recapitulated in this system (compare lanes 1 and 2). Similar results were observed in a lower-salt buffer (Yeeles et al., 2017) that supports fully replicated left lead products in the absence of Csm3/Tof1 (Figure S10I, compare lanes 1 and 2). Whereas the responses of Csm3-2A/Tof1 and Csm3/Tof1-3A to the RFB were comparable to the wild type, we observed a 30%–40% reduction in pausing for Csm3-5A/Tof1 and an ∼60% reduction with Csm3-2A/Tof1-3A (Figures 7B, 7C, S10I, and S10J). RFB activity with Csm3-5A/Tof1-3A was reduced almost to background levels. Importantly, although the replisome association of this mutant was compromised (Figures 6E and S10G), it retained the ability to stimulate replication such that the 8.2 kb left lead products in Figure 7B were highly dependent on the mutant protein (compare lanes 1 and 7). The fork pausing defect displayed by Csm3-5A/Tof1-3A therefore likely represents a specific defect in responding to the RFB and is not simply a consequence of the protein being absent from the replisome. These findings demonstrate the Csm3/Tof1 dsDNA grip is required for efficient replication fork pausing at the RFB.

Fork pausing at proteinaceous barriers can also have deleterious consequences, for example when forks stall at covalently trapped topoisomerase I (Topo I) complexes following treatment of cells with camptothecin (CPT). Csm3 and Tof1 are important for cellular tolerance of CPT (Redon et al., 2006), although their mechanism of action is unclear. Given the DBMs are important for pausing at the RFB, we considered they may also be involved in the response to fork stalling during CPT treatment. To test this hypothesis, we generated haploid yeast expressing either Csm3-5A, Tof1-3A, or a Csm3 charge-reversal mutant, Csm3^R46D, K47D, R48D, R49D, K53D^ (Csm3-5D), from the endogenous promoters. *tof1-3A* and *csm3-5D* exhibited mild growth defects at 20 μM CPT, whereas *csm3-5A* grew almost like the wild type (Figure 7D). At 40 μM CPT, both *tof1-3A* and *csm3-5D* displayed clear growth defects and *csm3-5A* showed very mild sensitivity. Combining *csm3-5D* and *tof1-3A* had an additive effect on CPT sensitivity such that cell growth was only marginally better than *tof1*Δ. None of the mutants displayed sensitivity to HU and only *tof1-3A/csm3-5D* showed a very mild sensitivity to methyl methanesulfonate (MMS) (Figure S10K), indicating the observed defects with CPT were not a general response to replication stress. Collectively, these data reveal the Csm3/Tof1 dsDNA grip participates in the maintenance of genome stability following Topo I inhibition by CPT, likely by stabilizing stalled replication forks.

## Discussion

The cryo-EM structures we have determined provide the first near-atomic-resolution views of the CMG helicase bound to accessory proteins at a replication fork, affording the most complete picture of the eukaryotic replisome to date. They reveal the structure of Csm3/Tof1 and its extensive network of interactions with MCM, which redefines the architecture of the front of the replisome by placing the complex ahead of CMG (Figure 1). Additionally, the structures show in detail how ssDNA is engaged by the MCM motor domains in two distinct conformations that likely reflect translocation intermediates, and reveal a mechanism for strand separation involving a separation pin contributed by the NTH of Mcm7.

### Structure and Function of Csm3/Tof1

The crystal structure of the N-terminal domain of human Timeless illustrated this region of the protein adopts a helical-repeat configuration (Holzer et al., 2017). Our work develops this finding by showing the helical repeats extend for approximately two-thirds of Tof1 and are capped at the C-terminal end by Csm3. The primary interface between the two subunits comprises a short hydrophobic helix from Tof1 that is encased by Csm3, which likely explains why Csm3 is unstable in the absence of Tof1 (Bando et al., 2009) and cellular levels of Timeless and Tipin are interdependent (Chou and Elledge, 2006). Our structure also highlights the critical importance of two large loops (Ω-loop and MCM-plugin) inserted between helical repeats that serve to functionalize the Tof1 α solenoid by anchoring it to CMG and the replication fork. We anticipate human Tipin/Timeless will be anchored to CMG in a similar manner as many of the structural features we identify involved in CMG binding are highly conserved. We note, however, that the loop in Timeless equivalent to the Ω-loop is considerably shorter than in *S. cerevisiae* and therefore its interactions with both MCM and dsDNA may differ.

In addition to binding Mrc1, Tof1 interacts with Topo I via its C-terminal region that is not resolved in our structure (Park and Sternglanz, 1999). The advanced positioning of Csm3/Tof1 in the replisome should facilitate Topo I recruitment ahead of the fork, which may serve to limit excessive fork rotation (Schalbetter et al., 2015). Similarly, Timeless binds directly to the helicase DDX11 that is involved in sister chromatid cohesion (Cortone et al., 2018) and therefore DDX11 is most likely recruited to the front of the replisome to perform this function.

The conformations of Csm3/Tof1 and the dsDNA grip we observe are likely to be engaged during unperturbed replisome progression because mutation of both DBMs compromises replication in the absence of exogenous DNA damage (Figure 6C). Moreover, our work demonstrates the grip plays a key role in stabilizing the entire FPC in the replisome (Figures 6E and S10G) and is especially important for efficient fork pausing at the RFB (Figure 7B). Although the mechanism of RFB recognition is currently unknown, we speculate the dsDNA grip might function to stabilize the replisome on the template once Fob1 is encountered by the fork. Alternatively, it might be required to correctly position Csm3/Tof1 at the front of the replisome to recognize Fob1, perhaps via a direct protein-protein interaction. It was recently reported that Tof1 recruits Topo I to the replisome for efficient fork pausing at the RFB *in vivo* (Shyian et al., 2020). Our experiments were performed on linear templates in the absence of topoisomerases, indicating that Topo I is not absolutely required for Csm3/Tof1-dependent RFB recognition. Gripping dsDNA could also enable Csm3/Tof1 to detect structural perturbations in the DNA template or protein roadblocks in advance of CMG, which might be important for its fork stabilization functions. This hypothesis is consistent with the involvement of the dsDNA grip in the cellular tolerance of CPT (Figure 7D).

The normal replication rates displayed by the single-DBM mutants (Figure 6C), the dependence on Mrc1 for Csm3/Tof1-dependent rate enhancement (Yeeles et al., 2017), and the reduced association of Mrc1 in experiments using Csm3-5A/Tof1-3A (Figure 6E) indicate that Csm3/Tof1 promote rate enhancement indirectly, likely by stabilizing and/or positioning Mrc1 in the replisome. It was reported that Timeless is displaced from replisomes in human cells to slow replication in response to redox changes (Somyajit et al., 2017) and our findings now indicate this displacement could be mediated by disrupting the dsDNA grip.

### Position of Mrc1 in the Replisome

Although Mrc1 was not resolved in the cryo-EM structure, XL-MS data strongly suggest it stretches from the front of the replisome to the rear (Figure 2), affording a mechanism for coordinating events ahead of the fork—perhaps monitored by Csm3/Tof1—with leading-strand polymerization. Moreover, the positioning of Mrc1 across one side of CMG spanning N and C tiers could enable it to directly modulate helicase activity to control fork rate and maintain the coupling of DNA synthesis to CMG template unwinding when leading-strand polymerization is compromised (Katou et al., 2003). Several of the Mrc1 cross-linking sites we identify on CMG are in close proximity to cross-linking sites previously identified for the essential firing factor Mcm10 (Mayle et al., 2019), indicating the two proteins might compete for CMG binding. It will be interesting to discover whether the two proteins can bind simultaneously to CMG during replisome progression. Although we cannot exclude the possibility that multiple copies of Mrc1 associate with the replisome, we consider it most likely that a single protein was present in our complex based on the apparent 1:1 stoichiometry observed during glycerol gradients (Figure 1A).

It is currently unclear how Mrc1 and Claspin stimulate fork rate in yeast and human cells. The N-terminal half of Mrc1 interacts with the flexibly linked catalytic domain of Pol ε (Lou et al., 2008) and several amino acids from this region cross-linked to Mcm6 and Mcm2 in close proximity to where the unwound leading-strand template will emerge from CMG (Figure 2A). It is therefore possible Mrc1 might accelerate forks by tethering the flexible catalytic domain of Pol ε (Zhou et al., 2017) to this region of CMG to facilitate optimal helicase-polymerase coupling. The structure of a replisome containing both Mrc1 and Pol ε should help elucidate this mechanism.

### CMG-DNA Interactions and a Mechanism of Strand Separation

The structures we have determined offer near-atomic-resolution views of eukaryotic CMG-DNA interactions (Figure 3). Notably, conformation 2 represents the first example of ssDNA engagement across all six MCM subunits of CMG, as well as the first time a conformation with DNA bound across Mcm47 has been observed outside *Drosophila*. Although previous work highlighted the involvement of H2I and PS1 loops in CMG-ssDNA engagement (Abid Ali et al., 2016, Eickhoff et al., 2019, Georgescu et al., 2017, Goswami et al., 2018), description of the residues involved was limited to the PS1 lysine and H2I basic residues (Yuan et al., 2020). The improved resolution of our structures has enabled a complete ssDNA-binding motif to be described for eukaryotic CMG, demonstrating conservation of the mode of ssDNA engagement across eukaryotic and archaeal MCMs (Meagher et al., 2019). This has revealed a set of phosphate contacts displaying repetition across all six MCM subunits in the heterohexamer, presumably to ensure ssDNA can be engaged by all subunits at different points during a rotary translocation cycle (Eickhoff et al., 2019). In contrast, the sugar and base contacts formed by the H2I loops display greater diversity between MCM subunits; the contribution of these contacts to translocation in eukaryotic CMG remains unknown and roles in DNA melting during helicase activation have been hypothesized (Meagher et al., 2019).

The location of the fork junction in our structures is in good agreement with several structures of CMG obtained in the presence of ATP (Georgescu et al., 2017, Yuan et al., 2020), strongly suggesting this represents the point of template unwinding in the replisome. This conclusion is further supported by our identification of a putative strand-separation pin, the Mcm7 NTH, which abuts the final base pair of the duplex in both conformations 1 and 2. A recent paper, published while this manuscript was in revision, proposed a dam-and-diversion model for template unwinding that does not utilize a separation pin (Yuan et al., 2020). Although our work supports a model whereby the NTHs of Mcm6 and Mcm4 guide the incoming dsDNA along a defined path toward the helicase channel, consistent with the dam-and-diversion model, the improved resolution of the Mcm7 NTH in our structures shows it remodeling to position a conserved phenylalanine against the final base pair, which supports its role as a separation pin. The stable positioning of dsDNA ahead of CMG (Eickhoff et al., 2019) and utilization of a defined separation pin should help ensure the correct positioning of unwound template strands within the replisome for downstream processes. Moreover, if the C tier motor domains were to disengage from ssDNA, the extensive contacts around the fork junction, together with the Csm3/Tof1 dsDNA grip, might function to prevent helicase backtracking.

The cryo-EM structure of the FPC bound to CMG at a replication fork represents a significant advance in our understanding of eukaryotic replisome structure and mechanism. Yet, given its complexity, subunit dynamics, and sophisticated regulation, much work remains to be done. The work presented here provides an ideal platform to build ever-more complex replisome assemblies for analysis by cryo-EM. This will be crucial to facilitate a complete mechanistic description of this remarkable molecular machine.

## STAR★Methods

### Key Resources Table

**Table:** 

REAGENT or RESOURCE	SOURCE	IDENTIFIER
**Antibodies**		

α-Csm3	Maric et al., 2014	N/A
α-Ctf4	Maric et al., 2014	N/A
α-FLAG	Sigma	Cat# A8592; RRID:AB_439702
α-Mcm7	Maric et al., 2014	N/A
α-Mrc1	Mukherjee and Labib, 2019	N/A
α-RFA	Agrisera	Cat# AS07 214; RRID:AB_1031803
α-Psf1	Maric et al., 2014	N/A

**Bacterial and Virus Strains**		

5-alpha Competent *E. coli* (High Efficiency)	New England Biolabs	Cat# C2987H
*Escherichia coli*: Rosetta 2(DE3) strain: F^-^*ompT hsdS*_B_(r_B_^-^ m_B_^-^) *gal dcm* (DE3) pRARE2 (Cam^R^)	Novagen / Merck Millipore	Cat# 71400

**Chemicals, Peptides, and Recombinant Proteins**		

3X FLAG peptide	Sigma	Cat# F4799
Adenosine 5′-(β,γ-imido)triphosphate lithium salt hydrate (AMP-PNP)	Sigma	Cat# A2647
dNTP set	Invitrogen	Cat# 10297018
NTP set	Invitrogen	Cat# R0481
[alpha-P32]dCTP	Hatmann analytic	Cat# SRP-205
Anti-FLAG M2 affinity gel	Sigma	Cat# A2220
Bio-Gel HT (Hydrated) Hydroxyapatite	Bio-Rad	Cat# 130-0150
Calmodulin-Sepharose 4B	GE Healthcare	Cat# 17-0529-01
Camptothecin, *Camptotheca acuminata*	Merck	Cat# 208925
cOmplete, EDTA-free	Roche	Cat# 5056489001
Disuccinimidyl dibutyric urea (DSBU)	ThermoScientific	Cat# A35459
Glutaraldehyde	Sigma	Cat# G5882
Nonidet P-40 substitute (NP-40-S)	Roche	Cat# 11754599001
Glutathione Sepharose 4B	GE Healthcare	Cat# 17-0756-01
HiTrap Blue HP	GE Healthcare	Cat# 17-0412-01
HiTrap DEAE Fast Flow	GE Healthcare	Cat# 17-5055-01
HiTrap Heparin HP	GE Healthcare	Cat# 17-0406-01
HiTrap SP HP	GE Healthcare	Cat# 29-0513-24
IgG Sepharose Fast Flow	GE Healthcare	Cat# 17-0969-01
Micro SpinColumn, C18 column	Harvard Apparatus	Cat# 74-4607
MonoQ PC 1.6/5	GE Healthcare	Cat# 17-0671-01
MonoQ 5/50 GL	GE Healthcare	Cat# 17-5166-01
MonoS 5/50 GL	GE Healthcare	Cat# 17-5168-01
Ni-NTA Agarose	QIAGEN	Cat# 30210
Phosbind acrylamide	APExBIO	Cat# F4002
Sephacryl™ S400 High Resolution	GE Healthcare	Cat# GE27-5330-02
Suberic acid bis(3-sulfo-N-hydroxysuccinimide ester) sodium salt (BS^3^)	Sigma	Cat# S5799
Superdex 200 Increase 10/300 GL	GE Healthcare	Cat# 28-9909-44
Superose™ 6 Increase 10/300 GL	GE Healthcare	Cat# 29-0915-96
TWEEN® 20 (used for buffer exchange prior to cryo-EM grid preparation)	Sigma	Cat# P8341
Microspin G-50 columns	GE Healthcare	Cat# GE27-5330-02

**Recombinant Proteins (see also**Table S5)		

Cdt1-Mcm2-7	Coster et al., 2014	N/A
ORC	Frigola et al., 2013	N/A
Cdc6	Frigola et al., 2013	N/A
DDK	On et al., 2014	N/A
Sld3/7	Yeeles et al., 2015	N/A
Cdc45	Yeeles et al., 2015	N/A
Dpb11	Yeeles et al., 2015	N/A
Sld2	Yeeles et al., 2015	N/A
GINS	Yeeles et al., 2015	N/A
Pol ε	Yeeles et al., 2015	N/A
S-CDK	Yeeles et al., 2015	N/A
Mcm10	Yeeles et al., 2015	N/A
Pol α	Yeeles et al., 2015	N/A
Ctf4	Yeeles et al., 2015	N/A
RPA	This study	N/A
Mrc1	This study	N/A
Csm3/Tof1	This study	N/A
RFC	Yeeles et al., 2017	N/A
PCNA	Yeeles et al., 2017	N/A
Pol δ	Yeeles et al., 2017	N/A
Fob1	This study	N/A
Csm3-2A/Tof1	This study	N/A
Csm3-5A/Tof1	This study	N/A
Csm3/Tof1-3A	This study	N/A
Csm3-2A/Tof1-3A	This study	N/A
Csm3-5A/Tof1-3A	This study	N/A
Lambda phosphatase	He Laboratory	N/A
Bovine Serum Albumin	Invitrogen	Cat# AM2616

**Deposited Data**		

Co-ordinate file for conformation 1 (CMG-Csm3-Tof1-Ctf4_3_-fork DNA, reconstituted sample)	This study	PDB: 6SKL
Co-ordinate file for conformation 2 (MCM C-Tier-ssDNA, reconstituted sample)	This study	PDB: 6SKO
Map of conformation 1 (CMG-Csm3-Tof1-Ctf4-fork DNA, reconstituted sample)	This study	EMDB: EMD-10227
Map of conformation 2 (multi-body refinement of MCM[C-tier], reconstituted sample)	This study	EMDB: EMD-10230
Map used in building Csm3-Tof1 atomic model (multi-body refinement of Csm3-Tof1[body]-Mcm467[NTier], reconstituted sample)	This study	EMDB: EMD-10507
Map used in building Csm3-Tof1 atomic model (multi-body refinement of Tof1[head]-Mcm235[NTier], reconstituted sample)	This study	EMDB: EMD-10508
Map of conformation 1 (multi-body refinement of Cdc45-GINS-Ctf4, reconstituted sample)	This study	EMDB: EMD-10509
Map of conformation 1 (multi-body refinement of Mcm2356, reconstituted sample)	This study	EMDB: EMD-10510
Map of conformation 1 (multi-body refinement of Mcm47, reconstituted sample)	This study	EMDB: EMD-10511
Map of conformation 2 (multi-body refinement of Mcm25 + Mcm6 CTD, 5 AMP-PNP bound, reconstituted sample)	This study	EMDB: EMD-10730

**Experimental Models: Organisms/Strains**		

*S. cerevisiae* strains are detailed in Table S4	N/A	N/A

**Oligonucleotides**		

Fork leading strand: 5′-(Cy3)TAGAGTAGGAAGTGA(Biotinylated-dT)GGTAA GTGATTAGAGAATTGGAGAGTGTG(T)_34_T^∗^T^∗^T^∗^T^∗^T^∗^T (^∗^-phosphorothioate)	Integrated DNA Technologies (IDT)	N/A
Fork lagging strand: GGCAGGCAGGCAGGCACACACTCTCC AATTCTCT AATCACTTACCA(Biotinylated-dT)CACTT CCTACTCTA	Integrated DNA Technologies (IDT)	N/A

**Recombinant DNA (See also**Table S3**)**		

vVA20 (replication/recruitment assay template)	Aria and Yeeles, 2018	N/A
ZN5 (replication assay)	Taylor and Yeeles, 2018	N/A
pAM3 (Cdc6 purification)	Frigola et al., 2013	N/A
pJFDJ5 (GINS purification)	Yeeles et al., 2015	N/A
pET28a-Mcm10 (Mcm10 purification)	Yeeles et al., 2015	N/A
vJY19 (PCNA purification)	Yeeles et al., 2017	N/A
vJY23 (Psf1, Sld5)	This study	N/A
vJY24 (Psf2, Psf3)	This study	N/A
vJY25 (Fob1)	This study	N/A
vJY30 (RFB template)	This study	N/A
vJY71 (Cdc45, Ctf4)	This study	N/A
vJY72 (Csm3, Tof1)	This study	N/A
vJY74 (Mrc1)	This study	N/A
vJY111 (Rfa1)	This study	N/A
vJY113 (Csm3^R49A, K53A^-Tof1)	This study	N/A
vJY114 (Csm3-Tof1^K400A, R401A, K404A^)	This study	N/A
vJY115 (Csm3^R49A, K53A^-Tof1^K400A, R401A, K404A^)	This study	N/A
vJY116 (Csm3^K47A, R48A, R49A, Q51A, K53A^-Tof1)	This study	N/A
vJY117 (Csm3^K47A, R48A, R49A, Q51A, K53A^-Tof1^K400A, R401A, K404A^)	This study	N/A
vVA30 (Parent vector for Tof1 mutagenesis)	This study	N/A
vVA31 (Construction of Tof1-3A strains)	This study	N/A
vVA32 (Parent vector for Csm3 mutagenesis)	This study	N/A
vJY136 (Construction of Csm3-5D strains)	This study	N/A
vJY137 (Construction of Csm3-5A strains)	This study	N/A

**Software and Algorithms**		

CCP-EM (dev1.2.0)	CCP-EM	https://www.ccpem.ac.uk/
Chimera (v1.13)	UCSF Resource for Biocomputing, Visualization, and Informatics	https://www.cgl.ucsf.edu/chimera/
ChimeraX (v0.91)	UCSF Resource for Biocomputing, Visualization, and Informatics	https://www.cgl.ucsf.edu/chimerax/
Coot (v0.9-pre)	Paul Emsley (Medical Research Council Laboratory of Molecular Biology)	https://www2.mrc-lmb.cam.ac.uk/personal/pemsley/coot/
EMAN (v1.9)	Baylor College of Medicine	https://cryoem.bcm.edu/downloads/view_eman1_versions
EPU (v1.9.1 & AutoCTF)	ThermoFisher Scientific (FEI)	https://www.fei.com/software/epu-automated-single-particles-software-for-life-sciences/
ESPript (v3.0.7)	Patrice Gouet (Lyon University); Xavier Robert (Centre national de la recherche scientifique)	http://espript.ibcp.fr/ESPript/ESPript/
FIJI (v1.0)	National Institute of Health	https://imagej.net/Fiji/Downloads
Gautomatch (v0.53)	Kai Zhang (Medical Research Council Laboratory of Molecular Biology)	https://www.mrc-lmb.cam.ac.uk/kzhang/Gautomatch/
Gctf (v0.50)	Kai Zhang (Medical Research Council Laboratory of Molecular Biology)	https://www.mrc-lmb.cam.ac.uk/kzhang/Gctf/
ImageJ (v1.50i)	National Institute of Health	https://imagej.nih.gov/ij/
ISOLDE (v1.0b4)	Tristan Croll (Cambridge Institute for Medical Research)	https://isolde.cimr.cam.ac.uk/
Jalview (2.12.2b2)	Barton Group, University of Dundee	https://www.jalview.org/
MacPyMOL (v1.8.6.0)	Schrödinger	https://pymol.org/2/
MeroX	Michael Götze (ETH Zürich Institute of Molecular Systems Biology)	http://www.stavrox.com/
MolProbity	Duke Univeristy	http://molprobity.biochem.duke.edu/
MotionCor2 (v1)	University of California San Francisco (UCSF) EM Core	https://emcore.ucsf.edu/ucsf-motioncor2
MSConvert	ProteoWizard	http://proteowizard.sourceforge.net/index.html
MUSCLE	European Molecular Biology Laboratory -European Bioinformatics Institute (EMBL-EBI)	https://www.ebi.ac.uk/Tools/msa/muscle/
PDBePISA (v1.48)	European Molecular Biology Laboratory -European Bioinformatics Institute (EMBL-EBI)	https://www.ebi.ac.uk/pdbe/pisa/
Phenix (v1.16-3549)	Cambridge University; Duke University; Lawrence Berkeley National Laboratory; Los Alamos National Laboratory	https://www.phenix-online.org/
Photoshop CC 2018	Adobe	https://www.adobe.com/uk/products/photoshop.html
Phyre2	Structural Bioinformatics Group, Imperial College London	http://www.sbg.bio.ic.ac.uk/∼phyre2/
Prism (v8.0.0)	GraphPad	https://www.graphpad.com/scientific-software/prism/
ProSMART (v0.856)	Garib Murshudov (Medical Research Council Laboratory of Molecular Biology)	https://www2.mrc-lmb.cam.ac.uk/groups/murshudov/content/prosmart/documentation.html
Refmac (v5.8.0238)	Garib Murshudov (Medical Research Council Laboratory of Molecular Biology)	https://www2.mrc-lmb.cam.ac.uk/groups/murshudov/content/refmac/refmac.html
RELION (v2.1 & v3.0.6)	Sjors Scheres (Medical Research Council Laboratory of Molecular Biology)	https://www3.mrc-lmb.cam.ac.uk/relion/
Xcalibur™	ThermoFisher Scientific	https://www.thermofisher.com/order/catalog/product/OPTON-30965#/OPTON-30965
Xlink Analyzer (v1.1.4 dev29012020)	European Molecular Biology Laboratory (EMBL) - Hamburg	https://www.embl-hamburg.de/XlinkAnalyzer/XlinkAnalyzer.html
XMIPP	Centro Nacional de Biotecnologia (CNB) Instruct Image Processing Centre (I2PC)	http://xmipp.i2pc.es/

**Other**		

QUANTIFOIL Copper 400 mesh R2/2 holey carbon TEM grids	Electron Microscopy Sciences	Cat# Q450CR2

### Resource Availability

#### Lead Contact

Further information and requests for resources and reagents should be directed to and will be fulfilled by the Lead Contact, Joseph Yeeles (jyeeles@mrc-lmb.cam.ac.uk).

#### Materials Availability

Unique and stable reagents generated in this study are available upon request.

#### Data and Code Availability

Cryo-EM density maps of the reconstituted complex used in model building have been deposited in the Electron Microscopy Data Bank (EMDB) under the following accession numbers: for conformation 1, EMD-10227 (full complex), EMD-10507 (Csm3-Tof1^Body^-Mcm467^N-tier^), EMD-10508 (Tof1^Head^-Mcm235^N-tier^), EMD-10509 (Cdc45-GINS-Ctf4_3_), EMD-10510 (Mcm2356), EMD-10511 (Mcm47); for conformation 2, EMD-10230 (MCM^C-tier^), EMD-10730 (Mcm25-Mcm6^C-tier^). Atomic coordinates have been deposited in the Protein Data Bank (PDB) with the accession numbers PDB: 6SKL (conformation 1) and PDB: 6SKO (conformation 2, MCM^C-tier [5 AMP-PNP]^).

### Experimental Model and Subject Details

Proteins were purified from *Saccharomyces cerevisiae* strains (genotype: MATa ade2-1 ura3-1 his3-11,15 trp1-1 leu2-3,112 can1-100 bar1::Hyg pep4::KanMX) containing integrated expression constructs; or from *Escherichia coli* RosettaTM 2(DE3) cells (Novagen) (genotype: F– ompT hsdSB(rB– mB–) gal dcm (DE3) pRARE2 (CamR)) transformed with plasmids for protein overexpression (see Key Resources Table and Tables S3–S5 for details. Yeast strains for harboring Csm3 and Tof1 mutations were derived from W303-1a (genotype: MATa ade2-1 ura3-1 his3-11,15 trp1-1 leu2-3,112 can1-100). Plasmids details are reported in the Key Resources Table and Table S3.

### Method Details

#### Yeast strains

Vectors and strains were constructed using standard molecular biology techniques (see Tables S3 and S4 for details). All genes for protein expression were codon optimized as described (Yeeles et al., 2015). All mutant haploid yeast strains were isolated by tetrad dissection of heterozygous diploid strains. Coding sequences for all genes were verified by sequencing, as were the coding regions of mutant alleles of Csm3 and Tof1 following PCR amplification from genomic DNA.

#### Protein purification

Cdt1⋅Mcm2-7, ORC, Cdc6, DDK, Sld3/7, Sld2, Cdc45, S-CDK, Dpb11, GINS, Pol ε, Mcm10, RPA, RFC, PCNA, Pol α, Pol δ, Csm3/Tof1 and Ctf4 were purified as previously described (Taylor and Yeeles, 2018, Yeeles et al., 2015, Yeeles et al., 2017). An overview of the purification strategy for each protein is provided in Table S5.

##### RPA purification

Untagged *S. cerevisiae* RPA was purified from a 10 L culture of yJY106. Cells were grown at 30°C to 5 x10^7^ cells per ml in YEP (1.1% yeast extract, 2.2% bactopeptone, 55 mg/L adenine hemisulphate) + 2% w/v raffinose before induction by addition of galactose to 2% w/v final concentration from a 20% w/v stock. Cell growth was continued for 3 hours at 30°C before cells were harvested by centrifugation, washed in 100 mL 25 mM Tric-HCL pH 7.2, 10% glycerol, 500 mM NaCl, 1 mM Tris(2-carboxyethyl)phosphine hydrochloride (TCEP) (buffer R + 500 mM NaCl) and resuspended in buffer R. Cell paste was frozen in liquid nitrogen and cells were lysed using a pestle and mortar filled with liquid nitrogen. The lysate was cleared by centrifugation (235,000 g, 4°C, 1 hour) and nucleic acid precipitated by addition of polyethyleneimine to 0.025% from a 1% stock followed by gentle stirring at 4°C for 10 min. Precipitate was cleared by centrifugation (18,000 g, 4°C, 15 min) and solid ammonium sulfate was added slowly to 40% saturation. Following gentle stirring at 4°C for 10 min, precipitated protein was collected by centrifugation (18,000 g, 4°C, 20 min) and the precipitate resuspended in buffer R + 500 mM NaCl. The conductivity of the protein sample was adjusted to be equivalent to buffer R + 500 mM NaCl by dilution with buffer R before application to a HiTrap Blue column equilibrated in buffer R + 500 mM NaCl. All subsequent purification steps were performed as described in (Devbhandari et al., 2017).

##### Mrc1 purification

Mrc1 was purified as previously described (Yeeles et al., 2017) but with the following modifications. The growth temperature during protein expression was reduced from 30°C to 20°C. All subsequent steps were performed at 4°C. Lysed cell powder from a 10-15 L culture was resuspended in buffer M (50 mM Tris-HCl pH 8, 10% glycerol, 0.005% TWEEN 20, 0.5 mM TCEP, 400 mM NaCl) + protease inhibitors (cOmplete, EDTA-free (Roche), one tablet per 50 mL buffer). Insoluble material was cleared by centrifugation (235,000 g, 4°C, 1 hour) and 2-4 mL FLAG M2 Affinity gel (Sigma) was added to the supernatant. The sample was incubated for 100 min before the resin was collected in 20 mL columns (< 2 mL bed volume per column) and was washed with 75 mL buffer M. Columns were washed with 12.5 mL buffer M + 5 mM Mg(OAc)_2_ + 0.5 mM ATP, followed by 25 mL buffer M. Mrc1 was eluted in 1 column volume (CV) buffer M + 0.2 mg/ml 3x FLAG peptide and 2 CV buffer M + 0.1 mg/ml 3x FLAG peptide. The eluate was concentrated to ∼800 μL in an Amicon Ultra-15 30,000 NMWL concentrator and applied to a Superose 6 10/300 column (GE healthcare) equilibrated in 25 mM Tris-HCl pH 7.2, 10% glycerol, 0.005% TWEEN 20, 1 mM EDTA, 0.5 mM TCEP, 150 mM NaCl. Peak fractions were pooled, frozen in liquid nitrogen and stored at −80°C.

##### Csm3/Tof1 purification

Csm3/Tof1 was purified as previously described (Yeeles et al., 2017) but with the following modifications. After elution from Calmodulin Sepharose 4B (GE healthcare) by TEV cleavage the protein was applied to a 1 mL MonoQ column equilibrated in 25 mM Tris-HCl pH 7.2, 1 mM EDTA, 10% glycerol, 0.02% NP-40-S, 1 mM DTT, 200 mM NaCl. Protein was eluted with a 30 column volume gradient from 200 mM – 1M NaCl. Peak fractions were pooled, concentrated to ∼500 μL in an Amicon Ultra-15 30,000 NMWL concentrator and applied to a Superdex 200 Increase 10/300 gel filtration column equilibrated in 25 mM Tris-HCl pH 7.2, 1 mM EDTA, 10% glycerol, 0.02% NP-40-S, 1 mM DTT, 150 mM NaCl. Peak fractions were pooled, frozen in liquid nitrogen and stored at −80°C.

##### CMG purification

Diploid yeast (yJY37) (15-30 L) were grown at 30°C to 5 x10^7^ cells per ml in YEP + 2% w/v raffinose before induction by addition of galactose to 2% w/v final concentration from a 20% w/v stock. Cell growth was continued for 3 hours at 30°C before cells were harvested by centrifugation, washed in 150 mL buffer C (40 mM HEPES-NaOH pH 7.5, 10% glycerol, 0.005% TWEEN 20, 0.5 mM TCEP, 150 mM NaOAc) and resuspended in a minimal volume of buffer C + protease inhibitors (cOmplete, EDTA-free (Roche), one tablet per 50 mL buffer). Cell paste was frozen in liquid nitrogen and cells were lysed using a pestle and mortar filled with liquid nitrogen. Lysed cell powder (typically from a 15 L culture) was resuspended in buffer C + protease inhibitors and insoluble material removed by centrifugation (235,000 g, 4°C, 1 hour). FLAG M2 Affinity gel (8 ml) was added to the lysate and incubated for 90 min at 4°C. Resin was collected in 20 mL columns (< 2 mL bed volume per column) and washed with 80 mL buffer C per column. Columns were then washed with 10 mL buffer C + 5 mM Mg(OAc)_2_ + 0.5 mM ATP followed by 25 mL buffer C. Proteins were eluted with 1 CV buffer C + 2mM CaCl_2_ + 0.2 mg/ml 3x FLAG peptide then 2 CV buffer C + 2mM CaCl_2_ + 0.1 mg/ml 3x FLAG peptide. Calmodulin Sepharose 4B (GE healthcare) (1 ml) was immediately added to the eluate, which was incubated for 30 min before the resin was collected in a 20 mL column. The flow-through was reapplied to the column twice before washing the column with 25 CV buffer C + 2mM CaCl_2_. CMG was eluted in 8 CV of buffer C + 2 mM EDTA + 2 mM EGTA. Eluate was applied to a MonoQ PC 1.6/5 (GE healthcare) equilibrated in 25 mM Tris-HCl pH 7.2, 10% glycerol, 0.005% TWEEN 20, 0.5 mM TCEP, 150 mM KCl. CMG was eluted with a 20 CV gradient from 150-1000 mM KCl and peak fractions were dialysed overnight against 500 mL 25 mM HEPES-KOH pH 7.6, 40 mM KOAc, 40 mM K-glutamate, 2 mM Mg(OAc)_2_, 0.25 mM EDTA, 0.5 mM TCEP, 20% glycerol. Protein was frozen in liquid nitrogen and stored at −80°C.

##### Fob1 purification

yJY39 (10 L) were grown at 30°C to 4.5 x10^7^ cells per ml in YEP + 2% w/v raffinose before induction by addition of galactose to 2% w/v final concentration from a 20% w/v stock. Cell growth was continued for 3 hours at 30°C before cells were harvested by centrifugation, washed in 150 mL buffer F (25 mM Tris-HCl pH 7.2, 1 mM EDTA, 10% glycerol, 0.02% NP-40-S, 0.5 mM DTT) + 400 mM NaCl and resuspended in a minimal volume of buffer F + 400 mM NaCl + protease inhibitors (cOmplete, EDTA-free (Roche), one tablet per 25 mL buffer). Cell paste was frozen in liquid nitrogen and cells were lysed using a pestle and mortar filled with liquid nitrogen. Lysed cell powder was resuspended in buffer F + 400 mM NaCl + protease inhibitors and insoluble material removed by centrifugation (235,000 g, 4°C, 1 hour). FLAG M2 Affinity gel (2.5 ml) was added to the lysate and incubated for 3 hours at 4°C. Resin was collected in a 20 mL column and washed with 80 mL buffer F + 400 mM NaCl followed by 20 mL buffer F + 200 mM NaCl. Fob1 was eluted in 8 mL buffer F + 200 mM NaCl + 0.2 mg/ml 3x FLAG peptide. The eluate was diluted in buffer F to the equivalent of 150 mM NaCl and was applied to a 1 mL MonoQ column equilibrated in buffer F + 150 mM NaCl. Protein was eluted with a 25 CV gradient from 150-1000 mM NaCl in buffer F. Peak fractions were pooled and dialysed against buffer F + 150 mM NaCl for 3 hours prior to freezing in liquid nitrogen and storage at −80°C.

#### Preparation of fork DNA for cryo-EM sample preparation

Fork DNA was annealed by mixing equal volumes of Fork-Lead and Fork-Lag oligos (Integrated DNA Technologies) and allowing to cool gradually from 75°C to room temperature. The Fork-Lead and Fork-Lag stock solutions were made at 53 μM each in 25 mM HEPES-NaOH, pH 7.5, 150 mM NaOAc, 0.5 mM TCEP, 2 mM Mg(OAc)_2_. The sequence of each oligo was a modified version of the fork used in prior publication (Georgescu et al., 2017); Fork-Lead was 5′-(Cy3)TAGAGTAGGAAGTGA(Biotinylated-dT)GGTAAGTG ATTAGAGAATTGGAGAGTGTG(T)_34_T^∗^T^∗^T^∗^T^∗^T^∗^T, where ^∗^ denotes phosphorothioate backbone linkages. Fork-Lag was 5′-GGCAGGCAGGCAGGCACACACTCTCCAATTCTCTAATCACTTACCA(Biotinylated-dT)CACTTCCTACTCTA.

#### Glycerol 10%–30% gradient preparation

For co-expression experiments, Buffer A (40 mM HEPES-NaOH, pH 7.5, 150 mM NaOAc, 0.5 mM TCEP, 10% v/v glycerol) was layered on top an equal volume of freshly prepared Buffer B (Buffer A, except 30% v/v glycerol + 0.16% glutaraldehyde [Sigma]) in a 14 mL SW40-Ti tube (Beckman) and gradients made using a gradient-making station (Biocomp Instruments, Ltd.) before cooling on ice.

For *in vitro* reconstitution experiments, 500 μM AMP-PNP and 3 mM Mg(OAc)_2_ were added to Buffers A and B. Buffer B was further supplemented with a second cross-linking agent, 2 mM bis(sulfosuccinimidyl)suberate (BS^3^, ThermoFisher Scientific). These were layered in equal volumes in a 2.2 mL TLS-55 tube (Beranek Laborgerate) and gradients prepared using a gradient-making station (Biocomp Instruments, Ltd.) before cooling on ice.

#### *In vitro* reconstitution of CMG-Csm3/Tof1-Mrc1-Ctf4-DNA complexes for cryo-EM

Components were sequentially mixed with CMG while on ice as follows to yield a final reaction volume of 65 μL containing 0.5 μM CMG with a 1.5 molar excess of all other components, maintaining 500 μM AMP-PNP and 3 mM Mg(OAc)_2_ throughout. First, the fork DNA was added to CMG and incubated for 1 h. Subsequently, Csm3/Tof1 and Ctf4 were pre-mixed and added to the CMG:DNA reaction mixture. After 10 min incubation, Mrc1 was added for a further 45 min.

Before loading onto the glycerol gradient (prepared as described above), the reaction volume was diluted 2.5-fold using buffer D (25 mM HEPES-NaOH, pH 7.5, 150 mM NaOAc, 0.5 mM TCEP, 500 μM AMP-PNP, 3 mM Mg(OAc)_2_). The sample was separated by centrifugation (Beckman TLS-55 rotor, 200,000*g*, 4°C, 2 h) and 100 μL fractions manually collected. The fraction containing the complex was identified by silver-stained SDS-PAGE. Relevant fractions were pooled (total ∼190 μL) and buffer exchanged with cryo-EM buffer (buffer D except 100 μM AMP-PNP + 0.005% v/v TWEEN 20 [Sigma, Cat#P8341]) during six rounds of ultrafiltration in 0.5 mL 30K MWCO centrifugal filters (Amicon) using a bench-top centrifuge (21,000*g,* 4°C, 1 min/round). Sample was concentrated to ∼25 μL and immediately used for cryo-EM grid preparation.

#### Co-expression and purification of CMG-Csm3/Tof1-Mrc1-Ctf4 complexes for cryo-EM

Cultures of yJY74 (see Tables S4 and S5 for details) were grown in YEP with 2% w/v raffinose (15 L) at 30°C, to a density of ∼6 × 10^7^ cells/mL before inducing overexpression by addition of 2% w/v galactose for 3 h under the same conditions. Cells were harvested by centrifugation (3,000*g*, 8 min, 4°C), washed and resuspended with Lysis buffer (40 mM HEPES-NaOH, pH 7.5, 150 mM NaOAc, 10% glycerol, 0.005% v/v TWEEN 20, 0.5 mM TCEP, protease-inhibitors (cOmplete, EDTA-free (Roche), one tablet per 25 mL buffer)), before flash-freezing as pellets in liquid nitrogen.

Cells were lysed using a Freezer/Mill (6870D SPEX Sample Prep, 2 cycles, 1 min pre-cool, 2 min run-time, 1 min cool-time, rate 10 cps) before thawing in Lysis buffer. All subsequent steps were performed at 4°C unless specified otherwise. The lysate was clarified by centrifugation (160,000*g*, 45 min) and the supernatant filtered (0.45 μm PVDF syringe filters, Elkay Laboratory Products UK). The supernatant was then incubated with 8-10 mL anti-FLAG M2 affinity agarose gel (Sigma), rotating at 7 rpm for 60-90 min. The next affinity chromatography steps were done at room temperature using ice-cold buffers, unless stated otherwise. The supernatant was split between gravity flow columns (14 cm Econo-Pac, BioRad) and the flow-through re-applied once before each column was washed twice with 30 mL buffer W (40 mM HEPES-NaOH, pH 7.5, 150 mM NaOAc, 10% v/v glycerol, 0.005% v/v TWEEN 20, 0.5 mM TCEP). Each column was then washed once with 12.5 mL buffer W + 500 μM ATP + 5 mM Mg(OAc)_2_, incubating for 5 min partway through, before a final wash with 20 mL of buffer W + 2 mM CaCl_2_. Protein was eluted by successive addition of one CV buffer W + 2 mM CaCl_2_ + 0.2 mg/mL 3xFLAG peptide [Sigma], followed by two CV buffer W + 2 mM CaCl_2_ + 0.1 mg/mL 3xFLAG peptide, and finally one CV of buffer W + 2 mM CaCl_2_.

The FLAG-eluate was pooled and incubated with up to 1.2 mL Calmodulin Sepharose 4B affinity resin (GE Healthcare), rotating at 7 rpm for 1 h at 4°C. The sample was applied to a gravity flow column (9 cm Poly-Prep Chromatography Columns, Bio-Rad) and the flow-through reapplied twice. The column was washed twice with 20 mL buffer C (25 mM HEPES-NaOH, pH 7.5, 150 mM NaOAc, 0.5 mM TCEP) + 2 mM CaCl_2_ before elution using 3-5 mL buffer C + 2 mM EDTA + 2 mM EGTA. The sample was concentrated to 300 μL using 0.5 mL 30K MWCO centrifugal filters (Amicon) in a bench-top centrifuge (21,000*g*, 4°C). The sample was split across two glycerol gradients prepared as described above, with one gradient containing glutaraldehyde and used for subsequent sample preparation steps, while the second gradient lacked cross-linking agents to allow assessment of complex migration. The sample was separated by centrifugation in an SW 40 Ti rotor (Beckman) at 140,000*g* for 15 h at 4°C. Samples were manually fractionated in 400 μL fractions and analyzed by SDS-PAGE. The relevant fraction was buffer exchanged in buffer C + 0.005% v/v TWEEN 20 (Sigma, Cat# P8341) over six rounds of centrifugation (21,300*g*, 1 min/round, 4°C) in 0.5 mL 30K MWCO centrifugal filters (Amicon). The sample was concentrated to a final volume of ∼35 μL.

In early cryo-EM datasets we observed higher compositional heterogeneity of the complex likely arising from endogenous DNA co-purifying with our sample. In an attempt to overcome this, later sample preparations contained 10 μL streptavidin-blocked fork DNA added to the relevant fraction taken from glycerol gradients and incubated on ice for 15 min after gradient fixation and before buffer exchange. To prepare streptavidin-blocked fork DNA, 10 μL fork DNA (26.5 μM) was incubated with 12.5 μL tetravalent streptavidin (21 μM, Pierce) at room temperature for 40 min prior to addition of DNA to a purified replisome. The addition of DNA after the final centrifugation step did not alter DNA homogeneity in our cryo-EM reconstructions, and therefore data obtained from samples prepared with and without added streptavidin-blocked fork DNA were combined during processing.

#### Co-expression and purification of non-cross-linked CMG-Csm3/Tof1-Mrc1-Ctf4 complexes for cryo-EM

One sample was prepared as described for the co-expressed sample above (with SA-blocked fork DNA added) except with cross-linker omitted from the glycerol gradient. This was used to assess the impact of cross-linker on the architecture of the complex (Figure S3I).

#### Cryo-EM grid preparation

##### Reconstituted and co-expressed complex

Quantifoil R2/2, Cu-400 mesh cryo-EM grids pre-coated with an ultra-thin (3-5 nm) amorphous carbon (produced at the LMB) were glow discharged for 5 s at a plasma current of 15 mA (PELCO easiGlow). Sample (3 μL) was applied and incubated for 15-30 s at 4°C before manually blotting with filter paper for 10 s and plunge-freezing in liquid ethane (approx. −180°C).

#### Data collection

##### Reconstituted sample

A total of 6,878 raw micrographs were acquired across two datasets on the same 300 keV FEI Titan Krios microscope (LMB Krios3) at a calibrated pixel size of 1.049 Å/pixel (nominal magnification of 130,000 X). The K2 Summit direct electron detector (Gatan) was used in electron counting mode with a GIF Quantum energy filter slit width of 20 eV. EPU (ThermoFisher) was used for automated data collection, with a defocus range set at −1.4 to −2.6 μm and dose-fractionation into 20 fractions per movie, with a total exposure time of 7-8 s to achieve a dose of 37-38 e^-^/Å^2^ per micrograph.

##### Co-expressed sample

Six datasets were collected totaling 11,637 raw micrographs. The 300 keV FEI Titan Krios microscopes (LMB Krios1 and Krios2, eBIC Krios M03 and ESRF Krios1) were used with either a Falcon III direct electron detector (FEI) or a K2 Summit direct electron detector (Gatan), both in electron counting mode. The data were acquired at several magnifications ranging from 1.05-1.07 Å/pixel. EPU (ThermoFisher) was used for automated data collection with a defocus range set to −1.5 to −3 μm. For data acquired with a Falcon III detector the acquisition was dose-fractionated into 180 fractions with an exposure time of 44 s per micrograph and a dose of 0.82 - 0.84 e^-^/pixel/s. Data collected with a K2 camera were dose-fractionated into 20 fractions with a total exposure time of 6-8 s to achieve a dose per micrograph of 37-43 e^-^/Å^2^. The slit width of the GIF Quantum energy filter was set to 20 eV.

##### Co-expressed sample prepared without cross-linking

A single dataset of 2,527 raw micrographs was collected on a 300 keV Titan Krios microscope (LMB Krios2) equipped with a Falcon III direct electron detector (FEI) operated in electron counting mode and with a pixel size of 1.07 Å/pixel (nominal magnification of 75,000 X). EPU (ThermoFisher) with on-the-fly motion correction was used for automated data acquisition with a defocus range set at −2 to −3 μm, dose-fractionating each micrograph into 180 fractions. An exposure time of 44 s was used with a dose of 0.82 e^-^/pixel/s.

#### Data processing and 3D-reconstruction for the reconstituted sample

The gain-corrected 20-frame movies were aligned and dose-weighted (0.25-0.27 e^-^/Å^2^/frame) by MotionCor2 (Zheng et al., 2017). The contrast transfer function (CTF) parameters were calculated using Gctf (Zhang, 2016). Gautomatch (https://www.mrc-lmb.cam.ac.uk/kzhang/Gautomatch/) was used for automated particle picking on the remaining 6682 micrographs after manually discarding those containing contamination, no particles, significant drift or damaged holes. RELION 3.0-alpha was used for the entire data processing (Nakane et al., 2018, Scheres, 2012a, Scheres, 2012b, Zivanov et al., 2018). 632,000 particles were extracted with down-sampling by a factor of 2 and submitted for four rounds of 2D classification from which 472,000 selected particles were re-extracted without down-sampling in a box size of 360 pixels and submitted for 3D classification using a regularization parameter (T) of 4. Four of eight good 3D classes were included in further processing (Figure S2).

Two classes containing the best Csm3/Tof1 density (nearly 300,000 particles, 60%) were combined and 3D-refined before performing further rounds of CTF refinement, Bayesian polishing (Zivanov et al., 2019) and 3D-refinement to yield a map at an overall 3.1 Å resolution (all resolutions hereafter calculated with Gold standard Fourier shell correlation of 0.143). The precise pixel size of 1.049 Å was determined after maximizing the cross-correlation coefficient between our 3.1 Å map and the CMG:DNA model (PDB: 5U8S) (Georgescu et al., 2017) using Chimera (Pettersen et al., 2004). This pixel size was then used for postprocessing all maps obtained in this dataset. To further improve the Csm3/Tof1 density, multi-body refinement (Nakane et al., 2018) was performed for (i) the Tof1 Head including N-tier regions of Mcm2, 3, 5, and (ii) the Tof1 Body/Csm3 including the N-tier regions of Mcm4, 6, 7 and dsDNA (Figure S2). Resulting maps were sharpened with B-factor −20 Å^2^ to give final maps of 3.3 and 3.2 Å resolution, respectively. These maps were used for building the models of Csm3, Tof1 and dsDNA.

For the remainder of the complex, the above 3D classification identified two conformations differing in the position of the MCM C-tier and bound ssDNA (conformations 1 and 2). One class represented complexes in conformation 1 and containing Csm3/Tof1 (124,000 particles; 26%). One class represented complexes in conformation 2 and containing Csm3/Tof1 (159,000 particles, 34%). A third class represented a mixed population of particles in both conformations, lacking clear density for Csm3/Tof1; this class was separated into conformation 1 (74,000 particles; 16%) and conformation 2 (23,000 particles; 5%) using 3D subclassification without alignment.

For conformation 1 (Figure S2, gray maps), all classes in this conformation were combined irrespective of Csm3/Tof1 occupancy (198,000 particles; 42%) and 3D-refined, before performing CTF refinement, Bayesian polishing, another round of 3D refinement and map sharpening to yield a map at 3.2 Å resolution. Multi-body refinement was performed masking more rigidly-associated regions of the complex as described in Figure S2. After map sharpening, the resulting maps were used to build the atomic models of CMG and Ctf4 for conformation 1. The above multi-body refinement maps were sharpened with the following B-factors: −40 Å^2^ for the Mcm2356 map, −5 Å^2^ for the Mcm47 map, −20 Å^2^ for the remaining bodies.

For conformation 2 (Figure S2, yellow maps), a similar approach was taken as for conformation 1. After multi-body refinement, fitting of models to the conformation 2 density confirmed the only major differences between conformations was the position of the C-tier and bound ssDNA. Consequently, the maps for the MCM C-tier, Mcm3467 and Mcm25 were sharpened with B-factors −20, −10 and −10 Å^2^ respectively, and used to build the model of the MCM C-tier in conformation 2. An additional map produced after a further round of 3D subclassification (see below) was also used to aid model building for conformation 2.

After initial model building, it was clear there was a mixed population differing in AMP-PNP occupancy for conformation 2. To resolve these populations, the good particles from the original 3D-classification were combined before performing a further round of 3D-subclassification using a higher value T of 10 and limiting the Fourier components used in alignment to 10 Å resolution (refer to Figure S2). Of 12 classes, one represented complexes in conformation 2 with five AMP-PNP molecules bound to the C-tier (Figure S2, magenta map), and a second with particles in conformation 2 with three AMP-PNP molecules bound and a shorter region of ssDNA resolved (Figure S2, cyan map). Models were fitted to these and the AMP-PNP occupancy and ssDNA length adjusted accordingly (presented in Figure 3B). The map with five AMP-PNP molecules bound was then submitted for two-body multi-body refinement with one body covering Mcm25 and Mcm6 CTD; after map sharpening with a B-factor of −5 Å^2^, this map was useful in aiding final model building for the model of conformation 2. Finally it is worth noting this further 3D-subclassification additionally yielded a 3.7 Å resolution sharpened map of conformation 1 with more homogeneous resolution across all subunits in the complex.

To produce the cryo-EM density map best illustrating regions of unassigned density (Figure S3M) the subset of particles in conformation 2, which produced the 3.3 Å map of the whole complex (see Figure S2, yellow map), was subjected to a further round of 3D sub-classification without alignment, this time utilizing a higher value T of 100 in addition to providing a mask which encompassed Csm3/Tof1, dsDNA and the N-tier regions primarily belonging to Mcm4 and 6. Of six classes, four classes (50% of input particles) contained good Csm3/Tof1 and dsDNA density; these were recombined, 3D-refined and finally sharpened with a B-factor of −5 Å^2^.

Local resolution was calculated using RELION and maps were colored accordingly using Chimera (Pettersen et al., 2004), presented in Figure S1K.

#### Data processing and 3D-reconstruction for the cross-linked co-expressed sample

A total of six datasets totaling 11,647 raw movies were collected and processed independently using first RELION-2.1 and then RELION 3.0-alpha (Scheres, 2012a, Scheres, 2012b, Zivanov et al., 2018). In general, raw movies were aligned and dose-weighted by MotionCor2 (Zheng et al., 2017) and CTF parameters were estimated using Gctf (Zhang, 2016). Poor micrographs (containing contamination, no particles, significant drift or damaged holes) were manually excluded from each dataset. All particles were picked using Gautomatch (https://www.mrc-lmb.cam.ac.uk/kzhang/Gautomatch/). After one to two rounds of 2D-classification, followed by 3D-classification and 3D-refinement, the sharpened maps for the best classes from all six datasets were compared in Chimera in order to calculate scaling factors necessary for combining the datasets initially acquired at different microscope magnifications. Refined particles from five datasets were rescaled to the relative pixel size of the sixth dataset at 1.11 Å/pixel after re-estimation of the CTF parameters followed by particle re-extraction using a box size of 360 pixels (Wilkinson et al., 2019). The combined dataset comprised 412,000 particles, which were submitted for 3D-refinement. The resulting 3.4 Å map was sharpened with a B-factor of −20 Å^2^ and is presented in Figure S3J (see also Figures S3E and S3F). To improve the resolution of the complex, a three-body multi-body refinement was performed with bodies encompassing either the MCM C-tier, Csm3-Tof1-dsDNA or the remainder; the sharpened maps are presented in Figure S3G.

#### Data processing and 3D-reconstruction for the non-crosslinked co-expressed sample

Micrographs were processed using RELION-2.1 (Scheres, 2012a, Scheres, 2012b, Zivanov et al., 2018). Raw movies were aligned and dose-weighted by MotionCor2 (Zheng et al., 2017) and CTF parameters were estimated using Gctf (Zhang, 2016). Poor micrographs (containing contamination, no particles, significant drift or damaged holes) were manually excluded from each dataset. Particles were picked from the remaining 2387 micrographs using Gautomatch (https://www.mrc-lmb.cam.ac.uk/kzhang/Gautomatch/). A total of 413,000 particles were extracted and down-sampled by a factor of two before submission to two rounds of 2D classification. It was clear from 2D classification that there was a significant number of particles comprising more than one CMG molecule. Classes were stringently selected to contain complexes with a single copy of CMG, yielding 70,000 particles. These were subsequently submitted for 3D classification across six classes: heterogeneity was observed for Csm3-Tof1 and Ctf4 occupancy, with one class representing particles containing CMG-Csm3-Tof1-Ctf4-DNA. This class (28,000 particles) was re-extracted without down-sampling, 3D-refined and sharpened (B-factor of −50 Å^2^) to yield a map at 5.1 Å resolution (presented in Figure S3I).

#### Model building and refinement

Model building was carried out in *Coot* (Emsley et al., 2010) for the reconstituted sample using maps generated by multi-body refinement, as detailed in the data processing sections. An atomic model was built for conformation 1 (Table S1). As initial template models, CMG:DNA (PDB: 5U8S) (Georgescu et al., 2017) and the crystal structure of the C-terminal regions of Ctf4 (PDB: 4C8H) (Simon et al., 2014) were used where individual subunits were fitted as rigid bodies to the relevant multi-body refinement maps using Chimera (UCSF) (Pettersen et al., 2004), with N- and C-tier regions of MCM subunits fitted separately. It was notable that the resolution of MCM C-tier subunits was variable, with Mcm2, 3, 5 and 6 (those binding AMP-PNP and ssDNA) better resolved than Mcm4 and 7. Subunits were then jiggle-fitted and morphed to the relevant maps in *Coot*, prior to adjusting the models to density manually using local refinement and regularization.

For Mcm subunits, the regions N-terminal to the helical domain (N-terminal extension, NTE) of Mcm2, 4 and 6 were extended, with 28 residues built for the Mcm2 NTE, 12 residues built for the Mcm6 NTE, and remodelling of 6 residues of the Mcm4 NTE (Figure S1M). These NTE regions contain Tof1 binding sites. 172 residues were not observed for the NTE of Mcm2, although unassigned density is present in the vicinity and may account for some of these residues.

The MCM Zinc-finger (ZnF) domains were rebuilt with tetrahedrally-coordinated Zn^2+^ ions placed in the spherical density that was observed at low contour level between four cysteine residues in each of the ZnF domains in Mcm2, 4, 5, 6 and 7. The Mcm5 ZnF was based on the MCM double hexamer template model (PDB: 5BK4) (Noguchi et al., 2017). The N-terminal hairpin (NTH) loops of Mcm7 (the separation pin, 362-368) and Mcm2 (436-443) were remodelled as α-helical, with the Mcm6 NTH also significantly remodelled. The linkers between N- and C-tier were built fully as loops for Mcm2 (459-476) and 5 (339-363) and an α-helix (501-508) was built for the mostly disordered N/C-tier linker of Mcm6 (463-509). In the C-tier, the ssDNA-binding regions (helix H2, H2I loops and PS1 loops) showed much improved density, which required significant remodelling in terms of repositioning and extending the Cα-backbone, assigning the correct sequence register and rebuilding side-chains.

Density observed around the C-tier region of Mcm3 where the model is incomplete (vicinity of residue 583) remains unassigned. Additional helical density observed in the vicinity of Mcm6 could be potentially attributed to residues 202-251 and/or its N/C-tier linker, however this region was not included in the final model. The C-terminal winged-helix (WH) domain (851-877) was retained for Mcm4 in the lower-resolution density, as seen in prior structural work (Georgescu et al., 2017). AMP-PNP/Mg^2+^ was built in well resolved density at the Mcm2/6, 2/5 and 3/5 interfaces with side chains visible for WalkerA, WalkerB, Arg-finger and Sensor2 motifs (Figure S1H).

For the remainder of CMG, model building was as follows. The N-terminal CIP-box of Sld5 taken from the crystal structure of this peptide bound to Ctf4 (PDB: 4C95) (Simon et al., 2014) was rigid-body fitted and adjusted in clearly visible density. The model for Psf2 was extended for additional residues 33-38, which now appear to be ordered, presumably through the interaction of this region with the Ctf4 helical bundle. For Ctf4, the side-chains were adjusted, particularly at the interface with Cdc45 and Psf2. The N-terminal regions of Ctf4 (1-460), known to contain a WD40 domain in human And-1, could not be assigned to specific regions of density in our complex. Five residues of the Psf3 N-terminal His-tag were resolved in the density and are present in the model (N-Ser-His-Met-Ala-Ser-C).

For conformation 2, the largest changes compared to conformation 1 were observed in the MCM C-tier and the length of bound ssDNA, therefore a model was built for this region by adjusting our MCM C-tier models for conformation 1 to density of conformation 2. The resolution of C-tier subunits varied, with those bound to AMP-PNP/Mg^2+^ and 16-mer ssDNA (built as poly-dT) better resolved (the only AMP-PNP-free interface was observed between Mcm2 and 5). The major differences compared to conformation 1 were observed in the relative positions of individual MCM subunits and the positions of the ssDNA-binding loops. For Mcm4, density for the WH was no longer observed, while the linker connecting the WH to the AAA+ domain was repositioned away from the MCM central channel.

Csm3/Tof1 was partially built *de novo*. The N terminus of Tof1 was identified in our density after rigid-body fitting of the fragment of human Timeless (PDB: 5MQI) (Holzer et al., 2017), which was then used for homology modeling of the Tof1 region comprising helical repeats 1-6 using Phyre2 (Kelley et al., 2015). The Tof1 homology model was morphed and jiggle-fitted into our multi-body map of the Tof1 Head (Figure S2), which was then manually adjusted and locally refined before building *de novo* the Ω-loop and the MCM-plugin, which extend between helical repeats 3-4 and 4-5, respectively. The Mcm6 NTE packs against the Ω-loop and this region was built as a composite Mcm6-Tof1 β sheet given good density. The density for the Ω-loop in the region facing the major groove of DNA indicates greater flexibility. The density and connectivity for the long MCM-plugin was of a good quality, in particular several prominent newly built secondary structure elements (helices Bridge and αW, and the Wedge β-hairpin, see Figure S7E) packing against the interface with Mcm6, 4 and 7. The density of the MCM-plugin which protrudes into the minor groove of DNA could be well resolved and the side chains built represent those of the Tof1 DBM (401-404). Repeats 7-9 of Tof1 (the Body encompasses repeats 6-9, Figure S7A) were built *de novo* up to residue 781 with certain loops being omitted due to lack of density (Table S1). Following residue 781, the remaining novel density accounting for five helices was observed to have opposite polarity to helices in the Tof1 Body and the model for this density was built *de novo* with the sequence register assigned to the core of Csm3; in particular, the side chains of the helix α2 were well resolved with a prominent tryptophan side chain (W98). Additional density extending from a small helix α0 into the minor groove of dsDNA was built as the Csm3 DBM (46-53). This region is predicted to be disordered and is likely stabilized by interaction with DNA.

The dsDNA was built in the density of a multi-body refinement map representing Tof1^Body^/Csm3/dsDNA. Sequence register was assigned based on the sequence of our fork DNA assuming no unwinding due to the inclusion of AMP-PNP during sample preparation.

Once rebuilt, subunits were refined in the relevant maps using Refmac (Kovalevskiy et al., 2018), phenix.real_space_refine (Afonine et al., 2018) and ISOLDE (Croll, 2018).

#### Model to cryo-EM map validation for conformation 1 and conformation 2

Fourier Shell Correlation (FSC) was calculated between the refined models (conformation 1 and MCM C-tier:ssDNA of conformation 2) and their respective unsharpened sums of the two half maps using XMIPP (Sorzano et al., 2004). The above models were also refined with restraints against the respective half-1 maps and the FSC map-to-model curves were calculated for the half-1 and half-2 (not used for model refinement) maps (Figure S1I).

#### Cross-linking mass spectrometry (XL-MS)

The complex comprising CMG, Ctf4, Csm3/Tof1 and Mrc1 was purified following co-expression as described above. The eluate from the Calmodulin Sepharose 4B column (25 mM HEPES pH 7.5, 150 mM sodium acetate, 0.5 mM TCEP, 2 mM EDTA/EGTA) was immediately cross-linked with a 100-fold excess of the N-hydroxysuccinimide (NHS) ester disuccinimidyl dibutyric urea (DSBU, ThermoScientific, USA), with respect to the protein concentration. The cross-linking reactions were incubated for 60 min at room temperature and then quenched by the addition of NH_4_HCO_3_ to a final concentration of 20 mM and incubated for further 15 min. The cross-linked proteins were then precipitated according to the method of Wessel and Flügge (1984) and resuspended in 8 M urea in 50 mM NH_4_HCO_3._

The cross-linked proteins were reduced with 10 mM DTT and alkylated with 50 mM iodoacetamide. Following alkylation, the concentration of urea was reduced to 1 M by the addition of 50 mM NH_4_HCO_3_ and the proteins digested with trypsin (Promega, UK) at an enzyme-to-substrate ratio of 1:100, for 1 h at room temperature and then further digested overnight at 37°C following a subsequent addition of trypsin at a ratio of 1:20.

The peptide digests were then fractionated batch-wise by high pH reverse phase chromatography on micro spin C18 columns (Harvard Apparatus, USA), into five fractions (10 mM NH_4_HCO_3_ /10% v/v acetonitrile pH 8, 10 mM NH_4_HCO_3_ /20% v/v acetonitrile pH 8, 10 mM NH_4_HCO_3_ /30% v/v acetonitrile pH 8, 10 mM NH_4_HCO_3_ /50% v/v acetonitrile pH 8 and 10 mM NH_4_HCO_3_ /80% v/v acetonitrile pH 8). The 150 μL fractions were evaporated to dryness on a CoolSafe lyophilizer (ScanVac, Denmark) prior to analysis by LC-MS/MS.

Lyophilized peptides for LC-MS/MS were resuspended in 0.1% v/v formic acid and 2% v/v acetonitrile and analyzed by nano-scale capillary LC-MS/MS using an Ultimate U3000 HPLC (ThermoScientific Dionex, USA) to deliver a flow of approximately 300 nl/min. A C18 Acclaim PepMap100 5 μm, 100 μm × 20 mm nanoViper (ThermoScientific Dionex, USA), trapped the peptides before separation on a C18 Acclaim PepMap100 3 μm, 75 μm × 250 mm nanoViper (ThermoScientific Dionex, USA). Peptides were eluted with a gradient of acetonitrile. The analytical column outlet was directly interfaced via a nano-flow electrospray ionisation source, with a quadrupole Orbitrap mass spectrometer (Q-Exactive HFX, ThermoScientific, USA). MS data were acquired in data-dependent mode using a top 10 method, where ions with a precursor charge state of 1+ and 2+ were excluded. High-resolution full scans (R = 120 000, m/z 300-1800) were recorded in the Orbitrap followed by higher energy collision dissociation (HCD) (stepped collision energy 26 and 28% Normalized Collision Energy) of the 10 most intense MS peaks. The fragment ion spectra were acquired at a resolution of 50,000 and dynamic exclusion window of 20 s was applied.

For data analysis, Xcalibur raw files were converted into the MGF format using MSConvert (Proteowizard) (Kessner et al., 2008) and used directly as input files for MeroX (Götze et al., 2015). Searches were performed against an *ad hoc* protein database containing the sequences of the proteins in the complex and a set of randomized decoy sequences generated by the software. The following parameters were set for the searches: maximum number of missed cleavages 3; targeted residues K, S, Y and T; minimum peptide length 5 amino acids; variable modifications: carbamidomethylation of cysteine (mass shift 57.02146 Da), Methionine oxidation (mass shift 15.99491 Da); DSBU modified fragments: 85.05276 Da and 111.03203 Da (precision: 5 ppm MS and 10 ppm MS/MS); False Discovery Rate cut-off: 5%. Finally, each fragmentation spectrum was manually inspected and validated.

#### Origin-dependent DNA replication assays

Origin-dependent replication assays were performed essentially as described previously (Aria and Yeeles, 2018, Taylor and Yeeles, 2018). MCM loading was performed at 24°C in reactions (typically 35 μl) containing 25 mM HEPES-KOH pH 7.6, 100 mM K-glutamate, 0.01% v/v Nonidet P40 substitute (NP-40-S) (Roche #11754599001), 1 mM DTT, 10 mM Mg(OAc)_2_, 40 mM KCl, 0.1 mg/ml BSA, 3 mM ATP, 3 nM AhdI-linearized vVA20 template (Aria and Yeeles, 2018), 75 nM Cdt1⋅Mcm2-7, 40 nM Cdc6, 25 nM DDK, 20 nM ORC. After 10 min S-CDK was added to a final concentration of 80 nM and incubation continued at 24°C for 5 min. Reactions were diluted 4-fold into replication buffer to give final reaction concentrations (accounting for subsequent addition of replication proteins) of 25 mM HEPES-KOH pH 7.6, 250 mM K-glutamate, 0.01% NP-40-S, 1 mM DTT, 10 mM Mg(OAc)_2_, 10 mM KCl, 0.1 mg/ml BSA, 3 mM ATP, 200 μM C/G/UTP, 30 μM dA/dT/dG/dCTP, 1 μCi [α-^32^P]-dCTP, 0.75 nM AhdI-linearized vVA20 template, 18.75 nM Cdt1⋅Mcm2-7, 10 nM Cdc6, 6.25 nM DDK, 5 nM ORC. Reactions were equilibrated at 30°C (∼1 min) and replication initiated by addition of replication proteins from a master mix to the following final concentrations: 30 nM Dpb11, 100 nM GINS, 30 nM Cdc45, 10 nM Mcm10, 20 nM Pol ε, 20 nM Ctf4, 100 nM RPA, 20 nM RFC, 20 nM PCNA, 20 nM Pol α, 10 nM Pol δ, 12.5 nM Sld3/7, 20 nM Sld2, 20 nM Mrc1 and 20 nM Csm3/Tof1 or mutants where indicated. Reactions were quenched by addition of an equal volume of 100 mM EDTA and samples were processed as previously described (Aria and Yeeles, 2018, Taylor and Yeeles, 2018). RFB experiments were performed on AhdI-linearized vJY30 (Table S3) and Fob1 was added together with the MCM loading proteins to a concentration of 250 nM (62.5 nM after dilution into replication buffer).

#### Replisome association assays

MCM loading was performed at 30°C in reactions containing 25 mM HEPES-KOH pH 7.6, 100 mM K-glutamate, 0.01% v/v NP-40-S, 1 mM DTT, 10 mM Mg(OAc)_2_, 0.1 mg/ml BSA, 3 mM ATP, 3 nM vVA20 template (Aria and Yeeles, 2018), 75 nM Cdt1⋅Mcm2-7, 40 nM Cdc6, 25 nM DDK, 14 nM ORC. After 30 min reactions were diluted 2-fold into replication buffer to give final reaction concentrations (accounting for subsequent addition of replication proteins) of 25 mM HEPES-KOH pH 7.6, 250 mM K-glutamate, 0.01% NP-40-S, 1 mM DTT, 10 mM Mg(OAc)_2_, 0.1 mg/ml BSA, 3 mM ATP, 200 μM C/G/UTP, 30 μM dA/dT/dG/dCTP, 1.5 nM vVA20 template, 37.5 nM Cdt1⋅Mcm2-7, 20 nM Cdc6, 12.5 nM DDK, 7 nM ORC. Replication was initiated by addition of replication proteins from a master mix to the following final concentrations: 30 nM Dpb11, 100 nM GINS, 30 nM Cdc45, 10 nM Mcm10, 20 nM Pol ε, 20 nM Ctf4, 100 nM RPA, 20 nM RFC, 20 nM PCNA, 20 nM Pol α, 12.5 nM Sld3/7, 20 nM Sld2, 10 nM Mrc1 and 20 nM Csm3/Tof1 or mutants where indicated. After 25 min, samples (13 μl) were directly applied to 400 μL (bed volume) Sephacryl S-400 columns (GE healthcare) equilibrated in 25 mM HEPES-NaOH pH 7.5, 150 mM NaOAc, 10 mM Mg(OAc)_2_, 1 mM DTT, 0.01% v/v NP-40-S and 0.1 mM ATP. Columns were centrifuged (750 g, 2 min, 21°C) and the eluate was analyzed by SDS-PAGE and western blotting. Cdc45 was detected using its FLAG epitope with an anti-FLAG antibody (A8592, Sigma). RPA was detected with an antibody against the Rpa1 subunit (AS07 214). Mcm7, Psf1, Ctf4, Csm3 and Mrc1 were detected with sheep polyclonal antibodies (Maric et al., 2014, Mukherjee and Labib, 2019).

#### Electrophoretic mobility-shift assays

Csm3/Tof1 wild-type or mutant proteins were mixed with fork DNA prepared as for cryo-EM sample preparation (40 nM final [DNA]), at a molar ratio of protein:DNA of 1:1, 2:1, 4:1 and 8:1 in a reaction buffer containing 25 mM HEPES-KOH, pH 7.6, 100 mM KOAc, 2 mM Mg(OAc)_2_, 0.2% NP40 and 1 mM DTT and incubated on ice for 30 min. Ficoll 400 was added to each 15 μL reaction to a final concentration of 2.3% v/v before loading onto 4% native polyacrylamide gels for analysis. Gels were imaged using a Typhoon fluorescence imager (Amersham) at the Cy3 excitation wavelength of 532 nm.

#### Phosbind SDS-PAGE

Prior to electrophoresis Csm3/Tof1 was treated with Lambda protein phosphatase (λ-PP) in a reaction (100 μl) containing 50 mM HEPES-NaOH pH 7.5, 100 mM NaCl, 2 mM DTT, 1 mM MnCl_2_, 300 nM Csm3/Tof1 and 0.1 mg/ml λ-PP (Zhuo et al., 1993) for 40 min at 37°C. Samples (10 μl) were separated through 5% polyacrylamide gels containing 353 mM Bis-Tris-HCL pH 6.8, 100 μM ZnCl_2_ and 50 μM Phosbind (APExBio). Control gels were also run in the absence of Phosbind. Electrophoresis was performed in 1x NuPAGE MOPS running buffer (Invitrogen) at 30 mA for ∼90 min and gels were stained with Coomassie InstantBlue (Expedeon).

#### Camptothecin sensitivity assays

Saturated cultures of *S. cerevisiae* grown in YEP + 2% w/v glucose were diluted to an A_600_ of 0.2 and were grown to an A_600_ of ∼0.6-0.8 in YEP + 2% w/v glucose at 30°C. Cells were harvested and resuspended in YEP + 2% w/v glucose + 100 μg/ml ampicillin or sterile water to a A_600_ of 0.5. Cells from a 10-fold serial dilution in YEP + 2% w/v glucose + 100 μg/ml ampicillin or sterile water were then plated (8 μl) on YEPD agar plates supplemented with either DMSO or camptothecin (Merck).

#### Multiple sequence alignments

Amino acid sequences were retrieved from relevant databases (NCBI or SGD where stated; UniProt otherwise) (Cherry et al., 2012, UniProt Consortium, 2019). Alignment was performed using MUSCLE (EMBL-EBI) (Edgar, 2004). The alignment was rendered using ESPript3.0 (http://espript.ibcp.fr) (Robert and Gouet, 2014).

#### Structural analysis and visualization

All figures of structures were plotted in PyMOL (SchrödingerLLC, 2015), Chimera (Pettersen et al., 2004) or ChimeraX (Goddard et al., 2018). Calculations of buried surface area were performed using PDBePISA (Krissinel and Henrick, 2007). XL-MS crosslinks mapped to the atomic model in Figures S3J and S3K were plotted using the UCSF Chimera (Pettersen et al., 2004) plugin Xlink Analyzer (Kosinski et al., 2015).

### Quantification and Statistical Analysis

Quantification and data analysis of replication assays were performed in ImageJ and Prism8. Lane profiles were generated in ImageJ and were used to quantify the intensity of the stalled Left leading strand and the Right leading strand. Normalized Stall was derived by dividing the intensity of the Stalled left leading strand by the intensity of the Right leading strand. Data were plotted in Prism8.

## Supporting Citations

The following references appear in the Supplemental Information: Enemark and Joshua-Tor, 2006, Hodgson et al., 2007, Holm and Sander, 1995, Iacobucci et al., 2018, Ishiyama et al., 2010, Itsathitphaisarn et al., 2012, Morohashi et al., 2009, Singleton et al., 2004, Thomsen et al., 2016, Velankar et al., 1999, Wilson et al., 1995.

## Supplemental Information

Document S1. Figures S1–S10 and Tables S1 and S3–S5

Table S2 Summary of Cross-Links Identified in Cross-Linking Mass Spectrometry Experiments, Related to Figure 2 The quality of the fragment ion assignment is measured by a scoring function (score) (Iacobucci et al., 2018). Briefly, all peptide pairs matching the identified reporter ions are subjected to scoring. The score is determined in relation to the presence and intensity of the DSBU reporter ions, the number and length of peptide backbone ion series, and the number of identified ions related to the spectrum size and the number of possible fragment ions created from a theoretical peptide pair. To correct for random overlaps, features are also calculated for spectra with slightly shifted mass values (Iacobucci et al., 2018). Only peptides used in analysis with a score of ≥60 are shown.

Document S2. Article plus Supplemental Information
