# Supplementary material for: Cryo-EM Structure of the Fork Protection Complex Bound to CMG at a Replication Fork
Source: Mol Cell. 2020 Jun 4;78(5):926–940.e13. doi: 10.1016/j.molcel.2020.04.012 (PMC7276988; doi:10.1016/j.molcel.2020.04.012)
Supplement: Document S1. Figures S1–S10 and Tables S1 and S3–S5 [file mmc1.pdf]

Molecular Cell, Volume *78*

Supplemental Information

Cryo-EM Structure of the Fork Protection Complex Bound to CMG at a Replication Fork

Domagoj Baretić, Michael Jenkyn-Bedford, Valentina Aria, Giuseppe Cannone, Mark Skehel, and Joseph T.P. Yeeles

Figure S1

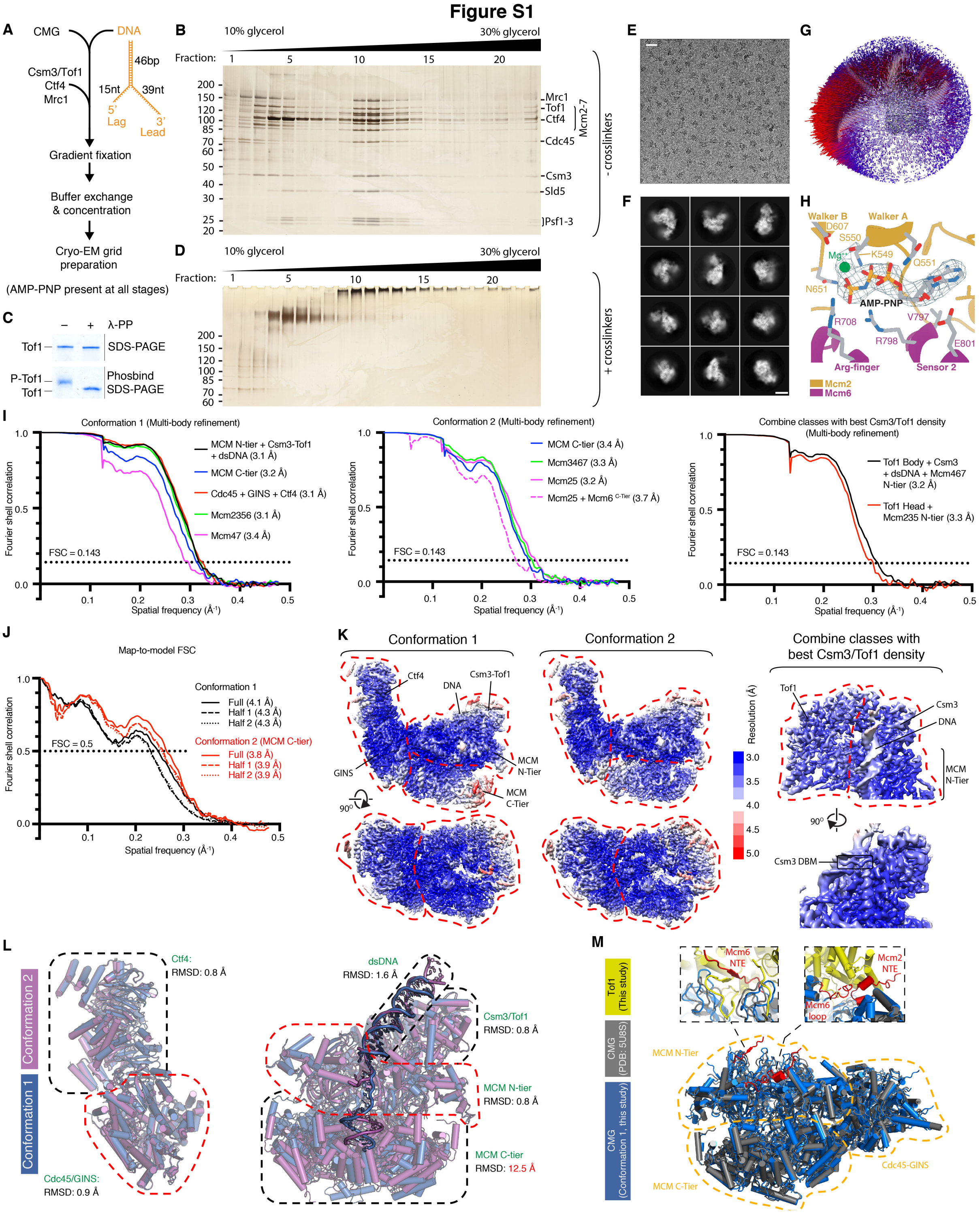

A
CMG
DNA
46bp
Csm3/Tof1
Ctf4
Mrc1
15nt
39nt
5' Lag
3' Lead
Gradient fixation
Buffer exchange & concentration
Cryo-EM grid preparation
(AMP-PNP present at all stages)
B
10% glycerol
30% glycerol
Fraction:
Mrc1
Tof1
Ctf4
Mcm2-7
Cdc45
Csm3
Sld5
Psf1-3
- crosslinkers
C
λ-PP
Tof1
SDS-PAGE
P-Tof1
Phosbind SDS-PAGE
D
+ crosslinkers
E
F
G
H
Walker B
Walker A
D607
S550
K549
Q551
N651
AMP-PNP
R708
V797
R798
E801
Arg-finger
Sensor 2
Mcm2
Mcm6
I
Conformation 1 (Multi-body refinement)
MCM N-tier + Csm3-Tof1 + dsDNA (3.1 Å)
MCM C-tier (3.2 Å)
Cdc45 + GINS + Ctf4 (3.1 Å)
Mcm2356 (3.1 Å)
Mcm47 (3.4 Å)
FSC = 0.143
Fourier shell correlation
Spatial frequency (Å-1)
Conformation 2 (Multi-body refinement)
MCM C-tier (3.4 Å)
Mcm3467 (3.3 Å)
Mcm25 (3.2 Å)
Mcm25 + Mcm6 C-Tier (3.7 Å)
Combine classes with best Csm3/Tof1 density (Multi-body refinement)
Tof1 Body + Csm3 + dsDNA + Mcm467 N-tier (3.2 Å)
Tof1 Head + Mcm235 N-tier (3.3 Å)
J
Map-to-model FSC
Conformation 1
Full (4.1 Å)
Half 1 (4.3 Å)
Half 2 (4.3 Å)
Conformation 2 (MCM C-tier)
Full (3.8 Å)
Half 1 (3.9 Å)
Half 2 (3.9 Å)
FSC = 0.5
K
Conformation 1
Conformation 2
Combine classes with best Csm3/Tof1 density
Ctf4
DNA
Csm3-Tof1
GINS
MCM N-Tier
MCM C-Tier
Resolution (Å)
Tof1
Csm3
MCM N-Tier
Csm3 DBM
L
Conformation 2
Conformation 1
Ctf4: RMSD: 0.8 Å
Cdc45/GINS: RMSD: 0.9 Å
dsDNA RMSD: 1.6 Å
Csm3/Tof1 RMSD: 0.8 Å
MCM N-tier RMSD: 0.8 Å
MCM C-tier RMSD: 12.5 Å
M
Tof1 (This study)
CMG (PDB: 5U8S)
CMG (Conformation 1, this study)
Mcm6 NTE
Mcm2 NTE
Mcm6 loop
MCM N-Tier
MCM C-Tier
Cdc45-GINS

Figure S1, related to Figure 1. Samples for cryo-EM experiments prepared by *in vitro* reconstitution.

(A) Schematic illustrating method of sample preparation for cryo-EM experiments.

(B) Representative silver-stained SDS-PAGE showing fractions taken across glycerol gradient prepared with omission of cross-linking agents.

(C) Analysis of Tof1 phosphorylation status. Tof1 was analyzed by Coomassie-stained SDS-PAGE in the absence and presence of Phosbind as indicated.

(D) As in (B) but for a gradient containing the cross-linking agents bis(sulfosuccinimidyl) suberate (BS^3) and glutaraldehyde. Fractions 10+11 were pooled for cryo-EM grid preparation.

(E) Representative cryo-EM micrograph. Scale bar, 30 nm.

(F) Representative 2D class averages. Scale bar, 10 nm.

(G) Representative 3D angular distribution of particle projections.

(H) Representative example of cryo-EM density observed for bound AMP-PNP•Mg^{2+}.

(I) Representative Fourier shell correlation (FSC) curves shown for maps used in model building. The FSC=0.143 criterion used to determine map resolution is indicated on all graphs (dotted black line). Left: conformation 1 (grey maps in Figure S2). Centre: conformation 2 (yellow maps in Figure S2). The Mcm25+$Mcm6^{C\text{-}tier}$ curve (dashed line) is derived from multi-body refinement of a separate 3D subclassification (refer to Figure S2). Right: maps used to build Csm3/Tof1 models (red maps in Figure S2).

(J) Model-to-map FSC curves. *Full*: correlation between the sum of half-map 1 and half-map 2, and the refined model. *Half 1*: correlation between half-map 1 and the model refined in half-map 1. *Half 2*: correlation between half-map 2 and the model refined in half-map 1.

(K) Representative cryo-EM density maps colored by local resolution. In each case, composite maps are displayed comprising separate maps derived from multi-body refinement, indicated by the red-dashed lines. Far-right: multi-body refinement maps used for Csm3/Tof1 model building derived from combining particles in conformations 1 and 2 (coloured red in Figure S2).

(L) Comparison of conformations 1 and 2. Models were built for the entire complex in conformation 1, and the MCM C-tier of conformation 2. To complete the model of conformation 2, remaining subunits were docked as rigid bodies with models fitting the density well. Complete models for conformations 1 and 2 were then aligned on either the MCM N-tier or Cdc45/GINS (red outlines) and root mean square deviation (RMSD) values calculated for the regions indicated (dashed outlines) without further alignment. The large movement in the C-tier resulting in the high RMSD value is illustrated in Movie S1.

(M) Accessory-factor binding does not induce large conformational changes in CMG helicase. CMG from conformation 1 (CMG-Csm3/Tof1-Mrc1-Ctf4-DNA) was compared to the published model of CMG-DNA (PDB: 5U8S) (Georgescu et al., 2017) by aligning on the MCM N-tier using PyMOL. Regions of Mcm2 and Mcm6 which became ordered by direct interaction with Tof1 are highlighted in red.

Figure S2

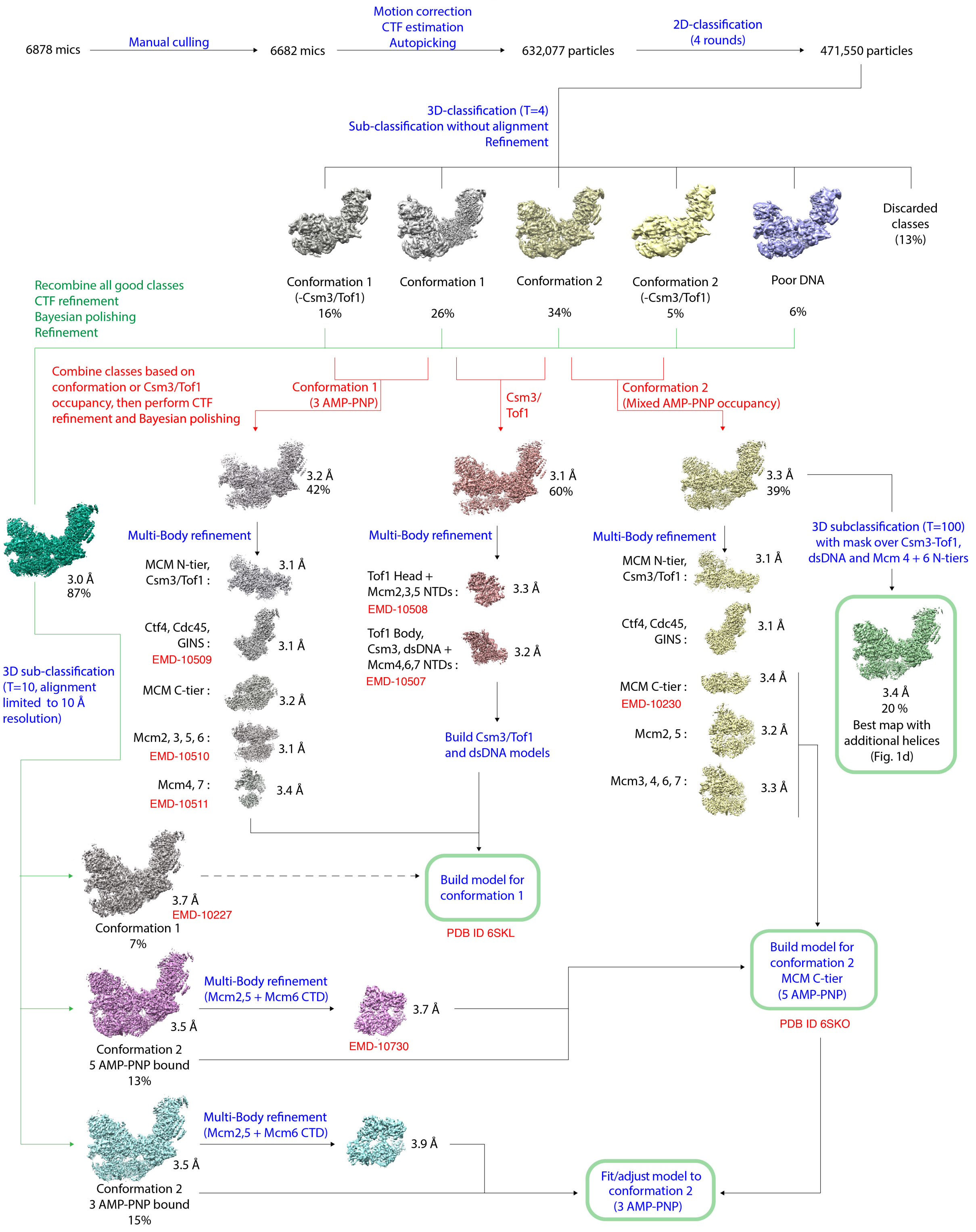

Figure S2, related to Figure 1. Schematic of data processing pipeline for cryo-EM samples prepared by *in vitro* reconstitution.

Cryo-EM density maps related to conformation 1 are colored grey. The 3.7 Å conformation 1 map represents the map with best density over all subunits in a single map for this conformation. Maps related to conformation 2 are colored yellow for maps with a mixed AMP-PNP occupancy, or magenta/cyan for maps with five/three AMP-PNP bound. T is the regularization parameter used during 3D classification. Green boxes highlight the final models discussed in the text and the map best illustrating regions of unassigned density (Figure S3J).

Figure S3

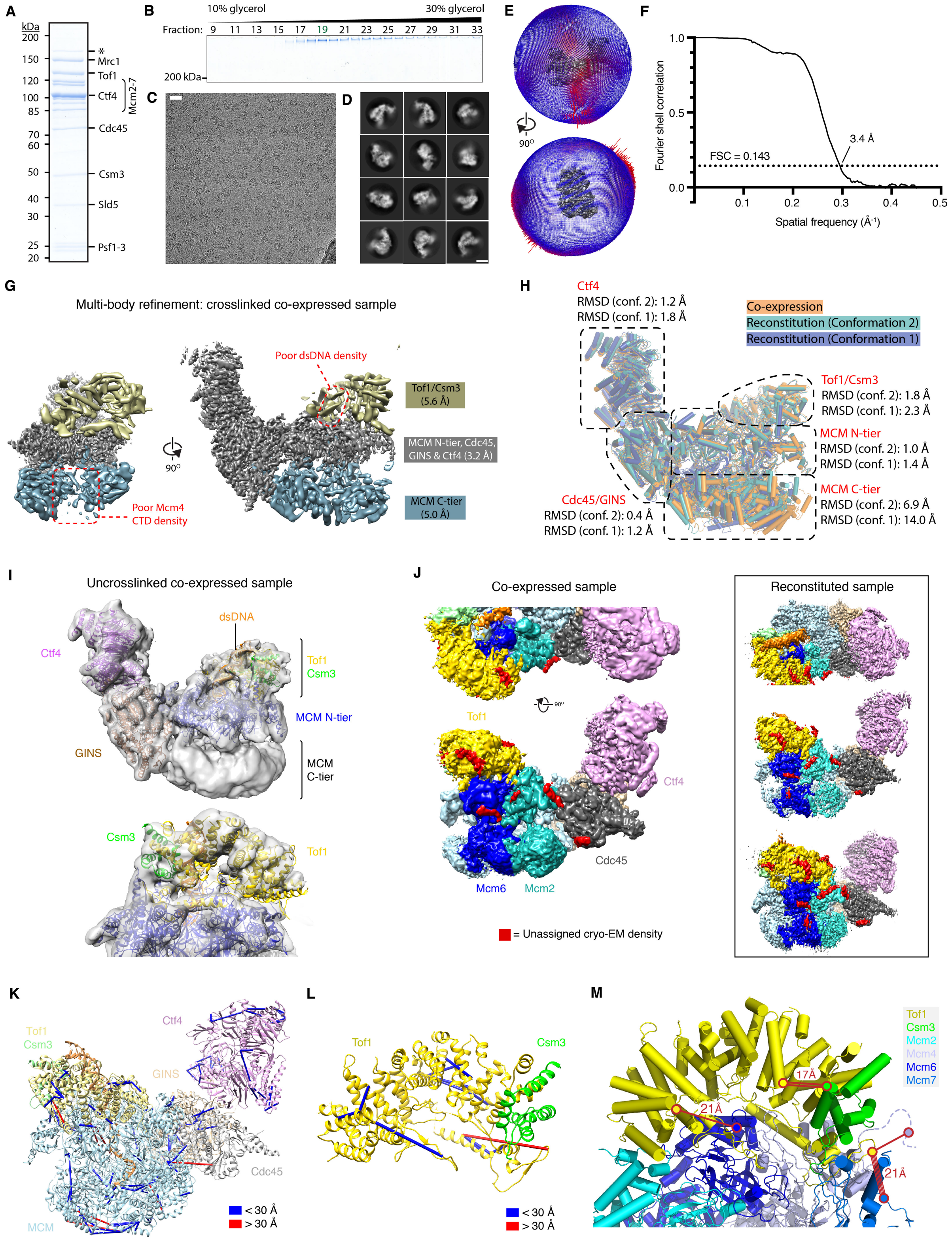

A
kDa
200
150
120
100
85
70
60
50
40
30
25
20
*
Mrc1
Tof1
Ctf4
Mcm2-7
Cdc45
Csm3
Sld5
Psf1-3
B
10% glycerol
30% glycerol
Fraction: 9 11 13 15 17 19 21 23 25 27 29 31 33
200 kDa
C
D
E
90°
F
1.0
0.5
0.0
Fourier shell correlation
FSC = 0.143
3.4 Å
0.1 0.2 0.3 0.4 0.5
Spatial frequency (Å-1)
G
Multi-body refinement: crosslinked co-expressed sample
Poor dsDNA density
Tof1/Csm3 (5.6 Å)
MCM N-tier, Cdc45, GINS & Ctf4 (3.2 Å)
MCM C-tier (5.0 Å)
Poor Mcm4 CTD density
90°
H
Ctf4
RMSD (conf. 2): 1.2 Å
RMSD (conf. 1): 1.8 Å
Co-expression
Reconstitution (Conformation 2)
Reconstitution (Conformation 1)
Tof1/Csm3
RMSD (conf. 2): 1.8 Å
RMSD (conf. 1): 2.3 Å
MCM N-tier
RMSD (conf. 2): 1.0 Å
RMSD (conf. 1): 1.4 Å
MCM C-tier
RMSD (conf. 2): 6.9 Å
RMSD (conf. 1): 14.0 Å
Cdc45/GINS
RMSD (conf. 2): 0.4 Å
RMSD (conf. 1): 1.2 Å
I
Uncrosslinked co-expressed sample
dsDNA
Ctf4
Tof1
Csm3
MCM N-tier
GINS
MCM C-tier
Csm3
Tof1
J
Co-expressed sample
Reconstituted sample
90°
Tof1
Ctf4
Cdc45
Mcm6
Mcm2
= Unassigned cryo-EM density
K
Tof1
Csm3
Ctf4
GINS
Cdc45
MCM
< 30 Å
> 30 Å
L
Tof1
Csm3
< 30 Å
> 30 Å
M
Tof1
Csm3
Mcm2
Mcm4
Mcm6
Mcm7
17Å
21Å
21Å

Figure S3, related to Figure 1. Samples for cryo-EM experiments prepared by co-expression of 15 proteins in *S. cerevisiae.*

(A, B) Representative Coomassie-stained SDS-PAGE of the eluate from calmodulin sepharose 4B resin (A) (see Methods for details) and fractions across a crosslinking glycerol gradient containing glutaraldehyde (B). In (B), fraction 19 was taken for cryo-EM grid preparation. The 14 mL gradient (400 μL fractions) used in (B) differs from the 2.2 mL gradient (100 μL fractions) used for the reconstituted sample (Figure S1B and S1D), explaining the discrepancy in the fraction number chosen. * indicates a contaminant with comparable migration to previously identified Pol1, a known interaction partner of Ctf4 shown to co-purify with replisome progression complexes under similar purification conditions (Gambus et al., 2006). All panels in Figure S3A-J (except Figure S3I) depict samples prepared with crosslinking.

(C) Representative cryo-EM micrograph. Scale bar, 30 nm.

(D) Representative 2D class averages. Scale bar, 10 nm.

(E) 3D angular distribution for particle projections.

(F) Fourier shell correlation curve for the best overall cryo-EM map.

(G) Cryo-EM maps produced using multi-body refinement, with individually refined regions (bodies) colored separately. Single-stranded DNA was not observed to bind the MCM C-tier, consistent with a mixed population of conformations 1 and 2 which were distinguished only after stabilizing the C-tier by addition of AMP-PNP to the reconstituted sample.

(H) Comparison of reconstitution and co-expression datasets illustrating that major differences are only observed in the MCM C-tier. The model for the co-expressed sample was generated by rigid body fitting of individual subunits from the reconstituted sample into the co-expression map, with N- and C-tier regions fitted independently for each Mcm. The complete model for conformation 2 was produced as described for Figure S1L. Complete models (except DNA) were aligned on the region encompassing MCM N-tier, Cdc45 and GINS, and RMSD values were determined for each region of the complex indicated by dashed outlines without further alignment.

(I) Cryo-EM density map for a sample produced by co-expression without addition of cross-linking agents. The model for conformation 1 (built using the high-resolution reconstituted dataset, see Figures 1, S1 and S2) with the C-tier deleted fitted the density well as a single rigid body indicating crosslinking did not affect the gross architecture of the complex.

(J) Cryo-EM density maps for the co-expressed and reconstituted samples coloured by subunit indicating the position of regions of unassigned density (red).

(K) Inter- and intra-subunit cross-links (score ≥ 60) detected by cross-linking mass spectrometry (XL-MS) mapped to the model of conformation 1. The distance expected between Cα atoms of the cross-linked residues was expected to be ~30 Å or less. The majority of crosslinking distances exceeding 30 Å can be rationalized by flexibility in the complex, primarily in the mobile MCM C-tier known to adopt multiple conformations, the flexibly tethered Mcm4 winged-helix domain observed to reposition in conformation 2, and loops which might reposition in a subset of particles.

(L) Intra-subunit cross-links detected by XL-MS for Csm3/Tof1 (score ≥ 60). Note the cross-linking distance > 30 Å was detected for a cross-link between two residues in the MCM-plugin which would become disordered if a sub-population of particles had dissociated from CMG during sample preparation or analysis.

(M) Inter-subunit cross-links detected by XL-MS for Csm3/Tof1 (score ≥ 60). The number of lines denotes the number of crosslinks detected. Circles denote the position of the residues observed to cross-link, coloured by subunit.

Figure S4

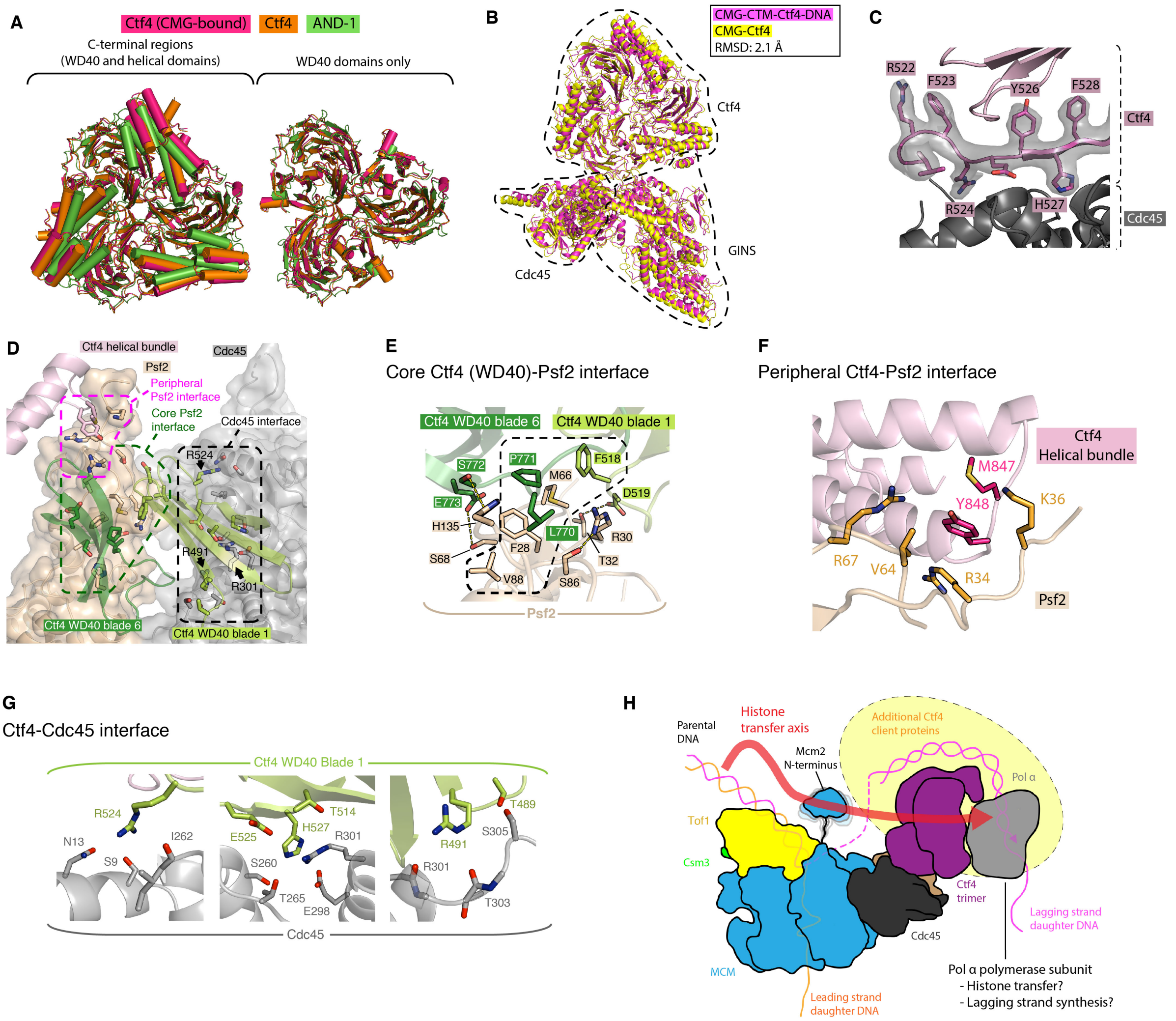

Figure S4, related to Figure 1. Ctf4 tethering in the replisome.
(A) Comparison of CMG-bound Ctf4 with previously published crystal structures of the C-terminal regions of trimeric Ctf4 (PDB: 4C8H) (Simon et al., 2014) and human And-1 (PDB: 5OGS) (Kilkenny et al., 2017).
(B) Comparison of the Cdc45-GINS-Ctf4 region of the CMG-Csm3/Tof1-Mrc1-Ctf4-DNA structure (conformation 1) with a prior structure of Ctf4 bound to a single CMG molecule in the absence of DNA (PDB: 6PTJ) (Yuan et al., 2019).
(C) Representative region of cryo-EM density at the CMG:Ctf4 interface, shown for a region of Ctf4 WD40 blade 1.
(D) Overview of the CMG-Ctf4 interface from a "top-down" perspective looking from Ctf4 towards Cdc45/GINS. Surface representations are shown for Cdc45/GINS. Only the regions of Ctf4 involved in the interaction are displayed. Core and peripheral interaction networks at the interface with Psf2 are expanded in (E) and (F), respectively. Arginine residues central to interaction networks at the interface with Cdc45 [expanded in (G)] are labelled.
(E) Ctf4-Psf2 core interface mediated by the Ctf4 WD40 domain. The central hydrophobic network is indicated by the dashed outline. Yellow dashes indicate hydrogen bonds.
(F) Ctf4-Psf2 peripheral interaction network mediated by the Ctf4 helical bundle.
(G) Ctf4-Cdc45 interface, subdivided into three interaction networks each involving a central arginine.

(H) Schematic illustrating the position of Ctf4 in the context of the eukaryotic replisome replicating DNA. The position of Pol α suggested by recent cryo-EM investigation of CMG-Ctf4 complexes in the absence of DNA is indicated (Yuan et al., 2019). The N-terminus of Mcm2 (not resolved) is also illustrated as flexibly tethered. The yellow halo indicates additional Ctf4 interacting partners ("clients") are expected to interact with the replisome, including Chl1, Dia2 and Dna2 (Morohashi et al., 2009; Villa et al., 2016). The role suggested of Ctf4 in parental histone transfer to the lagging strand along the Mcm2-Ctf4-Pol α axis is illustrated (Gan et al., 2018).

Figure S5

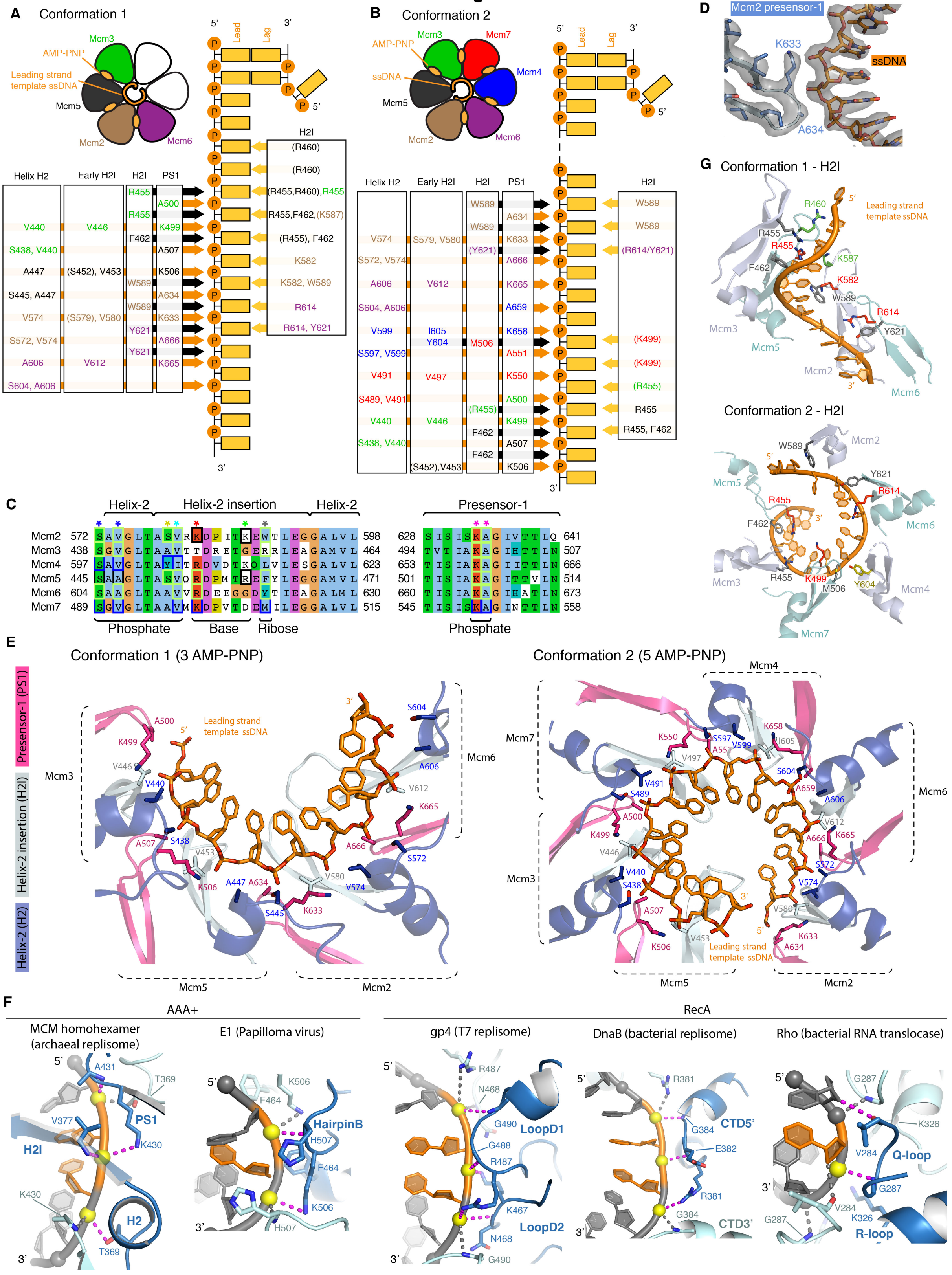

A
Conformation 1
Mcm3
AMP-PNP
Leading strand template ssDNA
Mcm5
Mcm2
Mcm6
Lead
Lag
5'
3'
Helix H2
Early H2I
H2I
PS1
B
Conformation 2
Mcm7
Mcm4
ssDNA
C
Helix-2
Helix-2 insertion
Presensor-1
Mcm2 572 SAVGLTASVRKDPITKEWTLEGGALVL 598
Mcm3 438 SGVGLTAAVTTDRETGERRLEAGAMVL 464
Mcm4 597 SAVGLTAYITRDVDTKQLVLESGALVL 623
Mcm5 445 SAAGLTASVQRDPMTREFYLEGGAMVL 471
Mcm6 604 SAAGLTAAVVRDEEGGDYTIEAGALML 630
Mcm7 489 SGVGLTAAVMKDPVTDEMILEGGALVL 515
Mcm2 628 SISISKAGIVTTLQ 641
Mcm3 494 TVTIAKAGIHTTLN 507
Mcm4 653 TISIAKAGIITTLN 666
Mcm5 501 TISIAKAGITTVLN 514
Mcm6 660 TISIAKAGIHATLN 673
Mcm7 545 TISISKAGINTTLN 558
Phosphate
Base
Ribose
D
Mcm2 presensor-1
K633
A634
ssDNA
E
Conformation 1 (3 AMP-PNP)
Conformation 2 (5 AMP-PNP)
Presensor-1 (PS1)
Helix-2 insertion (H2I)
Helix-2 (H2)
G
Conformation 1 - H2I
Conformation 2 - H2I
F
AAA+
RecA
MCM homohexamer (archaeal replisome)
E1 (Papilloma virus)
gp4 (T7 replisome)
DnaB (bacterial replisome)
Rho (bacterial RNA translocase)
PS1
H2I
H2
HairpinB
LoopD1
LoopD2
CTD5'
CTD3'
Q-loop
R-loop

Figure S5, related to Figure 3.
(A, B) Representation of contacts made by the MCM C-tier with leading-strand template ssDNA for conformations 1 and 2 respectively based on proximity. Arrows indicating interaction with phosphate (“P”), ribose and nucleobase moieties are colored orange, black and yellow, respectively. Parentheses denote residues where cryo-EM density could not unambiguously identify the position of side chains.
(C) Multiple sequence alignment for MCM C-tier DNA binding loops for *S. cerevisiae* MCM subunits. Boxes indicate residues involved in ssDNA interactions, with interactions observed in conformation 1, 2 or both colored black, blue or lime-green, respectively. Asterisks above the alignment are colored according to the scheme used in other panels.
(D) Cryo-EM density observed for C-tier-bound ssDNA and a representative interacting loop.
(E) Repetitive interactions of MCM C-tier AAA+ domains with the leading-strand template phosphate moieties.
(F) Comparison of repetitive phosphate contacts mediated by diverse homohexameric nucleic acid helicases (PDB: archaeal MCM, 6MII; E1, 2GXA; T7, 6N7N; DnaB, 4ESV; Rho, 5JJI) (Enemark and Joshua-Tor, 2006; Gao et al., 2019; Itsathitphaisarn et al., 2012; Meagher et al., 2019; Thomsen et al., 2016).
(G) Interactions of MCM H2I loop residues with sugar/base moieties of leading-strand template ssDNA. Residues are colored according to their position in the H2I primary sequence, corresponding to the panel (C) asterisks. Additional arginine residues in Mcm2/5 in conformation 1 (coloured green), and a tyrosine residue from Mcm4 in conformation 2 (colored yellow), at distinct positions in the H2I loop are also shown to contact ssDNA.

Figure S6

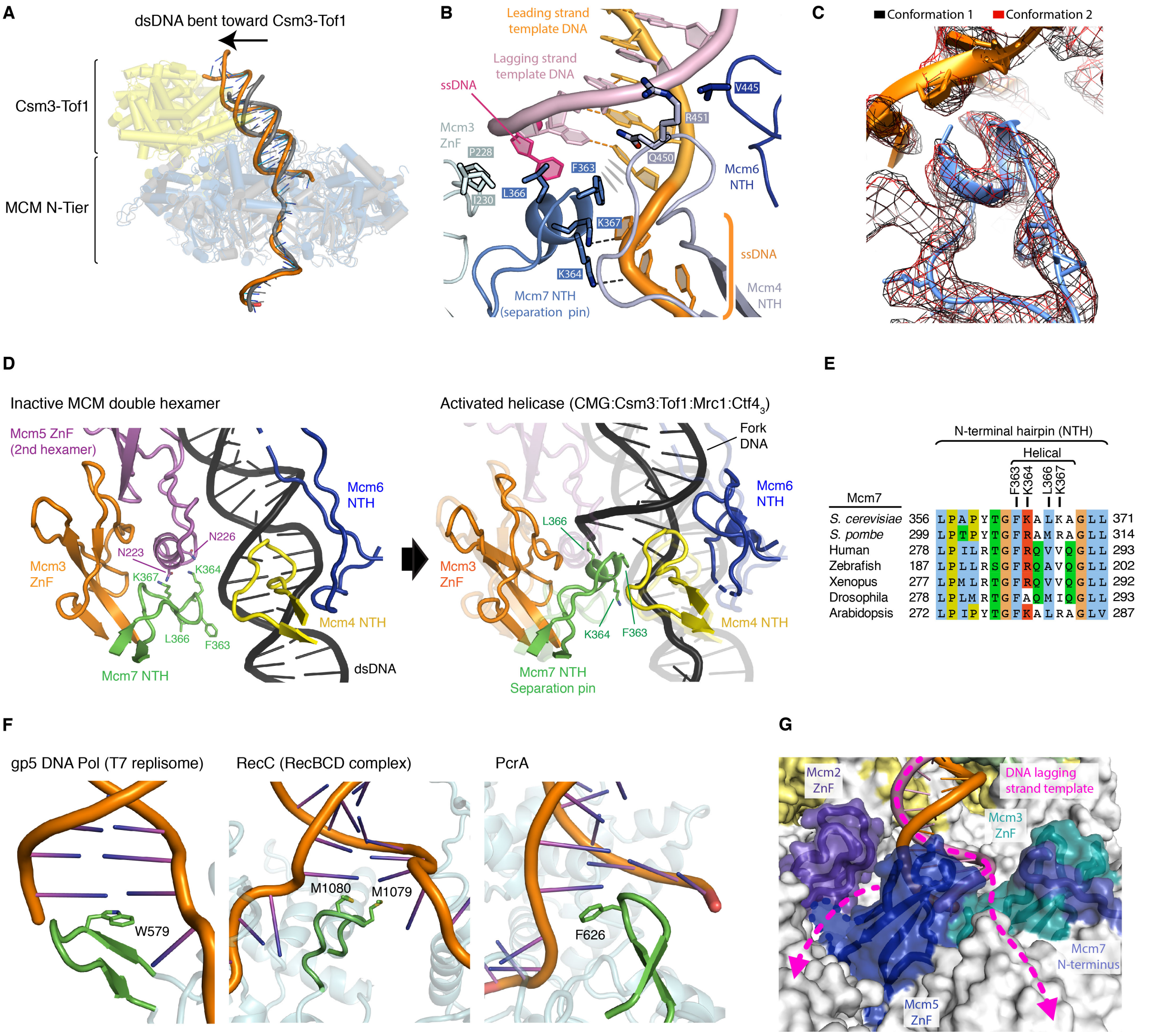

Figure S6, related to Figure 3.

(A) Superposition of the CMG-Ctf4-Csm3/Tof1-Mrc1-fork DNA structure (conformation 1, coloured blue [MCM], yellow [Csm3/Tof1], orange [DNA]) with a prior CMG-fork DNA structure (PDB: 5U8S, coloured grey) (Georgescu et al., 2017). For clarity only the Mcm N-tier, Csm3/Tof1 and DNA are shown. Models were aligned on the MCM N-Tier using PyMOL.

(B) Detailed view of the residues involved in interactions with DNA at the fork junction. The Mcm5 ZnF which contacts the unwound lagging strand via R184 is omitted for clarity (see Movie S2). F363 is shown to make π-π interactions with the last base-pair of DNA.

(C) Comparison of the Mcm7-NTH for conformations 1 and 2. Cryo-EM density is shown as mesh.

(D) Comparison of the MCM loops surrounding the fork junction in CMG with the corresponding region of the inactive MCM double hexamer on dsDNA (PDB: 5BK4) (Noguchi et al., 2017). Right-hand side: the model for the active helicase is shown overlaid on the model of the inactive double hexamer having aligned globally on the MCM N-tier, for ease of comparison.

(E) Multiple sequence alignment for the Mcm7 N-terminal hairpin (strand separation pin). The internal helix and DNA-interaction residues are labelled.
(F) Comparison of previously described strand separation pins from diverse helicases (PDB: T7, 6N7W; RecBCD, 1W36; PcrA, 3PJR) (Gao et al., 2019; Singleton et al., 2004; Velankar et al., 1999). The characteristic hydrophobic residues observed to abut the last base-pair are indicated.
(G) Schematic for hypothesized lagging-strand template ssDNA exit pathways post-unwinding.

Figure S7

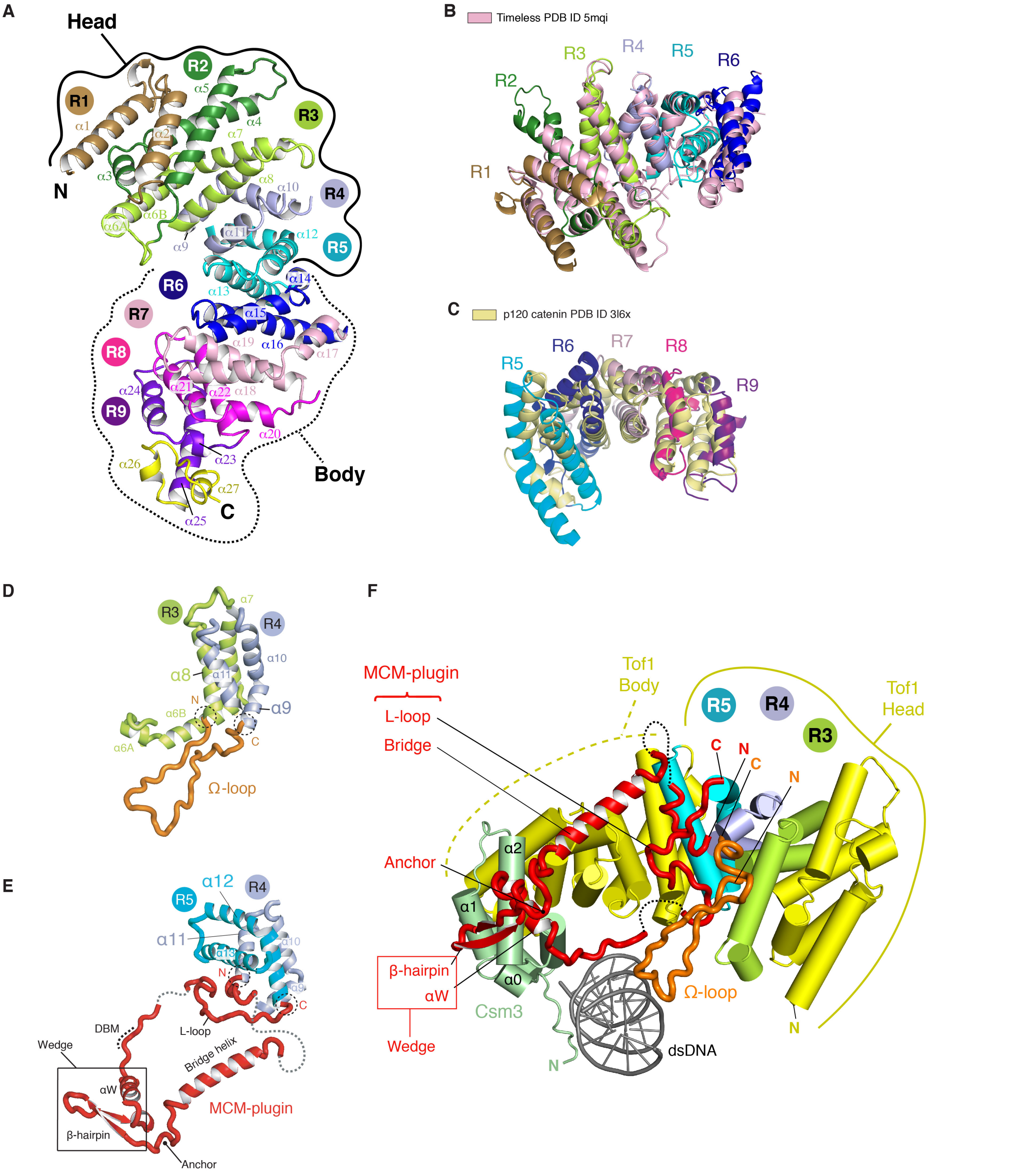

Figure S7, related to Figure 4.
(A) Assignment of Tof1 helical repeats. The model is coloured by helical repeat (R1-9) and the α-helices are numbered. The positions of the Head (repeats 1-5) and Body (repeats 6-9) are highlighted.
(B) Superposition of Tof1 helical repeats 1-6 with the crystal structure of the human Timeless N-terminus (Holzer et al., 2017) (RMSD = 2 Å).
(C) Superposition of Tof1 helical repeats 5-9 with repeats 5-9 of p120 Catenin (Ishiyama et al., 2010) (RMSD = 6 Å). p120 Catenin was returned as the second highest scoring protein after Timeless in a Dali search (Holm and Sander, 1995) against Tof1 (13-781).

(D) Location of the Tof1 Ω-loop between helical repeats 3 and 4.
(E) Location of the MCM-plugin between helical repeats 4 and 5. The N- and C-termini of the Ω-loop and MCM-plugin are highlighted by dashed black circles.
(F) Bottom view of Csm3/Tof1. Tof1 repeats 3, 4 and 5 are highlighted to emphasize the positions of the Ω-loop and MCM-plugin.

Figure S8

A Tof1 Ω-loop and MCM-plugin

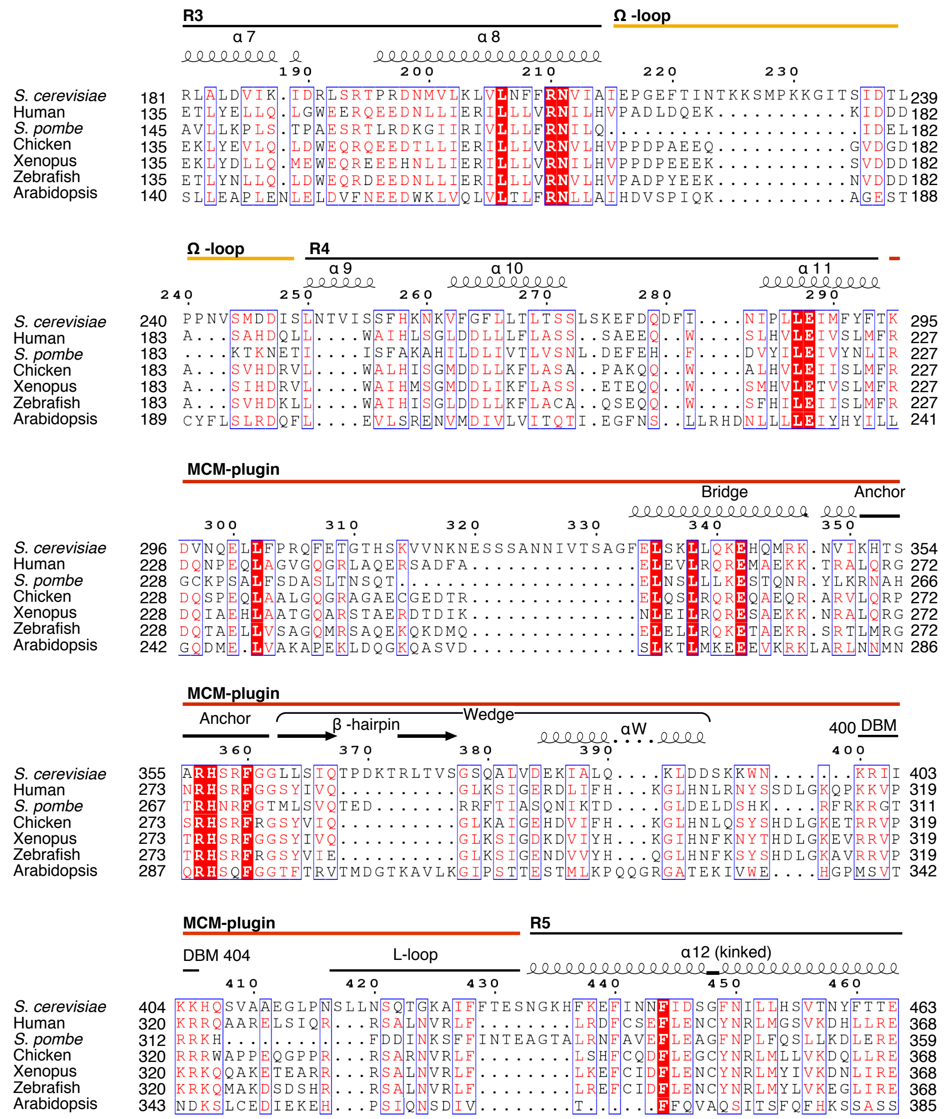

B Csm3 (model built for 46-139)

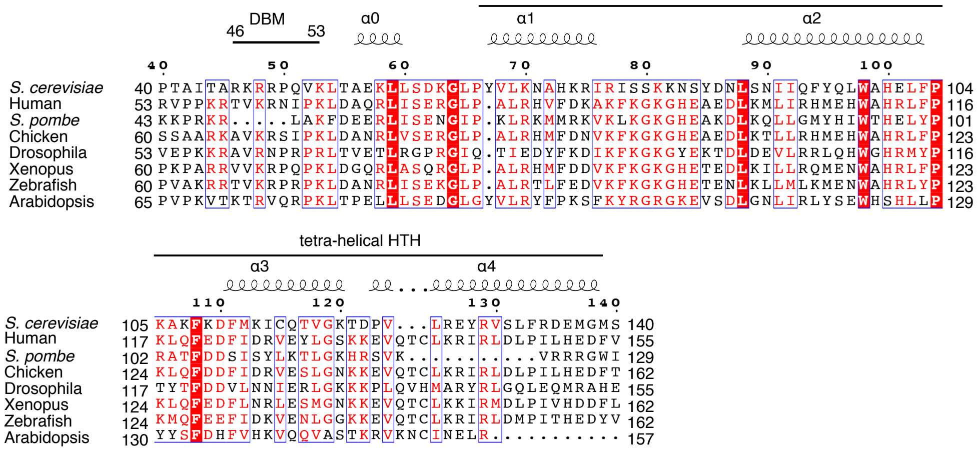

Figure S8, related to Figure 4. Multiple sequence alignment of Csm3 and Tof1.
(A) The sequences of Tof1 (*S. cerevisiae*, 1238 residues, SGD YNL273W) and its orthologues: human Timeless (*H. sapiens*, 1208 residues, UniProtKB Q9UNS1), Swi1 (*S. pombe*, 971 residues, UniProtKB Q9UUM2), chicken Timeless (*G. gallus*, 1217 residues, NCBI ref XP_015155764.1), Xenopus Timeless (*X. tropicalis,* 1204 residues, UniProtKB F6Z6K7), zebrafish Timeless (*D. rerio*, 1278 residues, NCBI ref NP_001265529.1) and Arabidopsis Timeless *(A. thaliana*, 1141 residues, NCBI ref NP_200103.1).
(B) The sequence of Csm3 (*S. cerevisiae*, 317 residues, SGD YMR048W) and its orthologues: human Tipin (*H. sapiens*, 301 residues, UniProtKB Q9BVW5), Swi3 (*S. pombe*, 181 residues, UniProtKB O14350), chicken Tipin (*G. gallus*, 283 residues, UniProtKB Q5F416), Xenopus Tipin (*X. laevis,* 368 residues, UniProtKB Q0IHI4), zebrafish Tipin (*D. rerio*, 294 residues, UniProtKB G1K2L6) and Arabidopsis Tipin (*A. lyrata*, 287 residues, UniProtKB D7KZL5).

Figure S9

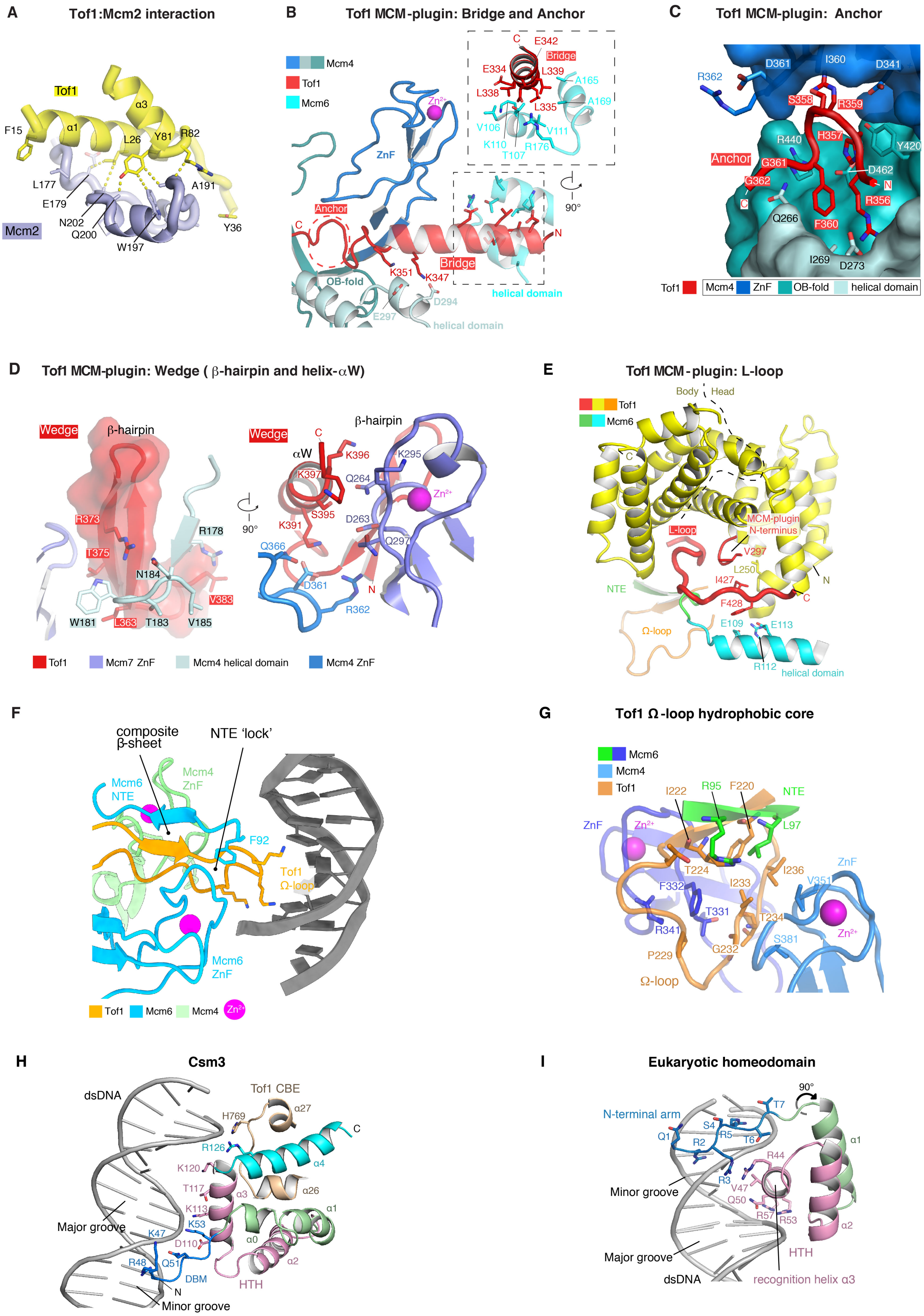

A
Tof1:Mcm2 interaction
Tof1
α3
α1
F15
L26
Y81
R82
L177
E179
A191
N202
Q200
W197
Y36
Mcm2
B
Tof1 MCM-plugin: Bridge and Anchor
Mcm4
Tof1
Mcm6
E342
C
Bridge
E334
L339
A165
L338
L335
A169
V106
V111
K110
R176
T107
Zn2+
ZnF
90°
Anchor
C
N
K351
K347
Bridge
OB-fold
D294
E297
helical domain
helical domain
C
Tof1 MCM-plugin: Anchor
D361
I360
D341
R362
S358
R359
R440
H357
Y420
Anchor
G361
D462
G362
N
C
R356
Q266
F360
I269
D273
Tof1
Mcm4
ZnF
OB-fold
helical domain
D
Tof1 MCM-plugin: Wedge (β-hairpin and helix-αW)
Wedge
β-hairpin
R373
R178
T375
N184
V383
W181
L363
T183
V185
90°
Wedge
β-hairpin
C
αW
K396
K295
K397
Q264
Zn2+
S395
D263
K391
Q366
Q297
D361
N
R362
Tof1
Mcm7 ZnF
Mcm4 helical domain
Mcm4 ZnF
E
Tof1 MCM-plugin: L-loop
Body
Head
Tof1
Mcm6
C
L-loop
MCM-plugin
N-terminus
V297
L250
NTE
I427
N
F428
C
Ω-loop
E109
E113
R112
helical domain
F
composite
β-sheet
NTE 'lock'
Mcm6
NTE
Mcm4
ZnF
F92
Tof1
Ω-loop
Mcm6
ZnF
Tof1
Mcm6
Mcm4
Zn2+
G
Tof1 Ω-loop hydrophobic core
Mcm6
Mcm4
Tof1
I222
R95
F220
NTE
ZnF
Zn2+
L97
ZnF
T224
I236
F332
I233
V351
T331
T234
R341
S381
Zn2+
P229
G232
Ω-loop
H
Csm3
dsDNA
Tof1 CBE
H769
α27
C
R126
α4
K120
T117
α3
α26
K113
α1
Major groove
K47
K53
α0
D110
R48
Q51
α2
DBM
HTH
N
Minor groove
I
Eukaryotic homeodomain
90°
N-terminal arm
T7
S4
Q1
R5
T6
α1
R2
R44
R3
Minor groove
V47
Q50
R57
R53
α2
HTH
Major groove
dsDNA
recognition helix α3

Figure S9, related to Figure 5.

(A) Interface between Tof1 (α1 and α3) and the Mcm2 NTE.

(B) Detailed view of interactions between the Bridge and Mcm4 and 6. The position of the Anchor is highlighted with a dashed red line and the inset shows details of the Bridge interface with the Mcm6 helical domain.

(C) Details of Anchor binding sites on Mcm4. The Mcm4 ZnF, OB-fold and helical domain are denoted by different shades of blue.

(D) Details of Wedge binding to Mcm4 and 7.

(E) Interactions of the L-loop with the Mcm6 helical domain and NTE. The dashed black line denotes the boundary of the Tof1 Head and Body.

(F) Detailed view of Ω-loop interactions with Mcm6, Mcm4 and dsDNA illustrating the 'lock' formed by the Mcm6 ZnF and NTE.

(G) Details of the interactions between the hydrophobic core of the Ω-loop and Mcm6 and 4.

(H) Overview of the Csm3 structure. Amino acid side chains for residues in the DBM and close to dsDNA are shown.

(I) Binding of a typical homeodomain transcription factor to dsDNA (Drosophila paired protein, PDB: 1FJL) (Wilson et al., 1995) emphasizing the minor groove contacts made by the N-terminal arm.

For (H) and (I), corresponding regions of the structures are colored similarly.

DBM, DNA binding motif.

Figure S10

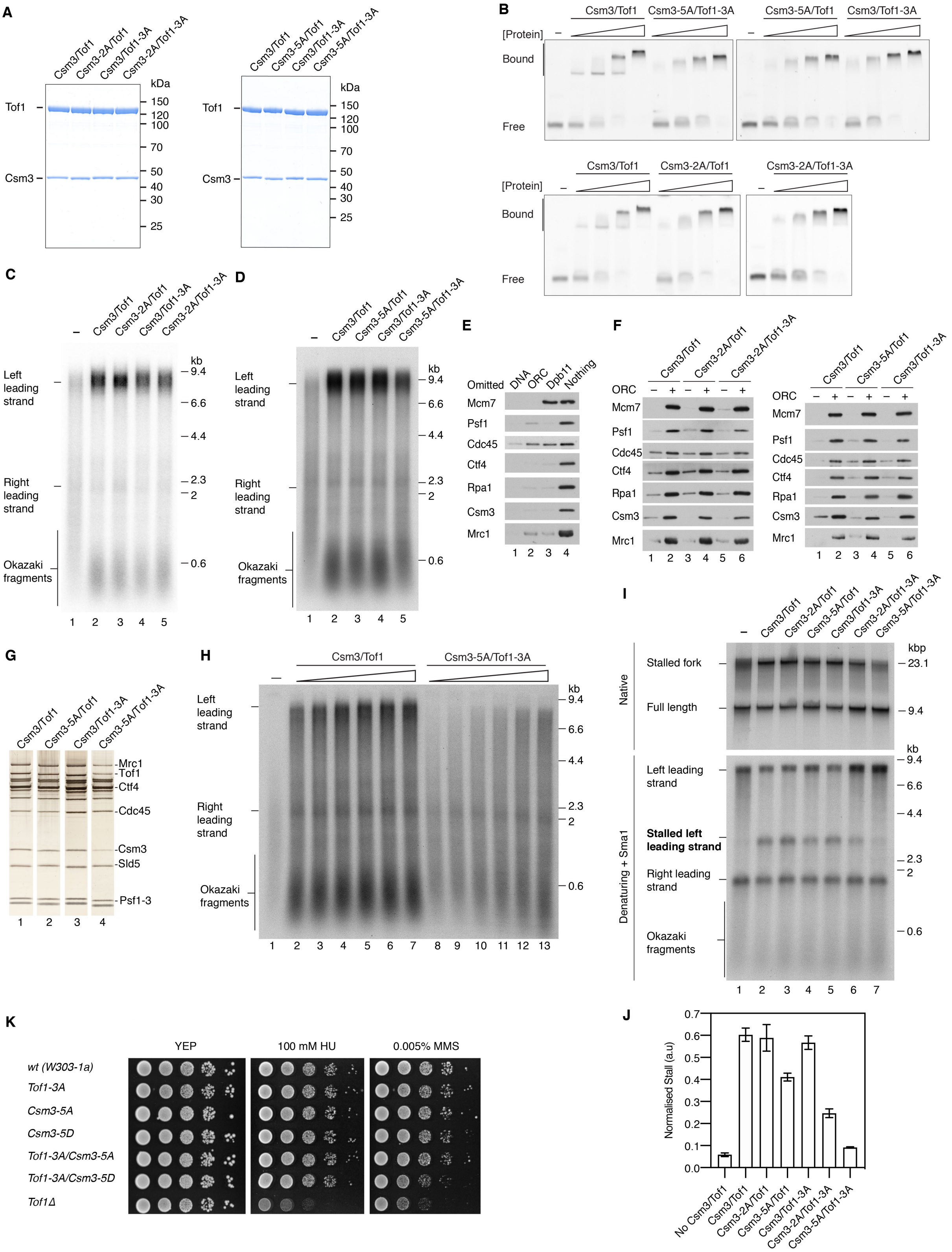

Figure S10, related to Figures 6 and 7.

(A) Coomassie strained SDS-PAGE of Csm3/Tof1 DBM mutants.

(B) Electrophoretic mobility shift assays (EMSA) with the fork DNA substrate in Figure S1A and increasing concentrations (40, 80, 160, 320 nM) of the indicated Csm3/Tof1 proteins.

(C, D) Denaturing gel analysis of origin-dependent replication reactions performed for 15 (C) and 20 (D) min.

(E, F) Protein association assays performed as in Figure 6D.
(G) Peak fractions from native glycerol gradients performed as in Figure S1B with the indicated Csm3/Tof1 proteins.
(H) Denaturing gel analysis of an origin-dependent replication reaction performed for 7 min with increasing concentrations (2.5, 5, 10, 20, 40, 80 nM) of the indicated Csm3/Tof1 proteins.
(I) Experiment performed and analyzed as in Figure 7B but with 100 mM potassium glutamate for 30 min. Pol δ was omitted as it catalyzes extensive strand-displacement synthesis in lower ionic strength buffers (Devbhandari et al., 2017).
(J) Quantitation of experiments performed as in (I). Error bars represent the SEM from a minimum of 3 experiments.
(K) Spot dilution assay performed for 2 days at 30°C.
SEM, standard error of the mean.

Subunit	Chain ID	Region	Residues	Residues built	Model template	Model building
**Csm3/Tof1**						
Tof1	X	repeats 1-6	1-213, 250-294, 433-550	13-37, 43-106, 117-213, 250-274, 280-294, 433-550	PDB: 5MQI (Holzer *et al.*, 2017)	Homology model docked and rebuilt
		Ω-loop	214-249	214-249	-	*de novo*
		MCM-plugin	295-432	295-305, 329-405, 412-432		
		repeats 7-8	551-704	556-606, 657-704		
		CBE	705-781	705-781		
Csm3	Y	N-terminus (DBM, α0)	1-63	46-63	-	*de novo*
		tetrahelical-HTH (α1- α4)	64-139	64-139		
**CMG**						
Mcm2	2	N-tier	173-471	173-471	PDB: 5U8S (Georgescu *et al.*, 2017)	Docked and rebuilt
		C-tier	472-868	472-710, 738-868		
Mcm3	3	N-tier	18-336	18-56, 90-332		Docked and rebuilt
		C-tier	337-740	337-583, 648-689, 696-740		
Mcm4	4	N-tier	174-505	174-469		Docked and rebuilt
		C-tier	505-852	505-552, 557-607, 615-731, 741-780, 792-835, 844-852		
		WH	853-933	853-920, 922-927		Docked and rebuilt
Mcm5	5	N-tier	20-343	20-107, 131-198, 205-213, 235-305, 320-343		Docked and rebuilt
		C-tier	344-694	344-694		
Mcm6	6	N-tier	91-496	91-200, 255-419, 434-463		Docked and rebuilt
		C-tier	497-838	497-615, 620-737, 744-838		
Mcm7	7	N-tier	1-395	4-34, 60-157, 190-210, 219-385		Docked and rebuilt
		C-tier	396-730	396-443, 449-486, 493-673, 679-729		
Psf1	A	-	1-208	1-107, 119-208		Docked and rebuilt
Psf2	B	-	1-213	1-38, 47-201		Docked and rebuilt
Psf3	C	-	1-194	1-58, 68-144, 158-194		Docked and rebuilt

		N-terminal His-tag	(-22) - 0	(-4) - 0		*de novo*
Sld5	D	-	1-294	2-15, 54-107, 120-294		Docked and rebuilt
		CIP-box	2-13	2-13		Docked and rebuilt using PDB: 4C95 (Simon *et al.*, 2014)
Cdc45	E	-	1-650	1-108, 111-166, 223-436, 461-593, 598-650		Docked and rebuilt
Ctf4 trimer						
Ctf4 monomer (CMG interface)	F			473-664, 670-791, 814-923		Docked and rebuilt
Ctf4 monomer (GINS-facing)	G	C-terminal regions	473-927	474-663, 670-790, 814-924	PDB: 4C8H (Simon *et al.*, 2014)	Docked and rebuilt
Ctf4 monomer (Cdc45-facing)	H			473-665, 670-790, 814-924		Docked and rebuilt
Fork DNA						
Leading-strand template	I	-	1-85	26-62	-	*de novo*
Lagging-strand template	J	-	1-61	15-36	-	*de novo*
Ligands						
	2	Mcm2/6 interface				
AMPPNP; Mg^{2+}	3	Mcm3/5 interface	-	1500; 1501	-	*de novo*
	5	Mcm5/2 interface				
	2	Mcm2 ZnF				
	4	Mcm4 ZnF				
Zn^{2+}	5	Mcm5 ZnF	-	1400	-	*de novo*
	6	Mcm6 ZnF				
	7	Mcm7 ZnF				

Table S1, related to STAR Methods. Summary of cryo-EM model building for conformation 1. See Methods for further details. For CMG and Ctf4, region boundaries were approximated from the structure.

Name	Construction details	Application
vJY23	pRS304-Psf1-Gal1-10-Sld5	Psf1 / Sld5
vJY24	pRS306-Psf2-Gal1-10-His-Psf3	Psf2 / His-Psf3
vJY25	pRS303-Fob1-TEV-2xFLAG- Gal1-10-Gal4	Fob1
vJY30	A 243 bp fragment containing the yeast RFB from chromosome XII was amplified with JY204 – TTCACTGTTCCTGCAGCACTTGTCTCTTACATCTTTCTTGG / JY205-TTTTTTTTTTGGATCCGTTGCAAAGATGGGTTGAAAG and cloned into ZN5(Taylor and Yeeles, 2018) using BamHI and PstI. RFB sequence:<br>GTTGCAAAGATGGGTTGAAAGAGAAGGGCTTTCACAAAGCTTCCCGAGCGTG<br>AAAGGATTTGCCCGGACAGTTTGCTTCATGGAGCAGTTTTTTCCGCACCATCA<br>GAGCGGCAAACATGAGTGCTTGTATAAGTTTAGAGAATTGAGAAAAGCTCATT<br>TCCTATAGTTAACAGGACATGCCTTTGATATGAAAAAAAATACTACGAACTAC<br>GATTTTACCAAGAAAGATGTAAGAGACAAGTG	RFB template
vJY71	pRS303-Cdc45^{iF2}-Gal1-10-Ctf4	Cdc45^{iF2} / Ctf4
vJY72	pRS based vector. Tof1-Gal1-10-Csm3 cloned Not1 / Apa1. Auxotrophic marker from pRS vector replaced with Nat-NT2 resistance marker amplified from pBP83 (Yeeles et al., 2015) with oligos (JY247 - TCTAGTCAATGCGGCCGCCGTACGCTGCAGGTCGAC and JY248 - ATCGATGAATTCGAGCTCG) and cloned Zra1/Not1. Lys2 3321-3799 amplified with primers (JY253 – CCGATTGACAGGGCCCTTCCTCCAC TTCTACTCTTGACAC and JY254 – CCGATTGACAAAGCGGAAGAGCCGAC ATCGTAACCATAATCGTG) and cloned Sap1/Apa1.	Csm3 / Tof1
vJY74	pRS based vector. Mrc1-Gal1-10-Gal4 cloned Not1 / Apa1. Auxotrophic marker from pRS vector replaced with Nat-NT2 resistance marker amplified from pBP83 (Yeeles et al., 2015) with oligos (JY247 and JY248) and cloned Zra1 / Not1. Lys2 3321-3799 amplified with primers (JY253 and JY254) and cloned Sap1/Apa1.	Mrc1
vJY111	pRS303-Rfa1-Gal1-10-Gal4	RPA
vJY113	pRS306 Tof1-gal1-10-CBP-Csm3$^{R49A, K53A}$<br>PCR mutagenesis with JY344 – AGTTGCTTTGACCGCTGAAAAGTTGTTG / 345 - TGTGGAGCTCTCTTTCTAGCGGTG	Csm3$^{R49A, K53A}$/Tof1
vJY114	pRS306 Tof1$^{K400A, R401A, K404A}$-gal1-10-CBP-Csm3<br>PCR mutagenesis with JY347 - ATTGCTAAGCACCAATCCGTTGCTG / JY348 - AATAGCAGCGTTCCACTTCTTAGAATCATCC from pRS306/Tof1- Gal-CBP-Csm3	Csm3/ Tof1$^{K400A, R401A, K404A}$
vJY115	pRS306 Tof1$^{K400A, R401A, K404A}$-gal1-10-CBP-Csm3$^{R49A, K53A}$<br>Subclone Csm3$^{R49A, K53A}$ into vJY114 with AscI/XhoI	Csm3$^{R49A, K53A}$/Tof1 $^{K400A, R401A, K404A}$
vJY116	pRS306 Tof1-gal1-10-CBP-Csm3$^{K47A, R48A, R49A, Q51A, K53A}$<br>PCR mutagenesis with JY377 - GCTCCAGCTGTTGCTTTGACCG / JY378 - AGCAGCTCTAGCGGTGATAGCAGTTG from pRS306/Tof1- Gal-CBP-Csm3	Csm3$^{K47A, R48A, R49A, Q51A, K53A}$/Tof1
vJY117	pRS306 Tof1$^{K400A, R401A, K404A}$-gal1-10-CBP-Csm3$^{K47A, R48A, R49A, Q51A, K53A}$<br>PCR mutagenesis with JY377/JY378 from vJY114	Csm3$^{K47A, R48A, R49A, Q51A, K53A}$/ Tof1$^{K400A, R401A, K404A}$
vVA30	The Tof1 open reading frame was amplified from W303 genomic DNA with primers VA84- TTCAATATAAGTCGACGCCCTGTATGAATTGCTTCC and vVA85 - TTCAATATAAGGCGCGCCATAGTCCTAGTAGCGGATTGC and was cloned into pFA6-Ura with AscI / SalI.	Parent vector for Tof1 mutagenesis
vVA31	The Tof1-3A open reading frame was amplified with primers vVA84 and vVA85 and was cloned into pFA6-Ura with AscI / SalI.	Construction of Tof1-3A strains
vVA32	The Csm3 open reading frame was amplified from W303 genomic DNA with primers VA81 - TTCAATATAAGTCGACATGGATCAAGATTTTGACAGTTTATTAC and VA82 - TTCAATATAAGGCGCGCCCTAAAAGCCCATTTCCTTCATAGC and was cloned into pFA6-Ura with AscI / SalI.	Parent vector for Csm3 mutagenesis
vJY136	PCR mutagenesis form vVA32 with JY422 – GATCCTCAAGTAGATTTAACA GCCGAAAAAC and JY423 - GTCATCATCAGCTGTAATTGCGGTTGGATC	Construction of Csm3-5D strains

vJY137	PCR mutagenesis form vVA32 with JY397 – CCTGCTGTAGCATTAACAGCCGA AAAACTACTCAG and JY398 - CGCTGCAGCTCTAGCTGTAATTGCGGTTGG	Construction of Csm3-5A strains

Table S3, related to STAR Methods. Details of vectors used and their construction.

Strain	Genotype	Reference
yJY36	*MATα ade2-1 ura3-1 his3-11,15 trp1-1 leu2-3,112 can1-100*<br>*bar1::Hyg*<br>*pep4::KanMX*<br>*ura3::URA3pRS306-Psf2/His-Psf3 (vJY24)*<br>*trp1::TRP1pRS304Psf1/Sld5 (vJY23)*<br>*his::HISpRS303Cdc45iFlag2/Gal4*	This study
yAM22	*MATa ade2-1 ura3-1 his3-11,15 trp1-1 leu2-3,112 can1-100*<br>*bar1::Hyg*<br>*pep4::KanMX*<br>*trp1::TRP1pRS304Mcm4, Mcm5*<br>*ura3::URA3pRS306/Mcm2, CBP-TEV Mcm3*<br>*leu2::LEU2pRS305/Mcm6, Mcm7*	(Zhou et al., 2017)
yJY37	*MATa / MATα ade2-1 ura3-1 his3-11,15 trp1-1 leu2-3,112 can1-100*<br>*bar1::Hyg*<br>*pep4::KanMX*<br>*trp1::TRP1pRS304Mcm4, Mcm5*<br>*trp1::TRP1pRS304Psf1/Sld5 (vJY23)*<br>*ura3::URA3pRS306/Mcm2, CBP-TEV Mcm3*<br>*ura3::URA3pRS306-Psf2/His-Psf3 (vJY24)*<br>*leu2::LEU2pRS305/Mcm6, Mcm7*<br>*his::HISpRS303Cdc45iFlag2/Gal4*	This study
yJY39	*MATa ade2-1 ura3-1 his3-11,15 trp1-1 leu2-3,112 can1-100*<br>*bar1::Hyg*<br>*pep4::KanMX*<br>*his3::HIS3pRS303-Fob1-TEV-2xFLAG- Gal1-10-Gal4 (vJY25)*	This study
yJY72	*MATα ade2-1 ura3-1 his3-11,15 trp1-1 leu2-3,112 can1-100*<br>*bar1::Hyg*<br>*pep4::KanMX*<br>*ura3::URA3pRS306-Psf2/His-Psf3 (vJY23)*<br>*trp1::TRP1pRS304Psf1/Sld5 (vJY24)*<br>*his::HISpRS303Cdc45iFlag2/untagged Ctf4 (vJY71)*<br>*lys2::NAT-untagged Mrc1 (vJY74)*	This study
yJY69	*MATa ade2-1 ura3-1 his3-11,15 trp1-1 leu2-3,112 can1-100*<br>*bar1::Hyg*<br>*pep4::KanMX*<br>*trp1::TRP1pRS304Mcm4, Mcm5*<br>*ura3::URA3pRS306/Mcm2, CBP-TEV Mcm3*<br>*leu2::LEU2pRS305/Mcm6, Mcm7*<br>*lys2::NAT- untagged Csm3/Tof1 (vJY72)*	This study
yJY74	*MATa /MATα ade2-1 ura3-1 his3-11,15 trp1-1 leu2-3,112 can1-100*<br>*bar1::Hyg*<br>*pep4::KanMX*<br>*trp1::TRP1pRS304Mcm4, Mcm5*<br>*trp1::TRP1pRS304Psf1/Sld5 (vJY23)*<br>*ura3::URA3pRS306/Mcm2, CBP-TEV Mcm3*<br>*ura3::URA3pRS306-Psf2/His-Psf3 (vJY24)*<br>*leu2::LEU2pRS305/Mcm6, Mcm7*<br>*his::HISpRS303Cdc45iFlag2/untagged Ctf4 (vJY71)*<br>*lys2::NAT-untagged Csm3/Tof1 (vJY72)*<br>*lys2::NAT-untagged Mrc1 (vJY74)*	This study
yJY106	*MATa ade2-1 ura3-1 his3-11,15 trp1-1 leu2-3,112 can1-100*<br>*bar1::Hyg*<br>*pep4::KanMX*<br>*ura3::URA3pRS306/Rfa2,Rfa3*<br>*his::HISpRS303Untagged RPA1 (vJY111)*	This study
yJY110	*MATα ade2-1 ura3-1 his3-11,15 trp1-1 leu2-3,112 can1-100*<br>*bar1::Hyg*<br>*pep4::KanMX*<br>*URA::Ura pRS306 Tof1-gal1-10-CBP-Csm3*$^{R49A / K53A}$ *(vJY113)*	This study
yJY111	*MATα ade2-1 ura3-1 his3-11,15 trp1-1 leu2-3,112 can1-100*<br>*bar1::Hyg*	This study

	pep4::KanMX<br>*URA::Ura pRS306 Tof1*$^{K400,\ R401A,\ K404A}$*-gal1-10-CBP-Csm3 (vJY114)*	
yJY112	*MAT**α** ade2-1 ura3-1 his3-11,15 trp1-1 leu2-3,112 can1-100*<br>*bar1::Hyg*<br>*pep4::KanMX*<br>*URA::Ura pRS306 Tof1*$^{K400A,\ R401A,\ K404A}$*-gal1-10-CBP-Csm3*$^{R49A,\ K53A}$ *(vJY115)*	This study
yJY120	*MAT**a** ade2-1 ura3-1 his3-11,15 trp1-1 leu2-3,112 can1-100*<br>*bar1::Hyg*<br>*pep4::KanMX*<br>*URA::Ura pRS306 Tof1-gal1-10-CBP-Csm3*$^{K47A,\ R48A,\ R49A,\ Q51A,\ K53A}$ *(vJY116)*	This study
yJY121	*MAT**a** ade2-1 ura3-1 his3-11,15 trp1-1 leu2-3,112 can1-100*<br>*bar1::Hyg*<br>*pep4::KanMX*<br>*URA::Ura pRS306 Tof1*$^{K400A,\ R401A,\ K404A}$*-gal1-10-CBP-Csm3*$^{K47A,\ R48A,\ R49A,\ Q51A,\ K53A}$<br>*(vJY117)*	This study
W303-1a	*MAT**a** ade2-1 ura3-1 his3-11,15 trp1-1 leu2-3,112 can1-100*	Lab strain constructed by<br>R. Rothstein
yJY145	*MAT**a** ade2-1 ura3-1 his3-11,15 trp1-1 leu2-3,112 can1-100*<br>*Csm3 (Ura3)*	This study
yVA57	*MAT**a** ade2-1 ura3-1 his3-11,15 trp1-1 leu2-3,112 can1-100*<br>*Tof1-3A (K400A, R401A, K404A)*	This study
yVA67	*MAT**a** ade2-1 ura3-1 his3-11,15 trp1-1 leu2-3,112 can1-100*<br>*Csm3-5D (R46D, K47D, R48D, R49D, K53D) (Ura3)*	This study
yVA68	*MAT**a** ade2-1 ura3-1 his3-11,15 trp1-1 leu2-3,112 can1-100*<br>*Csm3-5A (K47A / R48A / R49A / Q51A / K53A) (Ura3)*	This study
yVA70	*MAT**a** ade2-1 ura3-1 his3-11,15 trp1-1 leu2-3,112 can1-100*<br>*Tof1-3A*<br>*Csm3-5D (R46D, K47D, R48D, R49D, K53D) (Ura3)*	This study
yVA73	*MAT**a** ade2-1 ura3-1 his3-11,15 trp1-1 leu2-3,112 can1-100*<br>*Tof1-3A*<br>*Csm3-5A (K47A, R48A, R49A, Q51A, K53A) (Ura3)*	This study
yBH77	*MAT**a** ade2-1 ura3-1 his3-11,15 trp1-1 leu2-3,112 can1-100*<br>*tof1Δ::hphNT*	(Hodgson et al., 2007)

Table S4, related to STAR Methods. Details of yeast strains.

Protein	Expression strain	Affinity tag	Purification steps
Cdc45	yJY13	Internal 2xFLAG tag	Anti-FLAG M2 Agarose<br>Bio-Gel HT Hydroxyapatite
Cdc6	Plasmid pAM3 (*E. coli* expression)	N-terminal GST cleavable tag	Glutathione Sepharose 4B<br>Bio-Gel HT Hydroxyapatite
Cdt1-Mcm2-7	yAM33	N-terminal CBP cleavable tag on Mcm3	Calmodulin-Sepharose 4B<br>Superdex 200
Csm3/Tof1	yAE48	N-terminal CBP cleavable tag on Csm3	Calmodulin-Sepharose 4B<br>MonoQ<br>Superdex 200
Ctf4	yAE40	N-terminal CBP tag	Calmodulin-Sepharose 4B<br>MonoQ<br>Superdex 200
DDK	ySDK8	CBP tag on Dbf4	Calmodulin-Sepharose 4B<br>Lambda phosphatase dephosphorylation<br>Superdex 200
Dpb11	yJY26	C-terminal 3xFLAG tag	Anti-FLAG M2 Agarose<br>MonoS
GINS	Plasmid pJFDJ5 (*E. coli* expression)	N-terminal His tag on Psf3	Ni-NTA Agarose<br>MonoQ<br>Superdex 200
Mcm10	pET28a-Mcm10 (*E. coli* expression)	N-terminal His tag	Ni-NTA Agarose<br>MonoS (twice)
Mrc1	yJY32	C-terminal 2xFLAG tag	Anti-FLAG M2 Agarose<br>Superose 6
ORC	ySD-ORC	CBP-cleavable tag on Orc1	Calmodulin-Sepharose 4B<br>Superdex 200
PCNA	vJY19 (*E. coli* expression)	Untagged	Nucleic acid precipitation with Polymin P<br>Ammonium sulfate precipitation<br>HiTrap SP HP (flow through)<br>HiTrap Heparin HP (flow through)<br>HiTrap DEAE Fast Flow<br>MonoQ
Pol α	yAE95	N-terminal CBP tag on Pri1	Calmodulin-Sepharose 4B<br>MonoQ<br>Superdex 200
Pol δ	yAE34	C-terminal CBP tag on Pol32	Calmodulin-Sepharose 4B<br>HiTrap Heparin HP<br>Superdex 200
Pol ε	yAJ2	C-terminal CBP tag on Dpb4	Calmodulin-Sepharose 4B<br>HiTrap Heparin HP<br>Superdex 200
RFC	yAE41	N-terminal CBP tag on Rfc3	Calmodulin-Sepharose 4B<br>MonoS<br>Superdex 200
RPA	yJY106	Untagged	Nucleic acid precipitation with Polymin P<br>Ammonium sulfate precipitation<br>HiTrap Blue HP<br>ssDNA Cellulose<br>MonoQ

Sld2	yTD8	C-terminal 3x FLAG	Ammonium sulfate precipitation<br>Anti-FLAG M2 Agarose<br>HiTrap SP HP
Sld3/7	yTD6	C-terminal cleavable TCP tag	IgG Sepharose Fast Flow<br>TEV removal with Ni-NTA Agarose<br>Superdex 200
CMG	yJY37	Internal 2xFLAG tag on Cdc45<br>N-terminal CBP cleavable tag on Mcm3<br>N-terminal His tag on Psf3	Anti-FLAG M2 Agarose<br>Calmodulin-Sepharose 4B<br>MonoQ
Fob1	yJY39	C-terminal TEV 2x FLAG	Anti-FLAG M2 Agarose<br>MonoQ
CMG-Ctf4-Csm3/Tof1-Mrc1	yJY74	Internal 2xFLAG tag on Cdc45<br>N-terminal CBP cleavable tag on Mcm3<br>N-terminal His tag on Psf3	Anti-FLAG M2 Agarose<br>Calmodulin-Sepharose 4B<br>Glycerol gradient

Table S5, related to STAR Methods. Details of protein purification strategy.
